# Small-molecule-catalysed deamination enables transcriptome-wide profiling of *N*^6^-methyladenosine in RNA

**DOI:** 10.1038/s41557-025-01801-3

**Published:** 2025-04-17

**Authors:** Pingluan Wang, Chang Ye, Michelle Zhao, Bochen Jiang, Chuan He

**Affiliations:** 1 Department of Chemistry, The University of Chicago, Chicago, IL, USA.; 2 Howard Hughes Medical Institute, The University of Chicago, Chicago, IL, USA.; 3 USA Institute for Biophysical Dynamics, The University of Chicago, Chicago, IL, USA.; 4 Department of Biochemistry and Molecular Biology, The University of Chicago, Chicago, IL, USA.

## Abstract

The deamination reaction is important to both fundamental organic chemistry and biochemistry. Traditional chemical methods of deamination rely on the use of aryldiazonium salts under harsh acidic conditions, which limits the application scope for most biological substrates. Here we present an N-nitrosation strategy for deamination under mild conditions that DNA and RNA biological macromolecules can tolerate. Cooperative catalysis combining a carbonyl organocatalyst with a Lewis acid catalyst facilitates the formation of a carbon–nitro intermediate from a primary amine, which, on rearrangement into *N*-nitrosamine, leads to the selective deamination of unsubstituted canonical DNA/RNA bases under mild conditions. We used this approach to deaminate adenine into hypoxanthine, read as guanine by reverse transcriptases or DNA polymerases, while *N*^6^-methyladenosine sites resist deamination and remain identified as adenine. This reactivity enables a chemically mild, low-input detection method for sequencing of adenosine methylation at base resolution, named chemical cooperative catalysis-assisted *N*^6^-methyladenosine sequencing.

Chemical modifications in RNA and DNA play crucial roles in a wide range of biological processes, including transcription regulation, RNA degradation, protein translation and immune modulation^1–6^. These modifications have been quantitatively mapped at single-base resolution by new sequencing methods^7–14^, with nucleobase deamination being a highly effective strategy for the selective mapping of 5-methylcytosine in DNA^9,10^ and *N*^6^-methyladenosine (m^6^A) in RNA^12,13^.

In nitrite ion-based RNA sequencing methods^13,15–18^, adenosine (A) or cytidine (C) is deaminated through stepwise nitrosation and diazotization under acidic conditions (Fig. 1a). NO-seq and NT-seq are simple, cost-effective chemical methods, but their low conversion efficiencies and harsh acid treatment can compromise detection accuracy and RNA integrity through degradation^15,17^. Recently, Wang and co-workers reported the chemical-based approach of GLORI for m^6^A sequencing, which uses a glyoxal protecting group on guanosine (G) to substantially enhance deamination efficiency and selectivity for A in mammalian transcriptomes^13,18^. Despite being a major step forward, this method is still limited by RNA degradation under relatively harsh reaction conditions and reverse transcription stopping caused by incomplete glyoxal deprotection, necessitating a high input of RNA.

We sought to overcome the limitations of all previous nitrite ion-mediated nucleobase deamination approaches by designing a mild chemical deamination reaction that operates close to neutral pH, notably preserving RNA and improving the signal-to-noise ratio. While inspired by the evolved TadA-assisted *N*^6^-methyladenosine sequencing (eTAM-seq)^12^, GLORI^13^ and the chemoselective deamination of unmethylated adenines in general^15–17^, we were also intrigued by a report from Keeper and Roller in 1973 on the carbonyl-catalysed N-nitrosation ofsecondary amines using nitrite ions under neutral and basic media^19^. Adopting their strategy, we additionally employed a Lewis acid to facilitate N-nitrosation of the initial primary amine (Fig. 1b). We report here the development of a chemical sequencing method that mediates selective A-to-inosine (I) deamination in RNA to map m^6^A at base resolution under mild conditions (Fig. 1c). As the deamination is facilitated by cooperative catalysis using a carbonyl and Lewis acid as catalysts, we termed it chemical cooperative catalysis-assisted m^6^A sequencing (CAM-seq). CAM-seq promises a sensitive and robust mapping ofm^6^A with extensive coverage by avoiding harsh acidic treatment of RNA, allowing for more comprehensive quantification of m^6^A sites using a low input of sample.

## Results

### General deamination of substrates under mild conditions

When applying the conditions of carbonyl-catalysed N-nitrosation to nucleobase deamination, we envisioned that a Lewis acid catalyst could enhance the reactivity between the carbonyl organocatalyst and the heteroaromatic amine of nucleobases. Through preliminary deamination screenings of various Lewis acids and carbonyl compounds using cytidine as the substrate, we identified the optimal reaction conditions for the deamination process (Supplementary Tables 1–3.

**Fig. 2|:**
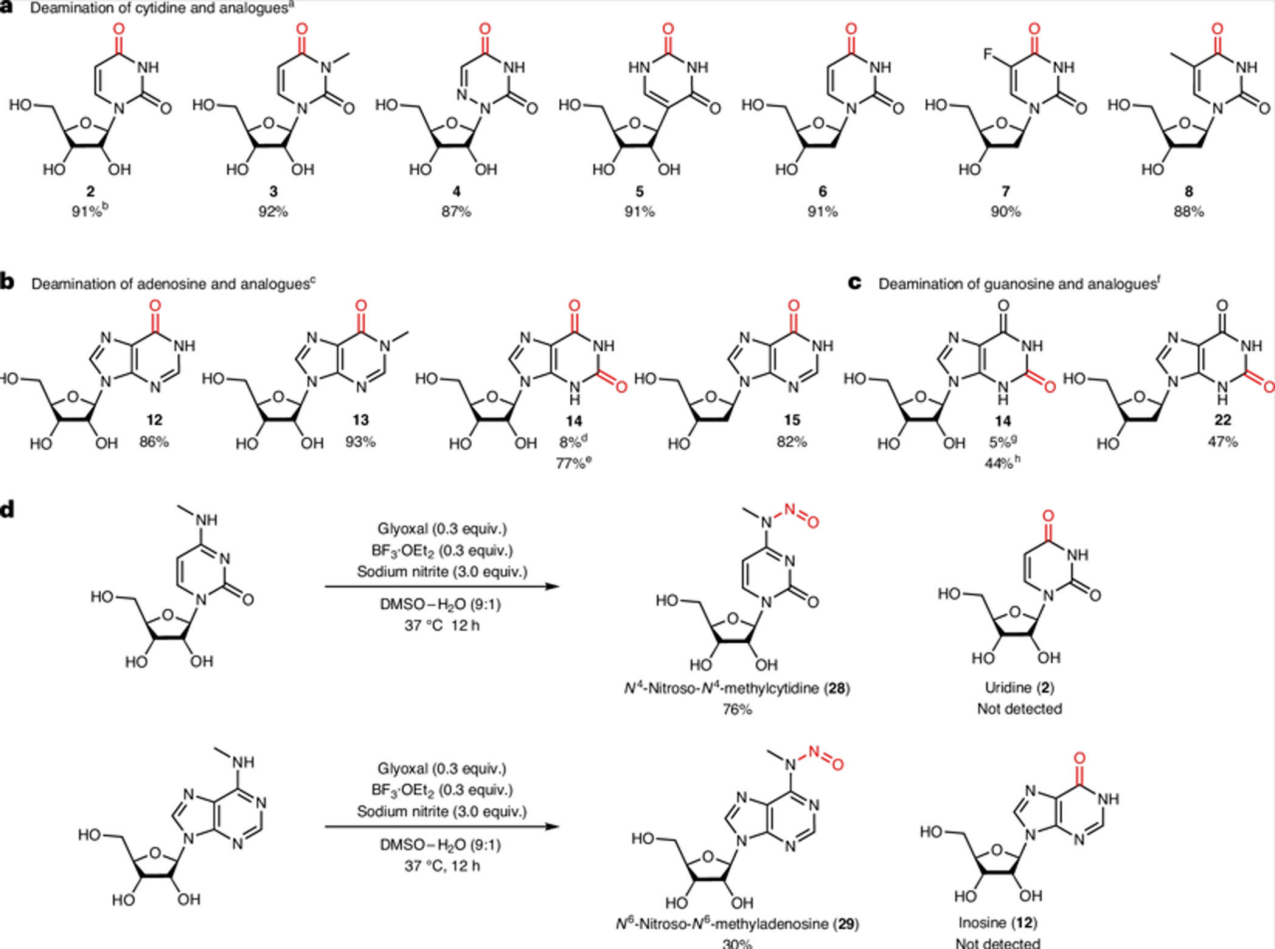
Deamination of nucleobases catalysed by a organocatalyst combined with a Lewis acid. **a**–**c**, Substrate scope of the deamination reaction with cytidine (**a**), adenosine (**b**) and guanosine (**c**) and their analogues (products and yields are shown). ^a^Deamination conditions A for cytidine and analogues: glyoxal (0.3 equiv.), BF_3_·OEt_2_ (0.3 equiv.), nitrite ion (3.0 equiv.) in dimethylsulfoxide (DMSO)–H_2_O (9:1) at 37 °C for 12 h. ^b^Isolated yield. ^c^Deamination conditions B for adenosine and analogues: glyoxal (0.3 equiv.), BF_3_·OEt_2_ (0.3 equiv.), nitrite ion (3.0 equiv.) in DMSO–H_2_O (9:1) at 37 °C for 12 h. ^d^2-Aminoadenosine was used as the starting material. ^e^Isoguanosine was used as the starting material. ^f^Deamination conditions C for guanosine and analogues: furfural (1.0 equiv.), BF_3_·OEt_2_ (1.0 equiv.), nitrite ion (3.0 equiv.) in DMSO–H_2_O (9:1) at 50 °C for 12 h. ^g^Guanosine deamination using conditions A. ^h^Guanosine deamination using conditions C. **d**, Reactions of methylated nucleosides under the optimized catalytic conditions. See Supplementary Materials for details.

On assessing the deamination compatibility of A, C and G nucleobase analogues with our selected co-catalyst conditions, we found that glyoxal with boron trifluoride diethyl etherate (BF_3_·OEt_2_) facilitated the highly efficient deamination of a broad range of nucleobase substrates (Fig. 2a–c and Supplementary Fig. 2). Switching from a dicarbonyl organocatalyst to furfural, a monocarbonyl organocatalyst, substantially increased the yield of guanosine analogue deamination. We hypothesize that this occurs because guanosine-like products at position 6 can cyclize with glyoxal, forming a guanosine–glyoxal adduct(Supplementary Figs. 1a,b)^20^.

Reactions with m^6^A and *N*^4^-methylcytidine (m^4^C) were shown to stop at the nitrosylated intermediate stage, with no detection of deaminated inosine or uracil products (Fig. 2d), consistent with previous experiments that used nitrite to drive nucleobase deamination^17^; a highly efficient and selective conversion of A or C but not m^6^A or m^4^C under mild conditions would present a robust method to map these methylated bases. We noted that the rate of conversion of m^6^A to its *N*-nitroso intermediate was lower than that of A, which contradicts previous observations^16^ and suggests a different mechanism behind the N-nitrosation step.

### Elucidation of the reaction mechanism

**Fig. 3|:**
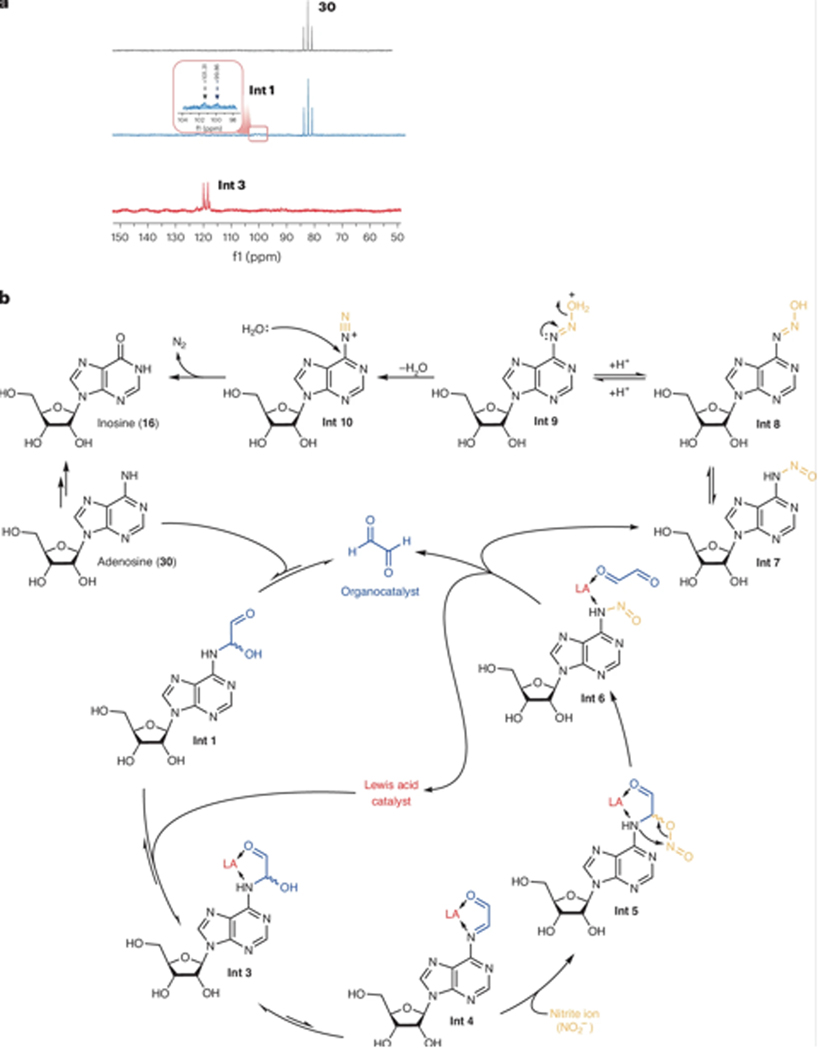
Mechanistic investigations and catalytic cycle. **a**, One-dimensional ^15^N NMR spectra of the deamination of [^15^N^6^]adenosine. ^15^N NMR spectra of [^15^N^6^] adenosine (top), the condensation product formed between [^15^N^6^]adenosine and the organocatalyst (middle) and the condensation product formed between [^15^N^6^]adenosine and the organocatalyst under Lewis acid catalysis (bottom). Inset: expansion of the ^15^N NMR spectrum around 100 ppm for **Int 1**. **b**, Proposed catalytic cycle for the deamination reaction.

We first followed the reaction by ^15^N nuclear magnetic resonance (NMR) spectroscopy, which revealed very poor condensation of [^15^*N*^6^]adenosine with glyoxal in the absence of the Lewis acid even after 2 h of heating at 50 °C (Fig. 3a, middle). Addition of BF_3_·OEt_2_ rapidly catalysed the conversion of ^15^N-labelled adenosine to the intermediate **Int 3** within half an hour (Fig. 3a, bottom).

Two-dimensional ^1^H–^15^N HMBC spectroscopy was used to monitor the Lewis acid-catalysed condensation with higher ^15^N sensitivity (Supplementary Figs. 4a–c)^21,22^. This technique was also used to monitor the reaction steps from the imine intermediate (Supplementary Fig. 5a). In the presence of Na^15^NO_2_, the [^15^N]imine signal disappeared, indicating its transformation to an aliphatic nitro-substituted intermediate and subsequent rearrangement to the N-nitrosation adduct (Supplementary Fig. 5b). When unlabelled adenosine was used, a clear, single signal from ^15^NO_2_ was observed (Supplementary Figs. 1c and 5c). These results confirm the pathway through the imine intermediate.

Building on these observations and previous studies of carbonyl-catalysed N-nitrosation, we proposed the reaction pathway depicted in Fig. 3b. Initially, the carbonyl catalyst and starting substrate condense to form a hemiaminal (**Int 1**), a process accelerated by the Lewis acid. Following dehydration to the imine intermediate **Int 4**, the nitrite ion interacts with the imine’s lowest unoccupied molecular orbital to form the C–O–N–O intermediate **Int 5**. This intermediate rearranges to form an N–NO bond, culminating in the formation of nitrosamine **Int 7** and the release of the organocatalyst and Lewis acid. ^11^B NMR experiments^23^ suggested that the Lewis acid could interact with both the carbonyl co-catalyst and the imine intermediate (Supplementary Fig. 3), as shown in **Int 3**–**Int 5**. Inosine (**16**) emerges after diazotization and deamination under slightly acidic conditions.

### Selective conditions for adenine deamination in DNA and RNA

Our group has previously developed various enzyme-assisted methods for mapping m^6^A modifications at single-base resolution, including selective allyl chemical labelling and sequencing (SAC-seq)^11,24^ and eTAM-seq^12^. However, we recognized the potential of a highly efficient, selective chemical deamination reaction as a cost-efficient alternative for m^6^A mapping, such as CAM-seq, involving carbonyl and Lewis acid co-catalysts under mild conditions. To make CAM-seq specific to m^6^A, we further explored the conditions to optimize its selectivity for adenine over cytosine and guanine in oligonucleotides, as well as the rate of deamination.

**Fig. 4|:**
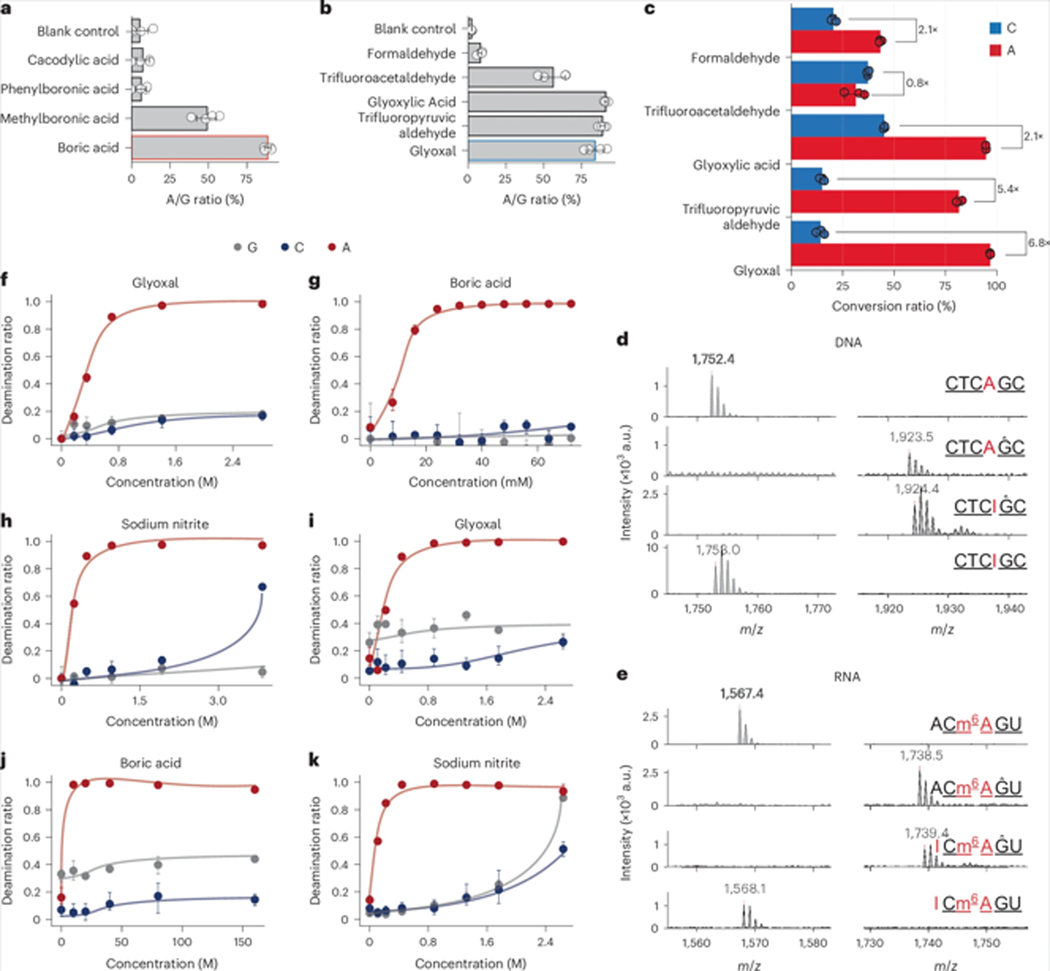
Optimization of deamination in oligonucleotides. **a,b**, The mutation ratio at adenine sites in an oligonucleotide for various Lewis acid catalysts (**a**) and carbonyl organocatalysts (**b**). The boric acid (red outline) and glyoxal (blue outline) catalysts were selected for further optimization. The error bars represent the 95% confidence interval (*n* = 4 technical replicates). **c**, Comparison of the catalytic efficiency of the deamination of A and C with various carbonyl organocatalysts. Dicarbonyl catalysts led to higher conversions on A sites than on C sites. The error bars represent the 95% confidence interval (*n* = 3 technical replicates), and the relative fold change of A conversion to C conversion is labeled. **d**, A 6-base DNA oligonucleotide CTCAGC under the optimized conditions shows high reactivity in both G protection and A deamination. **e**, A 5-base m^6^A RNA oligonucleotide probe (ACm^6^AGU) was used to demonstrate the selectivity between A and m^6^A under conditions similar to those used in **d**. Protected G sites are indicated by ^ and deaminated A sites are underlined. a.u., arbitrary units. **f**–**h**, Optimization of glyoxal (**f**), boric acid (**g**) and nitrite (**h**) concentrations for the deamination reaction of a DNA oligonucleotide, monitored via nucleotide composition analysis using LC–MS/MS (*n* = 3 biological replicates). The data are presented as the mean with 95% confidence intervals. Increased concentrations of glyoxal and boric acid did not affect the selectivity between A and G/C; however, higher nitrite concentrations substantially increased the conversion of C. **i**–**k**, Optimization of glyoxal (**i**), boric acid (**j**) and nitrite (**k**) concentrations for the deamination reaction of an RNA oligomer, monitored via nucleotide composition analysis using LC–MS/MS (*n* = 3 biological replicates). The data are presented as the mean with 95% confidence intervals. Increased concentrations of glyoxal and boric acid did not affect the selectivity between A and G/C; however, higher nitrite concentrations substantially increased the conversion of C.

We tested different Lewis acids by measuring the mutation ratio at adenine sites in an oligonucleotide. In contrast to the general deamination conditions, we found that boric acid exhibited the highest efficiency in enhancing reactivity (Fig. 4a and Supplementary Fig. 6a). With boric acid, dicarbonyl compounds achieved more efficient deamination than monocarbonyl compounds (Fig. 4b and Supplementary Fig. 6b). Using a liquid chromatography–tandem mass spectrometry (LC–MS/MS) assay, both trifluoropyruvic aldehyde and glyoxal demonstrated selectivity for adenine over cytosine (Fig. 4c and Supplementary Fig. 6c), but due to the notable DNA/RNA degradation caused by trifluoropyruvic aldehyde, glyoxal was selected for further study. This preference was similarly observed in RNA oligonucleotides (Supplementary Fig. 7a), confirming that dicarbonyl compounds are more reactive than monocarbonyl compounds.

We further evaluated these reactions using matrix-assisted laser desorption ionization time of flight mass spectrometry (MALDI–TOFMS)^25^. Complete deamination of A sites to inosines was observed in both DNA and RNA, whereas m^6^A sites remained unchanged (Fig. 4d,e). After optimizing the deamination conditions (Fig. 4f–k), glyoxal remained the most selective organocatalyst, as confirmed by the deep sequencing of a 35-base RNA oligonucleotide (Supplementary Fig. 7a).

Our optimized catalyst combination proved very robust across various temperatures and pH values, maintaining complete deamination at A sites with slightly reduced deamination at C and G sites as the temperature or pH was decreased (Supplementary Fig. 8a,b). These optimized conditions offer a deamination reaction under nearly neutral conditions, minimizing the degradation of biomacromolecules. Ammonium nitrites showed less deamination at C sites than sodium nitrite (Supplementary Fig. 8c), indicating the potential for even more selective conditions for adenosine deamination in RNA, although further optimization is required.

### Optimized conditions for m^6^A mapping (CAM-seq)

**Fig. 5|:**
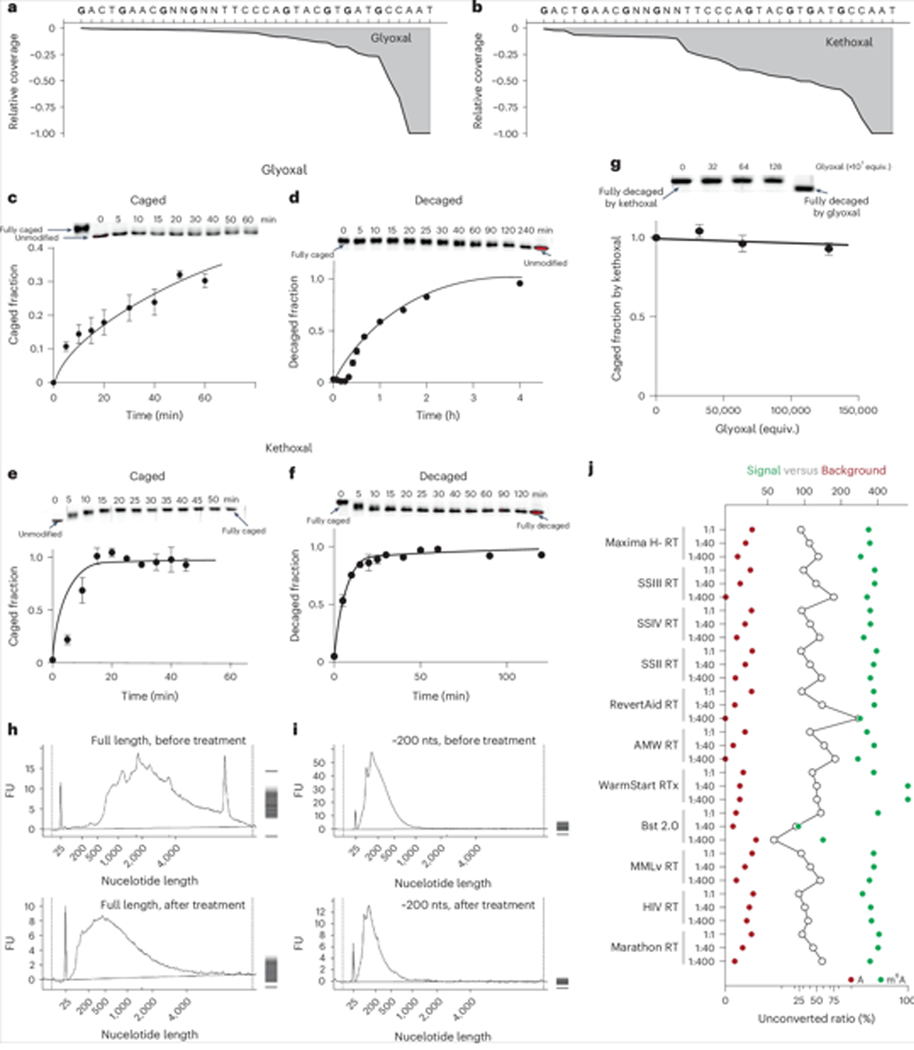
Optimization of the CAM-seq protocol. **a**, RT stopping was tested on an RNA oligonucleotide probe with G sites protected by glyoxal. The RT stop ratio reached approximately 75% at the first glyoxal-protected G site. **b**, A kethoxal-protected RNA oligonucleotide probe was used to demonstrate the RT stop ratio under conditions similar to those in **a**. The RT stop ratio reached only 40% at the first kethoxal-protected G site. **c**–**f**, Comparison of the caging (**c**,**e**) and decaging (**d**,**f**) kinetics of a DNA oligonucleotide using glyoxal (**c**,**d**) and kethoxal (**e**,**f**) under neutral conditions. The oligonucleotide was separated using PAGE gel, and the caged/decaged fraction was estimated from intensity. Data are presented as the mean with 95% confidence interval. **g**, Competition for the kethoxal fully caged DNA oligonucleotide by glyoxal under neutral conditions. The oligonucleotide was separated using PAGE gel, and the caged/decaged fraction was estimated from intensity. Data are presented as the mean with 95% confidence interval. **h**,**i**, RNA degradation assay using both full-length RNA (**h**) and 200-nucleotide fragmented RNA (**i**). The nucleotide length (nt) is shown on x-axis, and the RNA concentration measured by fluorescence units (FU) is shown on the y-axis. The gel image from the fragment analyzer is shown on the right. Following chemical treatment, full-length mRNA shows partial degradation to a 500-nt fragment, while fragmented RNA does not show notable degradation. **j**, Optimization of the reverse transcriptase condition was achieved through spike-in oligonucleotide sequencing data. Eleven reverse transcriptases and three dTTP/dCTP ratios (1:1, 1:40 and 1:400) were assayed. A stand-out condition (RevertAid RT at a 1:40 ratio) exhibited a background noise level below 0.36%. Open circles represent the signal-to-background ratio.

We noticed that, despite the effectiveness of glyoxal as an organocatalyst, its tendency to form adducts with guanine, followed by incomplete deprotection, could cause reverse transcriptase to stop during reverse transcription (known as RT stops). This is a limitation faced by GLORI in which glyoxal is used as a protecting group for G to minimize G-to-X conversions and ensure a selective deamination of adenosine. Therefore, we decided to use kethoxal as a protecting moiety due to its highly selective yet reversible reaction with G^25,26^. Glyoxal-caged RNA oligonucleotides showed a higher rate of RT stops, with 75% RT stops at the first caged G compared with 40% for the first kethoxal-caged G in RNA oligonucleotides (Fig. 5a,b). Kinetic studies of the DNA oligonucleotide template revealed that kethoxal displayed a caging and decaging rate 32 times greater than that of glyoxal under neutral conditions (Fig. 5c–f). Stability of the kethoxal protecting group could be enhanced under slightly acidic pHs, while a slightly basic pH facilitated easier removal (Supplementary Figs. 9j–l). Most importantly, glyoxal showed no ability to displace kethoxal from the fully caged kethoxal–guanine complex in competitive assays (Fig. 5g), which reinforced our preference for kethoxal as the protecting group for guanine during CAM-seq.

The acidic conditions used in previous nitrite deamination methods can cause notable RNA degradation^13,15–17^. We found that operating CAM-seq at a neutral to slightly acidic pH substantially reduced RNA degradation. While the optimized conditions caused a small amount of degradation when using long, full-length messenger RNA (Fig. 5h), negligible degradation was observed using 200-nucleotide mRNA fragments as input RNA (Fig. 5i).

Despite CAM-seq reagents achieving nearly 100% conversion efficiency in LC–MS/MS assays, ~1% of A sites remained unchanged in sequencing data. We reasoned that RT reactions may also yield false positives. Partial pairing of inosine with 2′-deoxythymidine (dT) could lead to the misinterpretion of m^6^A as unmodified A, and RT stops at inosine sites could create bias in quantification^27,28^. To address potential RT-induced artefacts, we examined 11 different reverse transcriptases and 3 different dNTP ratios, that is, 2′-deoxythymidine triphosphate (dTTP) to 2′-deoxycytidine triphosphate (dCTP) ratios of 1:1, 1:40 and 1:400. CAM-seq processing of 10 ng polyA-tailed RNA from HEK293T cells spiked in with m^6^A-modified RNA oligonucleotide (Supplementary Note I) revealed varying propensities of reverse transcriptases to incorporate dT during RT reactions (Fig. 5j and Supplementary Figs. 10–12). Considering that the conversion ratio and RT efficiency could be affected by local sequence context, representing the overall performance with a single oligonucleotide sequence may be overly simplistic. We therefore calculated the unconverted ratio of all A sites in the human transcriptome for each condition to better estimate the signal-to-noise ratio (Supplementary Fig. 12). GAC motifs, which are highly enriched for m^6^A modifications, served as a positive control, while UAG motifs, less enriched, were used as a negative control. Comparing signal-to-noise ratios led us to select condition RevertAid RT with a 1:40 dTTP/dCTP ratio for its ~fourfold decrease in background noise, with the signal from GAC motifs reduced by less than one-fifth. Under these optimized RT conditions, the background noise from unmodified A sites was less than 0.5% (Supplementary Fig. 12).

### Comprehensive m^6^A mapping in human transcriptome

The progression of m^6^A sequencing technologies has evolved over the past 10 years with notable advances in resolution^29–31^ and quantification^30,31^. The latest techniques^11–13^ combine single-base accuracy with quantification but still face some challenges in bias. The lessened effectiveness of SAC-seq for AAC motifs^11^ can potentially produce false negatives, while eTAM-seq^12^ and GLORI-seq^13^ encounter a background noise of 1–2% that escalates to >10% in highly structured regions such as ribosomal RNA (Supplementary Fig. 13). To attain a thorough grasp of the m^6^A landscape across the transcriptome, a method with much lower background noise devoid of false negatives is essential. Our optimized CAM-seq protocol selectively converts adenosine sites into inosine; after RT, inosine is paired with dCTP and read as an A-to-G mutation, while m^6^A reads as A after PCR (Supplementary Fig. 14). The optimized protocol also dramatically reduces RNA degradation and background noise, enabling its application to low input samples. Thus, CAM-seq was used to map m^6^A throughout the transcriptome in both human and plant samples using 10 ng polyA-enriched RNA.

**Fig. 6|:**
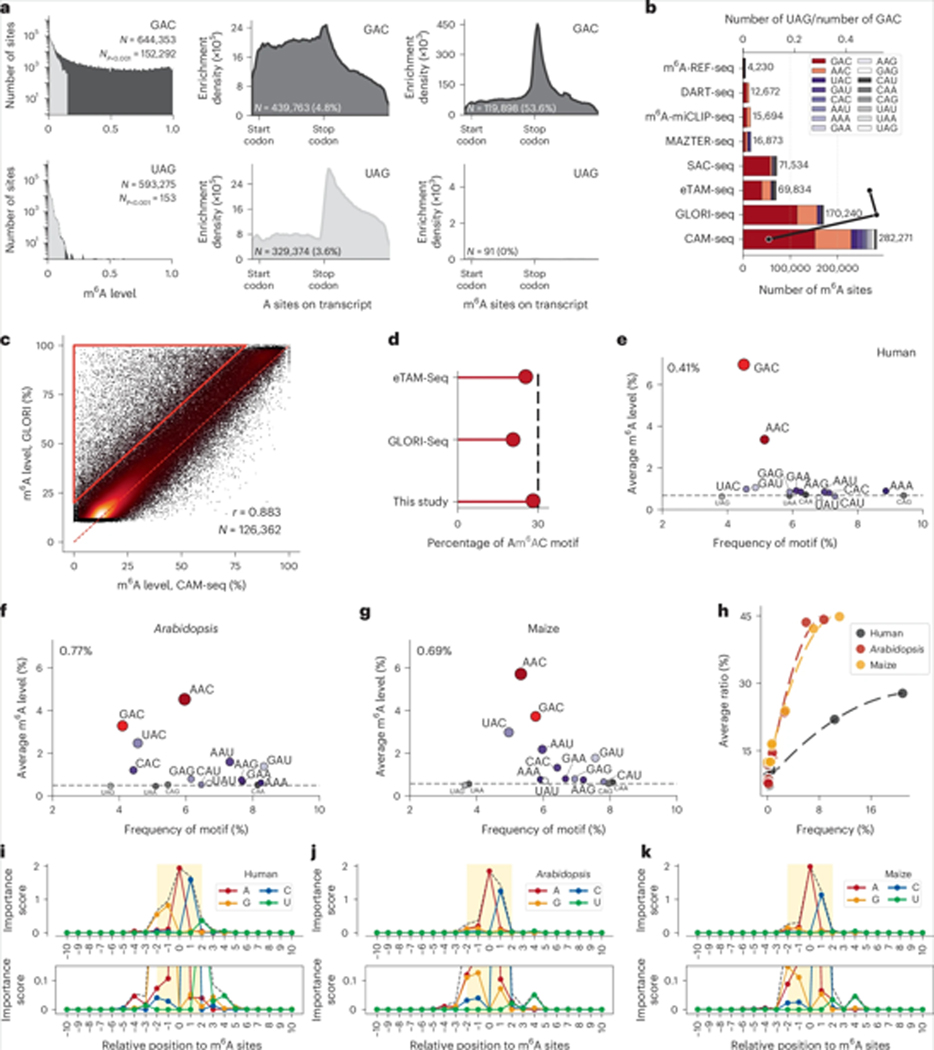
Accurate quantification of m^6^A modification reveals m^6^A deposition at the motif level. **a**, Distribution of m^6^A modification levels for all A sites (coverage > 20), focusing on GAC and UAG motifs as examples. Left: sites with *P* < 0.001 are marked in black and non-significant sites are marked in grey. The total number of A sites (*N*) and filtered m^6^A sites (*N*_*P* < 0.001_) are indicated. **b**, Comparison of detected m^6^A sites and estimated background noise for multiple methods. The bars show the total number of m^6^A sites, with GAC and AAC motifs in shades of red and other motifs in shades of purple. The black line indicates the UAG/GAC ratio, used as an estimate of the upper bound for false positives. **c**, Correlation between CAM-seq and GLORI-seq m^6^A levels for 126,362 overlapping sites (*r* = 0.883). Red dashed line mark the equal levels. The area marked by the red triangle highlights sites where GLORI-seq overestimates m^6^A levels. **d**, Fraction of AAC motifs among all m^6^A sites observed in three deamination-based methods, calculated as the sum of (m^6^A ratio × coverage) for AAC sites over the sum of (m^6^A ratio × coverage) for all sites. **e**, Average methylation level versus relative motif frequency across all A sites (no filter) for human samples. GAC and AAC motifs (red) stand out above the background. The overall m^6^A level is indicated as 0.41%. **f**,**g**, Average methylation level versus relative motif frequency across all A sites for *Arabidopsis* (**f**) and maize (**g**). The overall m^6^A levels are indicated as 0.77% and 0.69%, respectively. **e**–**g**, the dotted-dashed lines indicate the background noise, defined by the rarely methylated motifs (UAG/UAA/CAG/CAA). **h**, Relationship between motif frequency and average methylation level for the 16 three-letter motifs passing filters in human, *Arabidopsis* and maize. **i**–**k**, Nucleotide importance scores at each position relative to m^6^A sites in human (**i**), *Arabidopsis* (**j**) and maize (**k**). The dashed black lines show the total importance at each position.

Using the optimized RT conditions (RevertAid RT, 1:40 dTTP/dCTP ratio) for the human HEK293T cell line, we identified 282,281 highly confident m^6^A sites (*P* < 0.001). Of these, 235,173 and 180,056 sites exhibited modification ratios greater than 5% and 10%, respectively (Supplementary Fig. 15a,b). The GAC motif emerged as the most enriched, with 152,292 sites (54% of confident sites) within the HEK293T transcriptome (Fig. 6a), followed by the AAC motif (underlined A indicates modified site), which accounted for 76,475 sites (27%). Notably, YAC (CAC/UAC) and RAU (GAU/AAU) motifs, which differ by only one nucleotide from the RAC motif, also showed a degree of m^6^A modification. Other motifs, such as UAA/UAG/UAU, showed relatively rare m^6^A modifications. For example, of all the detected m^6^A sites, only 0.054% were in UAG motifs. m^6^A-enriched motifs such as GAC, but not m^6^A-rare motifs such as UAG, exhibited a distinct enrichment signal at the 3′-UTR region near the stop codon in the human transcriptome (Fig. 6a and Supplementary Fig. 15c,d), further confirming that CAM-seq provides high-quality m^6^A mapping across the entire transcriptome. We also calculated the ratio of UAG to GAC as a measure of the signal-to-noise level and for comparison with other methods.

This study presents the most comprehensive dataset for m^6^A detection in the human transcriptome so far (Fig. 6b). The signal-to-noise level of CAM-seq, calculated on the basis of the ratio of m^6^A on the UAG motif to m^6^A on the GAC motif, is approximately 0.05%, which is ten times lower than published results obtained in the same HEK293T cell line using the other two deamination-based methods, eTAM-seq and GLORI-seq. Enzyme-based methods, such as MAZTER-seq, m^6^A-miCLIP-seq, DART-seq and SAC-seq, exhibit motif bias, failing to detect all m^6^A sites. These results align with the observation in previous studies that a notable proportion of sites show an overestimation of m^6^A levels (Fig. 6c), probably due to incomplete deamination. Before the development of m^6^A sequencing methods, liquid chromatography–mass spectrometry (LC–MS) determined that approximately 30% of m^6^A sites were located in AAC motifs^32^; however, it has been challenging to validate this observation using high-throughput sequencing data due to the potential for false positives and false negatives. The ability of CAM-seq to uncover low-ratio motifs and eliminate false positives provides a more accurate alignment with LC–MS observations. By leveraging the CAM-seq data, we calculated the overall motif composition by sum of the (m^6^A ratio × site coverage of m^6^A sites on the AAC motif) over the sum of the (m^6^A ratio × site coverage of all m^6^A sites), deriving values of 25.4%, 20.5% and 28.1% for eTAM-seq, GLORI-seq and CAM-seq, respectively (Fig. 6d). These results suggest that CAM-seq can accurately uncover the absolute modification profile of a given sample, more accurately aligning with LC–MS observations and providing a reliable representation of the m^6^A landscape.

### Cross-species study uncovers motif-dependent m^6^A variation

Motivated by this observation, we investigated whether this m^6^A modification profile at the motif level is consistent across animal and plant species. *Arabidopsis* and maize RNA samples were extracted and sequencing libraries were prepared using the optimized RT condition (RevertAid RT, 1:40 dTTP/dCTP ratio). With a stringent cut-off *P* value of <0.001, we detected 111,121 and 141,990 m^6^A sites in *Arabidopsis* (Supplementary Fig. 16a,b) and maize (Supplementary Fig. 17a,b) samples, respectively. Similar to the human transcriptome, RAC motifs were the motifs most prevalently enriched with m^6^A in these plants. However, RAC motifs constitute only 40% of all m^6^A sites in plants, compared with about 85% in human samples. Interestingly, the AAC motif is the most abundant in *Arabidopsis* and maize, in contrast to the GAC motif in humans. Motifs that differ by one nucleotide, such as YAC and RAU, account for ~50% of the sites in plants, whereas they represent only ~10% in humans. This variation in m^6^A motif enrichment between plants and humans raises questions about whether m^6^A regulation is less stringent in the plant kingdom. On further investigation, the frequency of one m^6^A-rare motif (UAG) was found to be 0.043% and 0.051% in *Arabidopsis* and maize, respectively. These observations suggest that m^6^A writers in plants may exhibit a wider motif spectrum, but are still tightly regulated, similarly to humans (Fig. 6e–g).

The frequencies of m^6^A sites within all three-letter motifs were analysed against their average modification level, revealing an overall positive correlation. This means that motifs with higher abundances tend to have higher m^6^A modification levels (Fig. 6h). Interestingly, the plant samples exhibited a nearly identical trend, while the data for humans showed a smaller slope. This indicates that the m^6^A modification in human samples is more evenly distributed than in plants, where the m^6^A modification is more concentrated at specific sites. The analysis of five-letter motifs showed similar trends (Supplementary Figs. 15b–17b). For example, GGACT and CGACT both belong to the GAC motif, but 34,261 versus 30 modified sites were detected, with modification levels of 38% versus 7%, respectively. This suggests that the flanking bases at positions −2 and +2, in addition to those at positions −1 and +1, play important roles in regulating m^6^A deposition in humans. To unravel the rules governing m^6^A deposition at the motif level, we developed an importance score for each nucleotide at positions around the m^6^A site. The importance score represents the relative contribution of each position within the motifs, ranging from nucleotides −10 to +10 flanking the m^6^A site, based on the level of m^6^A methylation of each motif through the calculation of entropy (see Methods). The modification level is considered in this calculation rather than just the relative motif frequency. Consistent with previous studies, the modification level in the human transcriptome is significantly influenced by the cytosine (C) base at position +1 (Fig. 6i). In addition, the guanine (G) base at positions −2 and −1 and uracil (U) at position +2 notably affect the modification level of m^6^A motifs. Surprisingly, positions −4, −3, +3 and +4 also contribute to the m^6^A level: adenine (A) at position −4 and uracil (U) at position +4 are associated with a higher m^6^A modification level, whereas positions −3 and +3 show no clear nucleotide preference. Distal sites more than four nucleotides away do not significantly impact the m^6^A level.

Similar analyses of *Arabidopsis* (Fig. 6j) and maize (Fig. 6k) samples revealed that, despite the evolutionary diversity of these two plants, the rules for m^6^A deposition are relatively conserved. In contrast to human samples, only cytosine (C) at position +1 showed a significant importance score in these plants. Adenine (A) at position −1 plays a more crucial role than guanine (G) at the same position, while adenine (A) and guanine (G) at position −2 demonstrate comparable levels of importance. Interestingly, although position +2 contributes to m^6^A deposition, its total importance score is as low as that of position +4. Moreover, positions +2 and +3 also do not exhibit a preference for any specific nucleotide, although uracil (U) at position +4 contributes more to the m^6^A level, in alignment with the observations in human samples.

### Saturated profiling links transcription rate to m^6^A level

**Fig. 7|:**
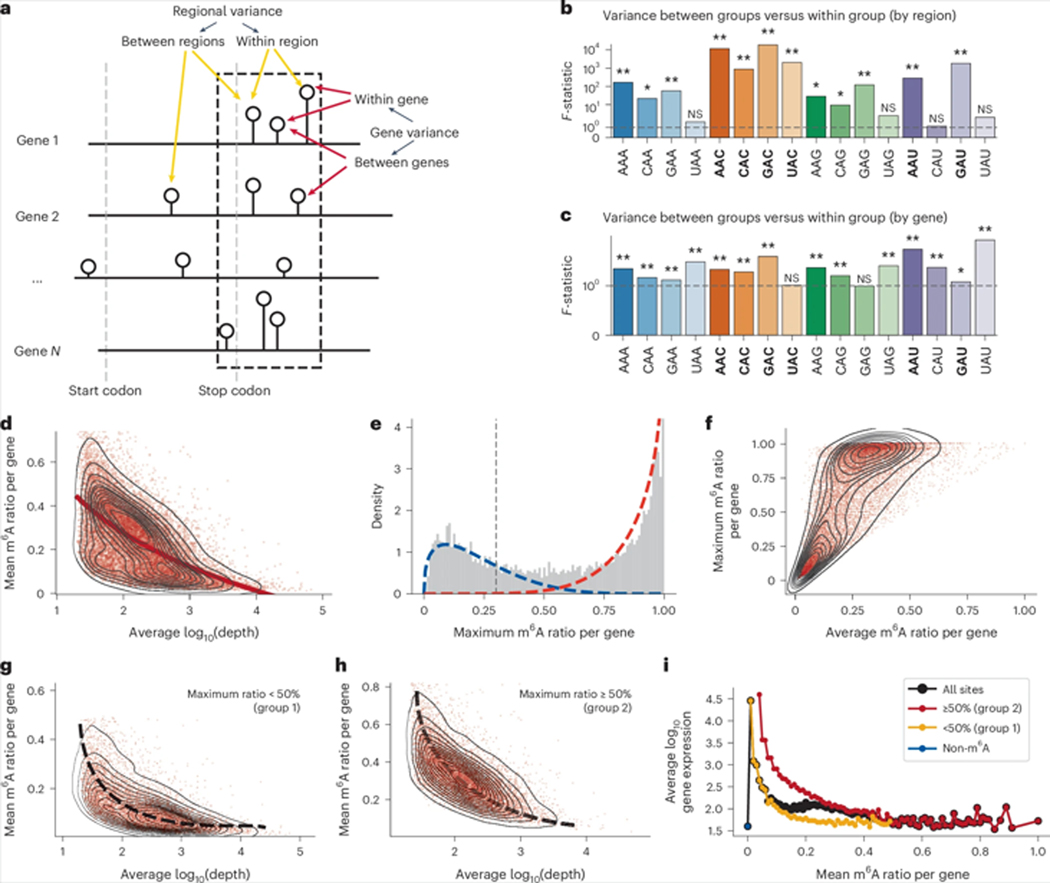
Factors affecting m^6^A variation. **a**, Illustration showing how the regional and gene distribution of A sites may affect the variance of the m^6^A modification level in genes. **b**, The region from −20 to +180 nucleotides near the stop codon is identified as an m^6^A deposition hotspot based on metagene analysis. All A sites are divided into two categories: those within this window and those outside. The variance of m^6^A sites between and within these regions was compared, followed by a one-way analysis of variance test. The *F*-statistic represents the ratio of the variance between groups to the variance within groups, with the *P* value indicating the significance of this comparison (**P < 10^−10^, *P < 10^−2^, NS, not significant). The dashed lines indicate an *F*-statistic of 1. Major m^6^A motifs in the human transcriptome are highlighted in bold. **c**, Corresponding data for the variance between genes and within a single gene. Only A sites in the −20 to +180 hotspot region were analysed as other regions were minimally modified (**P < 10^−10^, *P < 10^−2^, NS, not significant). **d**, Relationship between log_10_ gene expression and average m^6^A level, shown as a contour plot with a dotted trend line. **e**, Distribution of the maximum m^6^A modification level of all the genes. The fitting curve for low methylation and high methylation groups are marked by a blue and red dashed line, respectively. **f**, Correlation between the average and maximum m^6^A modification levels within genes. **g**,**h**, log_10_ gene expression level versus the average m^6^A level for lowly modified genes only (**g**) and highly modified genes only (**h**). **i**, Overall relationship between m^6^A level and gene expression for all genes (black), genes without m^6^A modification (blue), low-m^6^A-modified genes (yellow) and high-m^6^A-modified genes (red).

Despite extensive research into the role of motif patterns in regulating m^6^A levels, the variability in the degree of methylation among identical motifs remains unexplained. Over the past decade, several studies have identified factors that regulate the level of m^6^A site modification, such as the proximity to the stop codon, internal exon length, last exon localization and the presence of the exon junction complex. We refer to these influencing factors as regional variance, which can be demonstrated through global-scale statistical analyses, even in noisy and sparse datasets (Fig. 7a). For instance, by comparing the modification levels of sites within the −20 to +180 region near the stop codon with those outside this region, we observed that the regional distribution significantly impacts most A motifs (Fig. 7b).

Another variance exists at the gene level. Even when identical regions are considered, with all other features such as the distance to the stop codon being constant, notable differences in levels of m^6^A methylation are observed between genes (Fig. 7a). Previous challenges in studying this variance arose due to the absence of methods capable of generating a saturated map of m^6^A modifications, making the confirmation of m^6^A sites across genes uncertain. Using the saturated m^6^A dataset in this Article (Supplementary Figs. 18a,b), we can now accurately quantify the modification of all A sites with sufficient sequencing coverage. Our analysis revealed that gene-level factors significantly affect m^6^A variance (Fig. 7c), prompting an investigation into how genes influence m^6^A levels.

We observed a generally negative association between gene expression level and average m^6^A level (Fig. 7d). However, analysis of the density of the genes showed that the data points for the genes could be roughly separated into two clusters. This observation led us to speculate that the average m^6^A level of different genes might follow a bimodal distribution, but that was not the case (Supplementary Fig. 19c). We further reasoned that the level of modification of m^6^A could be influenced by motif pattern, regional distribution and the length of exons, and that variance of these factors might obscure comparison of m^6^A differences at the gene level. Consequently, the maximum modification level of a gene, indicative of its maximum capacity for m^6^A modification, might be a better parameter for distinguishing genes. When distinguishing genes by their maximum modification level, we found that all m^6^A-modified genes in the human transcriptome could be divided into two distinct groups (Fig. 7e,f): one with generally low modification across all m^6^A sites and the other with the potential for highly modified sites. Thus, we attempted to classify these genes into two groups based on whether the maximum modification level is below or above 50% (group 1 and group 2, respectively). Both groups showed a notable negative correlation between gene expression level and m^6^A level (Fig. 7g,h). We propose two possibilities to explain this observation: (1) genes with high m^6^A modification levels tend to be degraded, leading to a systematic drop in the expression level, and (2) genes with high expression levels show high transcription rates, and within cells, a higher transcription rate may contribute to a lower probability of methylation. Comparison of non-m^6^A-modified genes, group 1 genes and group 2 genes revealed that group 2 genes, with a higher capacity for m^6^A modification, showed higher expression levels than group 1, and group 1 genes showed higher expression levels than non-m^6^A genes (Fig. 7i). These observations suggest that m^6^A-mediated degradation may not be the primary factor in this trend, while the transcription rate might play an important role.

## Discussion

We have reported here a nucleoside deamination reaction involving cooperative catalysis with a carbonyl organocatalyst and Lewis acid. We have shown that this catalytic reaction proceeds through N-nitrosation, diazotization and denitrogenation. Our mechanistic studies revealed that boric acid acts as a Lewis acid during deamination, implying a role beyond that of a protective additive for guanine, as previously reported^13^. The harsh acidic reaction conditions of previous deamination procedures have limited their application in RNA modification sequencing^14,16^. We have shown here that the use of a boric acid buffer to adjust the pH to nearly neutral is crucial to maximize the conversion of adenine and minimize RNA degradation under the mild deamination conditions.

We selected kethoxal as the reactive and reversible protecting group for guanosine, avoiding harsh deprotection and reverse transcription stalling due to incomplete deprotection. Combined with optimal RT conditions, we have developed CAM-seq, a transcriptome-wide m^6^A sequencing method that has lower input RNA requirements, a shorter reaction time, streamlined procedure and reduced costs compared with other sequencing methods. CAM-seq can identify approximately 200,000 m^6^A sites using as little as 10 ng of input mRNA with background noise as low as 0.5%.

Saturated m^6^A profiling throughout the transcriptome using CAM-seq advances analyses from semi-quantitative to nearly quantitative, moving from limited profiling to comprehensive profiling. While previous methods such as meRIP-seq, m^6^A-REF-seq, m^6^A-SAC-seq, eTAM-seq and GLORI-seq provided valuable insights, they were limited to semi-quantitative analysis or limited profiling, although m^6^A-SAC-seq^11,24^ and related chemical approaches^14^ could directly read out m^6^A and retain sequence complexity. Our CAM-seq method, with its remarkably low background noise, facilitates systematic quantification of m^6^A sites in most regions within most transcripts, enabling unbiased comparisons at the gene level. Using this sequencing method, we systematically explored m^6^A variance at different layers: motif dependency, region variance and gene level. Consistent with previous studies, we found that m^6^A modifications are enriched at RAC motifs and near stop codons. Beyond this, we quantitatively compared the m^6^A distribution among different motifs in human and plant samples and found that YAC (CAC/UAC) and RAU (GAU/AAU) motifs, which differ by only one nucleotide from the RAC motif, also show enriched m^6^A modification, especially in plant samples. More importantly, our results suggest that the transcription rate is a potential determinant of m^6^A levels. Specifically, genes with high transcription rates tend to have lower m^6^A levels, possibly due to a limited availability of the m^6^A deposition complex or dynamic association of the methyltransferase complex with transcription machineries within the cell.

Overall, CAM-seq represents a important advance in RNA modification mapping, providing a robust, efficient and truly quantitative method for comprehensive m^6^A sequencing. Furthermore, it will enhance our understanding of m^6^A dynamics in various biological contexts with quantitative and saturated data.

### Online content

Any methods, additional references, Nature Portfolio reporting summaries, source data, extended data, supplementary information, acknowledgements, peer review information; details of author contributions and competing interests; and statements of data and code availability are available at https://doi.org/10.1038/s41557-025-01801-3.

## Methods

### General procedure for deamination

A 4-ml screw-cap vial was charged with the substrate nucleobase (0.1 mmol, 1.0 equiv.) and solvent mixture. The organocatalyst (0.03 mmol, 0.3 equiv.) and Lewis acid catalyst (0.03 mmol, 0.3 equiv.) were then added in one portion at room temperature, followed by sodium nitrite (3.0 equiv.). The reaction vial was sealed and incubated at 37 °C for 12 h. The crude reaction mixture was then concentrated under reduced pressure (in vacuo) and purified by flash column chromatography on silica gel to obtain the product.

### General procedure for MALDI–TOF MS analysis

A 1.0 μl aliquot of a 100 μM synthetic DNA oligonucleotide (5′-CTCAGC-3′) was mixed with 6.0 μl nuclease-free water. Next, 2.0 μl of 5× reaction buffer (0.5 M sodium cacodylate, 50 mM MgCl_2_, pH 7.0) and 1.0 μl of 1.0 M N_3_-kethoxal (dissolved in DMSO) were added, and the mixture was incubated at 37 °C for 10 min. A solution containing 20 μl of 8.8 M glyoxal and 5 μl of 10× PBS buffer was then added, followed by 15 μl H_3_BO_3_ and 10 μl nuclease-free water at ambient temperature. This reaction mixture was then incubated at 50 °C for 30 min. Subsequently, 40 μl of a deamination buffer (comprising 10 μl saturated sodium nitrite solution, 10 μl of 5× HEPES buffer (0.5 M, pH 6.0) and 20 μl nuclease-free water) was added and mixed thoroughve cycles, after which the reaction was held at 4 °C until the next step. The DNA was precipitated with ethanol, reconstituted in 5.0 μl nuclease-free water and used directly for MALDI–TOF MS analysis. For the MALDI–TOF MS analysis, a matrix solution was prepared by mixing 2′,4′,6′-trihydroxyacetophenone (10 mg ml^−1^ in 50% CH_3_CN–H_2_O) and ammonium citrate (50 mg ml^−1^ in H_2_O) in a ratio of 8:1 (v/v). A 1.0 μl aliquot of the purified reaction product was combined with 1.0 μl of the matrix, spotted onto the MALDI–TOF sample plate, allowed to dry and analysed using a Bruker Ultraflextreme MALDI–TOF/TOF mass spectrometer.

### General procedure for triple quad analysis

First, 1.0 μl of a 100 μM solution of synthetic 60-base DNA oligonucleotide was mixed with 6.0 μl nuclease-free water, 2.0 μl 5× reaction buffer (0.5 M sodium cacodylate, 50 mM MgCl_2_, pH 7.0) and 1.0 μl of 1.0 M N_3_-kethoxal (DMSO solution). The mixture was then incubated at 37 °C for 10 min. Next, 20 μl of 8.8 M glyoxal solution and 5 μl of 10× PBS were added to the reaction mixture, followed by 15 μl H_3_BO_3_ and 10 μl nuclease-free water under ambient conditions. The reaction mixture was then incubated at 50 °C for 30 min. Finally, 40 μl of a deamination buffer (comprising 10 μl saturated sodium nitrite solution, 10 μl of 5× HEPES buffer (0.5 M, pH 6.0) and 20 μl nuclease-free water) was added and the solution mixed well. The reaction mixture was heated at 37 °C for 5 min and then at 18 °C for 30 min for six cycles, and then held indefinitely at 4 °C until further purification. DNA was precipitated from the solution using ethanol and reconstituted in 50 μl nuclease-free water. Untreated deaminated oligonucleotides (50–150 ng) were diluted in 17 μl nuclease-free H_2_O and denatured at 95 °C for 5 min, then immediately chilled on ice for 2 min. Next, 1 μl nuclease P1 (1 U μl^−1^, Wako, 145–08221) and 2 μl of 100 mM NH_4_OAc solution were added to the reaction mixture and incubated at 42 °C for 2–4 h (or overnight). Then, 1 U of FastAP thermosensitive alkaline phosphatase and 2.7 μl of 10× FastAP buffer (Thermo Scientific, EF0651) were added, followed by incubation at 37 °C for 2–4 h (or overnight). Digested oligonucleotides were diluted to 60 μl with nuclease-free H_2_O and filtered through a 0.22 μm filter (Millipore, SLGVR04NL). Samples were analysed directly in a SCIEX Triple Quad 6500+ LC–MS/MS system. For each analysis, 10 μl of sample was injected and the nucleosides were separated by reversed-phase ultra-high pressure liquid chromatography on a C18 column (Agilent, 927,700–092), followed by detection by MS. Nucleosides were quantified using nucleoside precursor ion to base ion mass transitions of *m*/*z* 253.1 to 135.0 for dI and *m*/*z* 252.1 to 136.0 for dA. The nucleoside concentration was quantified using the calibration curves obtained from nucleoside standards measured under the same conditions. The final ratios of dI/dA and I/A were calculated by subtracting the background (mock control) generated by digestion enzymes.

### Cell culture

HEK293T cells were obtained from the American Type Culture Collection. The cells were maintained at 37 °C under 5% CO_2_ in a Heracell VIOS 160i incubator (Thermo Scientific). All cell lines were cultured in DMEM (GIBCO, 11995), supplemented with 10% (v/v) fetal bovine serum and 1% penicillin–streptomycin (GIBCO).

### Plant material collection

The Col-0 accession of *Arabidopsis thaliana* and maize inbred line B73 (*Zea mays*) were used in this Article. *Arabidopsis* plants were grown at 22 °C with 16 h of light per 24 h. The seedlings were collected after growing on 1/2 Murashige and Skoog medium plates for 7 days. Maize inbred line B73 seeds were germinated at 28 °C with 14 h of light per 24 h. The shoots of 7-day-old maize plants were collected. The collected tissues were flash-frozen in liquid nitrogen, ground using a mortar and pestle, and stored at −80 °C until further use.

### PolyA-tailed RNA isolation

Total RNA was extracted from cells or plant tissue using TRIzol reagent (Ambion by Life Technologies) and Direct-zol RNA miniprep kit (Zymo Research) following the manufacturer’s protocol. After RNA extraction, two rounds of poly(A) enrichment were conducted using DynaBeads mRNA direct purification kit (Thermo Fisher Scientific) following the manufacturer’s protocol with some modifications. Accordingly, 200 μl Dynabeads were washed with 200 μl lysis/binding buffer and then mixed with 100 μg total RNA in 300 μl lysis/binding buffer. The samples were then incubated on the roller mixer at room temperature for 20 min. The beads were washed twice with washing buffer A and once with washing buffer B in the kit. The beads were resuspended in 50 μl of 10 mM Tris–HCl (pH 7), incubated at 70 °C for 3 min for washing and then eluted with 10 μl buffer. The eluate was subjected to a second round of poly(A) enrichment following the same procedure.

### General procedure for library preparation

Wild-type HEK293T cells and plant tissues were collected in three biological replicates. PolyA+ RNA was extracted from total RNA and 10–20 ng of the RNA was fragmented using RNA fragmentation reagents (NEB, E6150S) at 94 °C for 2 min, followed by purification with the RNA Clean and Concentrator kit (Zymo Research). After chemical treatment, 3′-end repair was performed on both the ‘input’ and ‘treated’ samples using T4 polynucleotide kinase (PNK, NEB, M0201S). RNA was combined with 2 μl of 10× T4 PNK reaction buffer (NEB, B0201S) and 3 μl T4 PNK, diluted to a final volume of 20 μl and incubated at 37 °C for 60 min. The mixture was then purified using the RNA Clean and Concentrator kit (Zymo Research) and eluted with 10 μl RNase-free water. For 3′-adapter ligation, 10 μl of 3′-repaired RNA fragments were incubated with 1 μl of 20 μM RNA 3′-adapter (/5rApp/AGATCGGAAGAGCGTCGTG/3Bio/) at 70 °C for 2 min and immedi ately placed on ice. The reaction was supplemented with 2.5 μl of 10× T4 RNA ligase reaction buffer (NEB, M0373L), 7.5 μl poly(ethylene glycol) 8000 (PEG8000; 50%), 1 μl SUPERase·In RNase Inhibitor and 1 μl T4 RNA ligase 2 truncated KQ (NEB, M0373L). The mixture was incubated at 25 °C for 2 h, followed by incubation overnight at 16 °C for 12 h. The reaction mixture was diluted to a final volume of 47 μl with nuclease-free water and excess adapters were removed by adding 2 μl 5′-deadenylase (NEB, M0331S) and incubating at 30 °C for 30 min, followed by the addition of 1 μl RecJf (NEB, M0264L) for single-stranded DNA digestion at 37 °C for 30 min. The 3′-end-ligated RNA was purified using the RNA Clean and Concentrator kit (Zymo Research) and eluted with 10 μl RNase-free water. The ‘input’ and ‘treated’ samples were both mixed with 1 μl of 2.0 μM RT primer (5′-ACACGACGCTCTTCCGATCT-3′), heated at 65 °C for 2 min and immediately placed on ice. To this mixture, 1 μl of 5× RT buffer, 3.05 μl dNTP solution mix, 1 μl RNaseOUT recombinant ribonuclease inhibitor (Thermo Scientific, 10777019) and 1 μl RevertAid H Minus reverse transcriptase (Thermo Scientific, EP0452) were added. The mixture was thoroughly mixed and incubated at 42 °C for 1 h, followed by the addition of 1 μl RNase H (NEB, M0297L) and incubation at 37 °C for 20 min. The reaction mixture was then heated at 70 °C for 5 min and the resulting complementary DNA was purified using the DNA Clean and Concentrator kit (Zymo Research). The purified cDNA was mixed with 2.0 μl of 10 μM cDNA adapter (5′- /5Phos/NNNNNNNNNNAGATCGGAAGAGCACACGTCTG/3SpC3/−3′), heated at 70 °C for 2 min and immediately placed on ice. To this mixture, 5 μl of 10× T4 RNA ligase reaction buffer, 0.5 μl of 25 mM ATP, 25 μl PEG8000 (50%), 1.25 μl of 40 mM Co(NH_3_)_6_Cl_3_ solution, 3.75 μl DMSO and 1 μl T4 RNA ligase 1 (high concentration, NEB, M0437M) were added. The reaction mixture was then thoroughly mixed and incubated at 25 °C for 12 h, followed by purification using the DNA Clean and Concentrator kit (Zymo Research), eluting with 20 μl RNase-free water. The eluted cDNA (20 μl) was stored at −80 °C, and 8 μl was used for each 15-cycle PCR amplification. PCR was performed using NEBNext Multiplex Oligos for Illumina (96 unique dual index primer pairs, New England BioLabs, E6440S). The resulting libraries were purified on a 3.5% low-melting-point agarose gel and sequenced on an Illumina NovaSeq 6000 device with a single-end 100-base pair read length. For a more detailed description of the process, see Supplementary Note II.

## Data availability

Sequencing data have been deposited in the Gene Expression Omnibus (GEO) with the following accession numbers: GSE268871 for the human samples, GSE268872 for the maize samples and GSE268873 for the *Arabidopsis* samples. All other data are available in the paper or the Supplementary Information. Source data are provided with this paper.

## Code availability

Reads mapping and m^6^A sites detection scripts are available at GitHub via https://github.com/y9c/m6A-CAMseq (ref. 33).

## Supplementary Material

SI

**Supplementary information** The online version contains supplementary material available at https://doi.org/10.1038/s41557-025-01801-3.

## Figures and Tables

**Fig. 1|:**
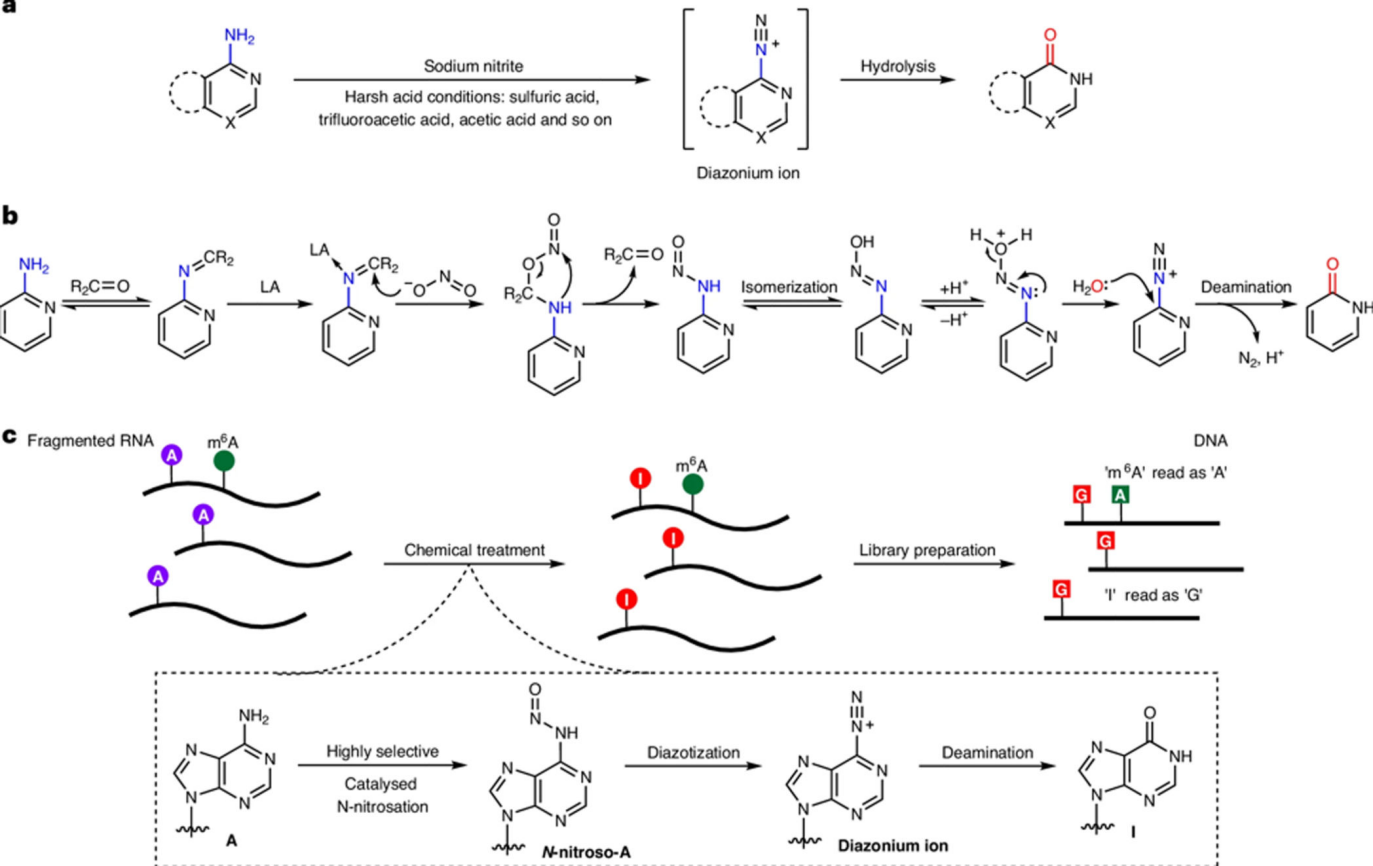
Cooperative deamination of primary amines catalysed by a carbonyl organocatalyst and Lewis acid. **a**, Nitrite ions mediate deamination through a sequential process of nitrosation and diazotization under harsh acidic conditions. **b**, This work and GLORI-seq^13^: cooperative deamination catalyzed by a carbonyl organocatalyst and Lewis acid (LA). **c**, Schematic of the m^6^A-CAM-seq method for whole transcriptome m^6^A sequencing. RNA samples were fragmented and treated under optimized conditions, followed by purification and sequencing library preparation.
