# Supplementary material for: Small-molecule-catalysed deamination enables transcriptome-wide profiling of N6-methyladenosine in RNA
Source: Nat Chem. Author manuscript; Available in PMC 2025 Aug 13. (PMC12345379; doi:10.1038/s41557-025-01801-3)
Supplement: SI [file NIHMS2093314-supplement-SI.pdf]

nature chemistry

Supplementary information https://doi.org/10.1038/s41557-025-01801-3

Small-molecule-catalysed deamination enables transcriptome-wide profiling of N^6-methyladenosine in RNA

In the format provided by the authors and unedited

Supplementary Materials

The file includes:

Materials and Methods

Supplementary Figures 1-18

Supplementary Tables 1-3

Supplementary Notes I & II

Reference

Materials and Methods

Caging and Decaging Kinetics

To visualize and quantify the caging and decaging kinetics, a custom-designed DNA oligonucleotide was purchased from Integrated DNA Technologies (IDT), containing a 5' 6-FAM (Fluorescein) label at the 5' terminus with the sequence 5'-FAM-TGCCAAGACTGTTGAGGAAGATGAGAGAAT-3'. For all assays, the oligo was separated using a 20% PAGE gel and imaged with an iBright 1500 using a 488 nm excitation laser. Fiji (ImageJ) was utilized to calculate the densitometric intensity of each reaction, normalizing it with respect to the fully caged or fully decaged DNA band to estimate percent conversion.

Glyoxal Fully Caging Assays. 0.2 nmol of the test DNA strand was mixed with 14.5 µL of an 8.8 M glyoxal solution (Sigma Aldrich) and 50 µL DMSO. Adopted from a previous protocol[1], reactions were brought to a final volume of 100 µL with nuclease-free water and incubated at 50 °C for 60 minutes. The reaction was precipitated from solution using ethanol and reconstituted in 50 µL of nuclease-free water.

Caging Kinetics Assays. Triplicates of 0.1 nmol of the test DNA strand were mixed with 1.0 µL of a 1.0 M carbonyl compounds solution and 10 µL of 5× sodium cacodylate buffer (pH = 7.0). The reactions were brought to a final volume of 50 µL with nuclease-free water, and incubated at 37 °C for 0, 5, 10, 20, 30, 40, 50, and 60 minutes. At each time point, the reaction was precipitated from solution using ethanol and reconstituted in 50 µL of nuclease-free water. An aliquot of 3 µL of the purified reaction was mixed with 2× RNA loading dye (New England Biolabs) and analyzed via PAGE. Caging half-times were estimated based on pseudo-first-order rate kinetics using a nonlinear curve fit in GraphPad Prism.

Glyoxal Fully Caging Assays. Triplicates of 0.1 nmol of the test DNA strand were mixed with glyoxal solution (10^3, 2×10^3, 4×10^3, 8×10^3, 16×10^3, 32×10^3, 64×10^3, 128×10^3 equiv.) and 10 µL of 5 × sodium cacodylate buffer (pH = 7.0). Reactions were brought to a final volume of 50 µL with nuclease-free water and incubated at 37 °C for 20 minutes. The reactions were precipitated from solution using ethanol and reconstituted in 50 µL of nuclease-free water. An aliquot of 3 µL of each purified reaction was mixed with 2× RNA loading dye (New England Biolabs) and analyzed via PAGE. Caging half-times were estimated based on pseudo-first-order rate kinetics using a nonlinear curve fit in GraphPad Prism.

pH value Caging Kinetic Assays. Triplicates of 0.1 nmol of the test DNA strand were mixed with 1.0 µL of an 8.8 M glyoxal solution or 1.0 µL of a 1.0 M glyoxal solution and 10 µL 5× sodium cacodylate buffer (pH = 5.5, 6.0, 6.5, 7.0, 7.5, 8.0, 8.5, 9.0). Reactions were brought to a final volume of 50 µL with nuclease-free water and incubated at 37 °C for 20 minutes. Reactions were precipitated from solution using ethanol and reconstituted in 50 µL of nuclease-free water. An aliquot of 3 µL of each purified reaction was mixed with 2× RNA loading dye (New England Biolabs) and analyzed via PAGE. Caging half-times were estimated based on pseudo-first-order rate kinetics using a nonlinear curve fit in GraphPad Prism.

Decaging Kinetic Assays. Triplicates of 20 pmol of a fully caged test DNA strand were incubated in a final volume of 50 µL of phosphate buffered saline (137 mM NaCl, 2.7 mM KCl, 8 mM Na_2HPO_4, and 2 mM KH_2PO_4) adjusted to pH 6.5, 7.0, or 7.5 where appropriate. Samples were incubated at 70 °C in a thermal cycler. A 3 µL aliquot was taken at the indicated time points and mixed with 2× RNA loading dye (New England Biolabs) and analyzed via PAGE. Decaging half-times were estimated based on pseudo-first-order rate kinetics using a nonlinear curve fit in GraphPad Prism.

NMR

More details on Supplementary Note I

Sanger Sequencing

The 60 nucleotide (nt) DNA oligo was treated with deamination reagents. Following treatment, the DNA was purified using the DNA Clean & Concentrate kit (Zymo Research). Primers complementary to the 5′ and 3′ flanking regions of the oligo were used for 10 rounds of PCR amplification. The resulting PCR product was then subjected to Sanger sequencing using reverse primers.

Spike-in Probe Synthesis

Spike-in probes were synthesized and pooled according to a previously published protocol[2]. Sequences for spike-in probes are listed in Supplementary Table 7. Probes 1–8 were mixed in a ratio (weight: weight, w: w) of 20%, 15%, 10%, 5%, 5%, 10%, 15% and 20%, respectively. The final probe mixture consists of five sets of UMI-labeled RNA oligos of 0/25/50/75/100% m^6A.

Sequencing library preparation

More details: Supplementary Note II

Sequencing data analysis

Sequencing adapters were trimmed from the 3' ends of both read 1 and read 2 using the Cutadapt tool. Low-quality and short reads were filtered out by applying "-q 20 –max-n 0 -m 20" arguments. 10 nt of a random sequence in the 5' end of read 1 were extracted as a unique molecular barcode for each sequence. The processed reads were then mapped to the human (GRCh38), *Arabidopsis* (TAIR10) and maize (Zm-B73-REFERENCE-NAM-5.0) genomes accordingly using the hisat2-3n tool. Further details can be found at: https://github.com/y9c/m6A-CAMseq.

Statistical methods of m^6A sites detection

Reads mapped using hisat2-3n were filtered using the criteria: "[XM] * 20 <= (qlen - sclen) && [Zf] <= 3 && 3 * [Zf] <= [Zf] + [Yf]". This ensured the removal of reads with more than 5% mismatched aligned bases and excluded clustered reads where either more than 3 unconverted A sites were detected or over one-third of the A sites were unconverted. Then, the probability (p) of random errors was calculated at various sequencing depths and numbers of unconverted events

using the binomial distribution. Only sites with a sequencing depth greater than 20 and a *p*-value less than 10^{-4} were considered significantly methylated and retained for further analysis.

Position importance score of m^6A motifs. Firstly, the expected frequencies of each nucleotide were calculated using sequences in the -10 to -5 window and +5 to +10 window around the m^6A sites. Since different species show different nucleotide frequencies on the gene, especially at the 3' UTR region, normalization of the expected nucleotide frequency ensures a fair interspecies comparison. If $B = \{A, C, G, T\}$ is the set of nucleotides, then for each $b \in B$, the adjusted expected frequency is f_b, and $\sum_{b \in B} E_b = 1$. Let s_i represent the score associated with motif i. For a certain position j, the total score of a given nucleotide b is the sum of s_i for all motifs where position j is nucleotide b, noted as $T_{\{b,j\}}$. $T_{\{b,j\}}$ is then normalized to the relative total score for 4 nucleotides at this position, respectively, noted as $N_{\{b,j\}}$. Then entropy is used to measure the importance of each nucleotide at position j.

$$E_{\{b,j\}} = N_{\{b,j\}} \, log_2(\frac{N_{\{b,j\}}}{f_b})$$

Finally, these importance scores are normalized to calculate a final contribution score for each position. This is done by first adjusting the importance scores by subtracting the minimum importance score at that position from all scores, and then normalizing these adjusted scores so that their sum equals the sum of the original importance scores at that position. The formula for the final contribution score of a position j is:

$$\mathrm{I}_{\{\mathrm{b,j}\}} = \frac{\mathrm{E}_{b,j} - \min\left(\mathrm{E}_{.,j}\right)}{\sum\left(\mathrm{E}_{.,j} - \min\left(\mathrm{E}_{.,j}\right)\right)} \cdot \sum \mathrm{E}_{.,j}$$

This algorithm ensures that the final scores represent the relative importance or contribution of each position within the motifs, factoring in both the distribution of nucleotides and their associated scores.

Supplementary Figures

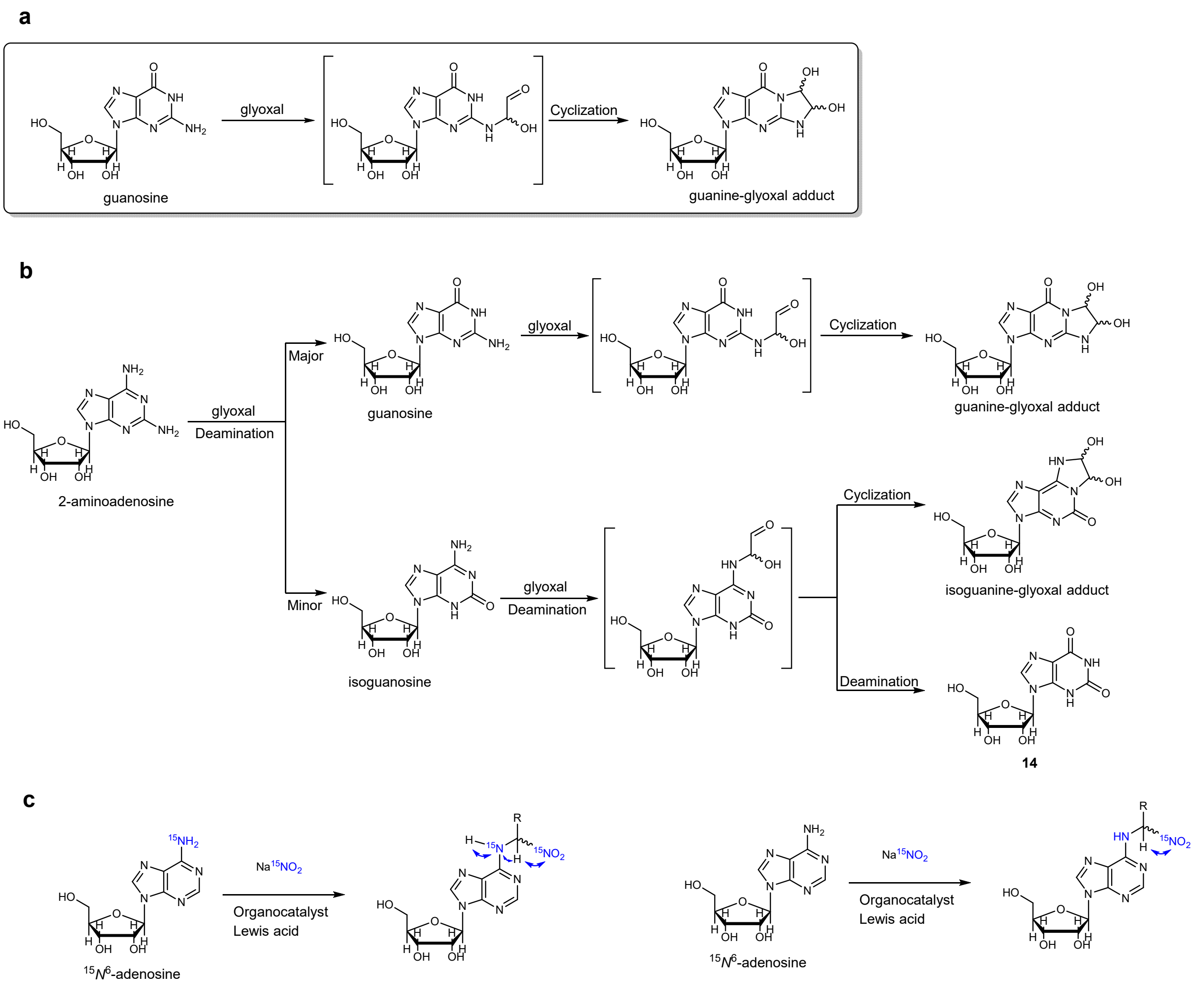

Supplementary Figure 1 | Side products of deamination reaction a, Formation of the major side product, a guanine-glyoxal adduct, which can inhibit the deamination of guanosine. **b**, Side products of 2-aminoadenosine deamination. Deamination of 2-aminoadenosine at the 2-position occurs more rapidly than at the 6-position. The majority of initial deamination events form guanosine, which then produces the guanosine-glyoxal adduct, inhibiting further deamination. A minority of initial deamination events result in isoguanine, leading to the formation of the isoguanine-glyoxal adduct and subsequent deamination product **14**. **c**, ^{15}N-labeled adenosine reacting with ^{15}N-labeled sodium nitrite results in a double ^{1}H-^{15}N HMBC signals (left). In contrast, normal adenosine reacting with ^{15}N-labeled sodium nitrite produces only a single ^{1}H-^{15}N HMBC signal (right).

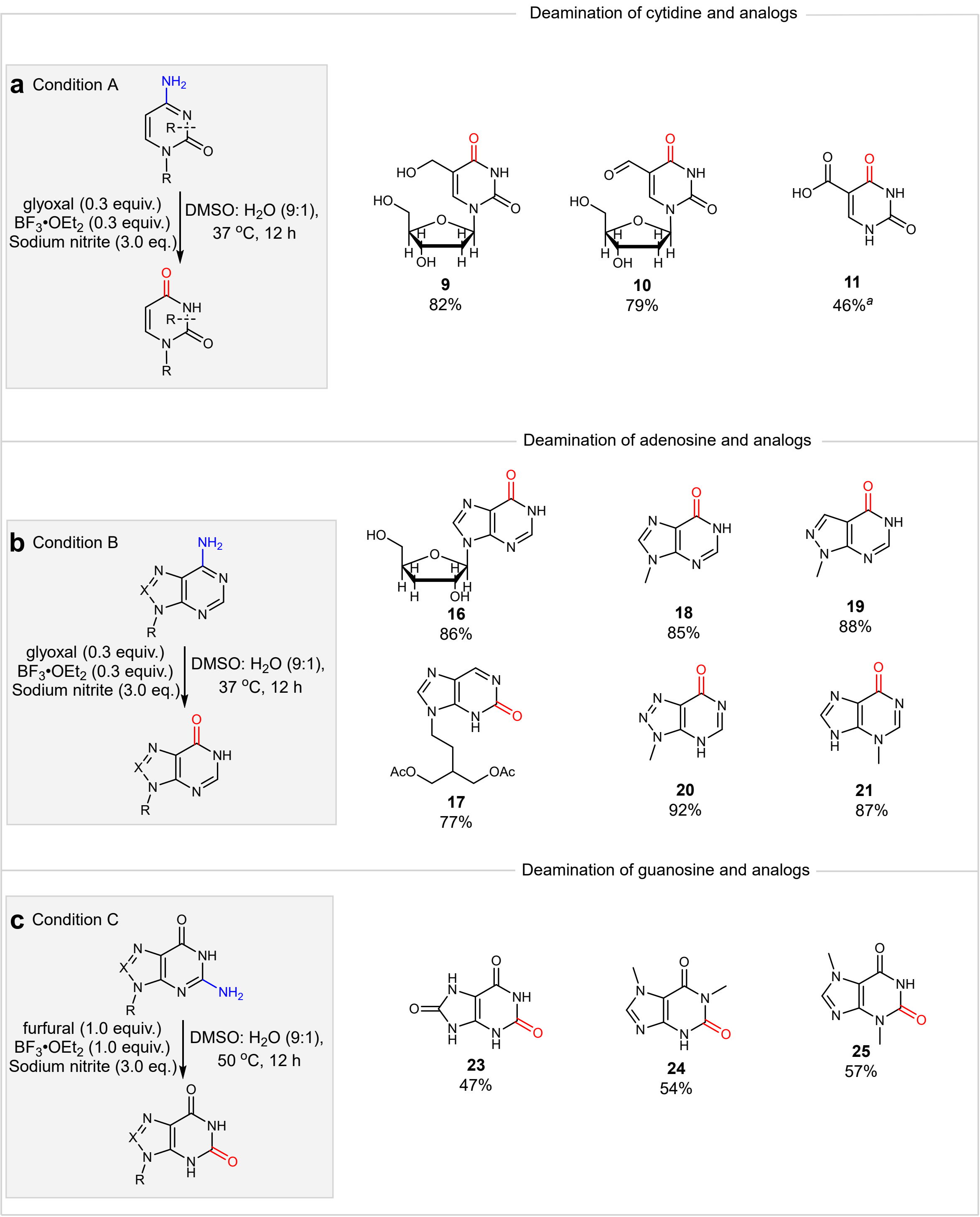

Supplementary Figure 2 | Deamination of nucleobases analogs catalyzed by organocatalyst combined with Lewis acid. a, Substrate scope of deamination reaction with cytidine analogues. **b**, Substrate scope of deamination reaction with adenosine analogues. **c**, Substrate scope of deamination reaction with guanosine analogues using 1.0 equiv. furfural as the organocatalyst. [a]The decarboxylation products after deamination have not been included.

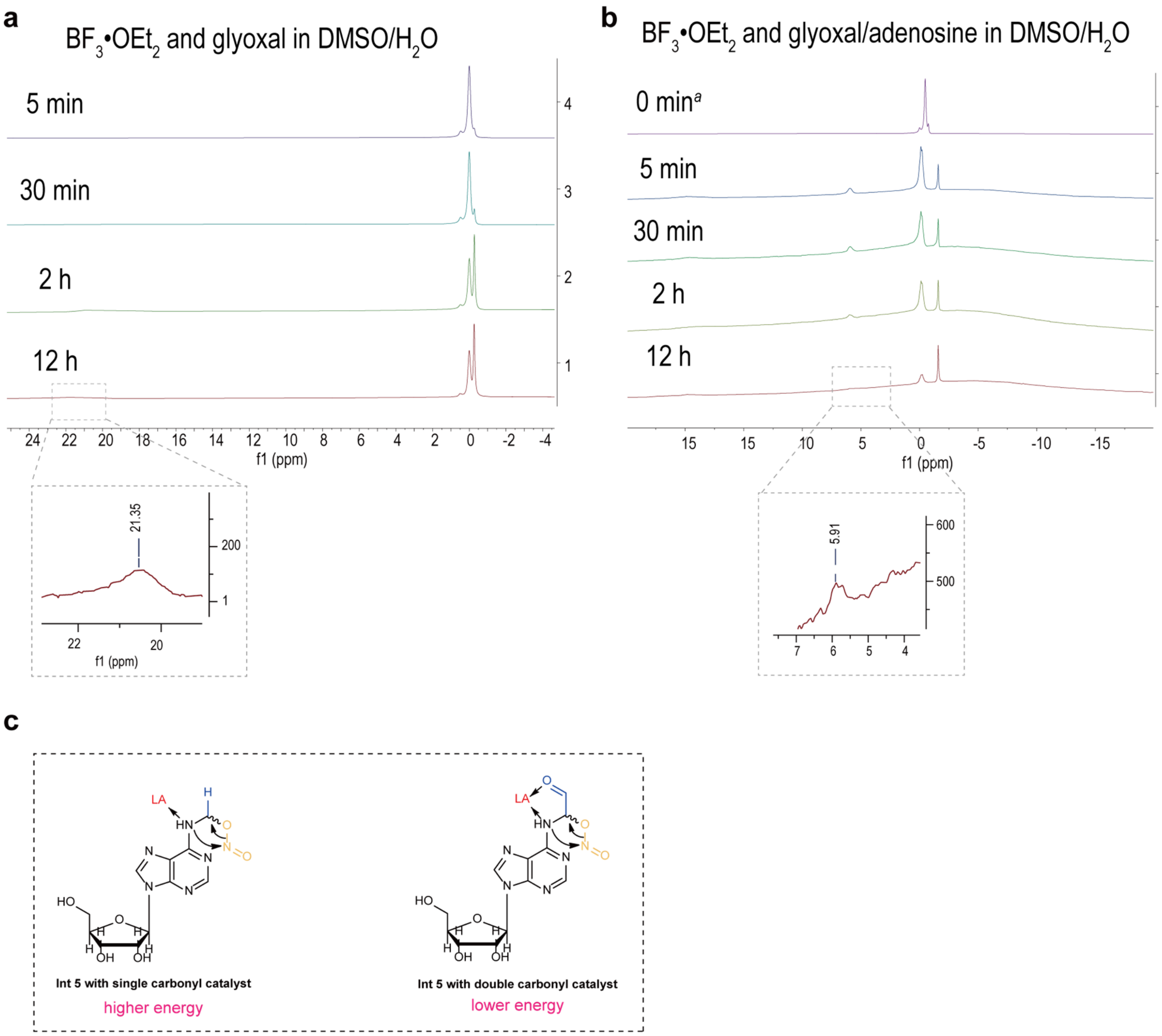

Supplementary Figure 3 | NMR experiments demonstrate X (O, N) → Lewis Acid (LA) interaction. a, ^{11}B NMR experiments demonstrating the interaction between Lewis Acid and organocatalyst. b, ^{11}B NMR experiments demonstrating the interaction between Lewis Acid and adenosine / organocatalyst / imine intermediate. [a]0 min refers to ^{11}B NMR spectrum of BF_3•OEt_3 and glyoxal adduct before the addition of adenosine. c, Proposed model demonstrating that the bicarbonyl catalyst shows enhanced catalytic efficiency compared to the single carbonyl catalyst.

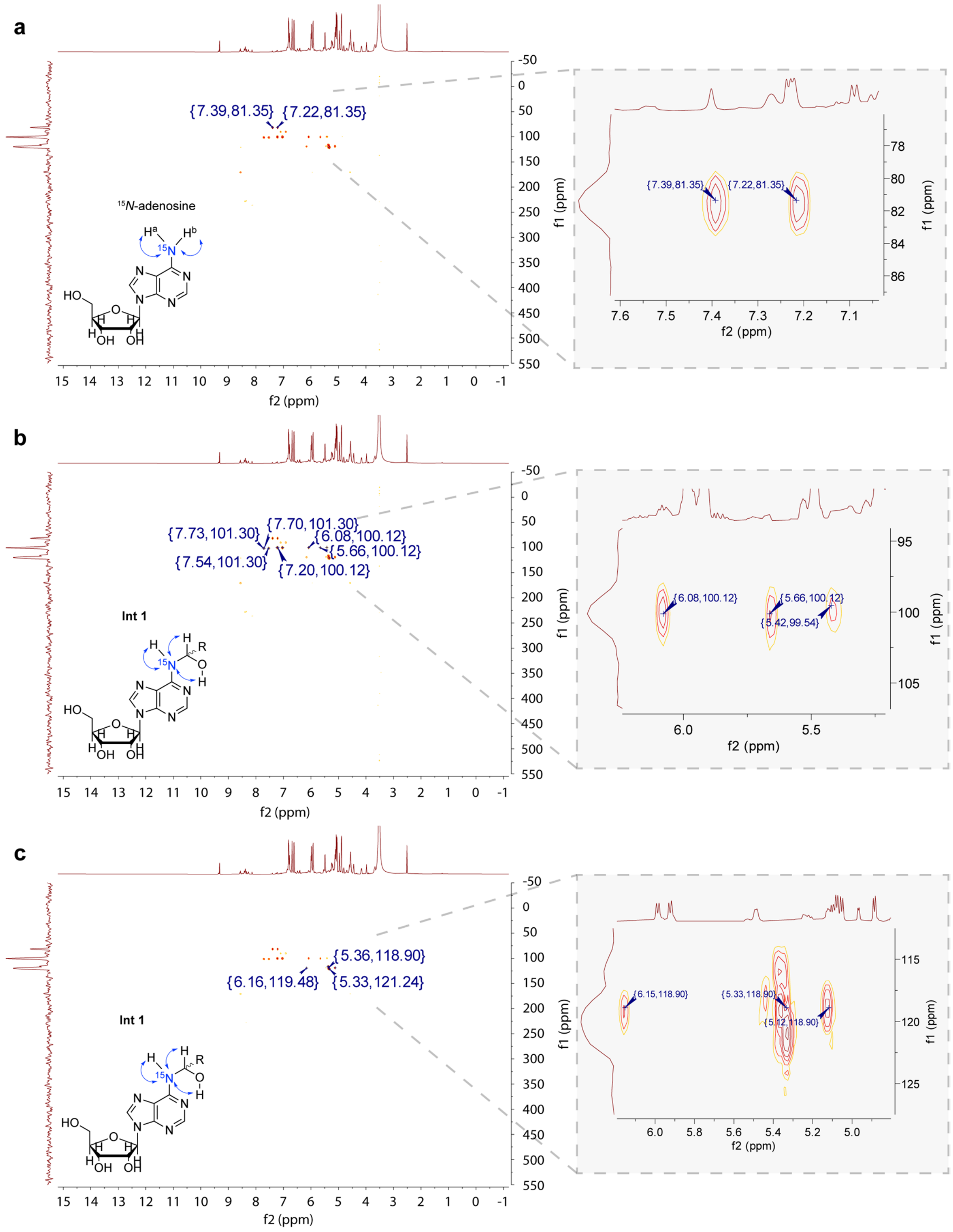

Supplementary Figure 4 | 2D ^{1}H-^{15}N HMBC spectra of $^{15}N^{6}$-adenosine condensation with glyoxal in DMSO-d$_6$/D$_2$O at room temperature. a, ^{1}H-^{15}N HMBC analysis of starting materials residues during the condensation of $^{15}N^{6}$-adenosine and glyoxal without Lewis acid catalysis. **b**, ^{1}H-^{15}N HMBC analysis of the condensation intermediate. **c**, ^{1}H-^{15}N HMBC analysis of potential stereoisomeric condensation intermediates.

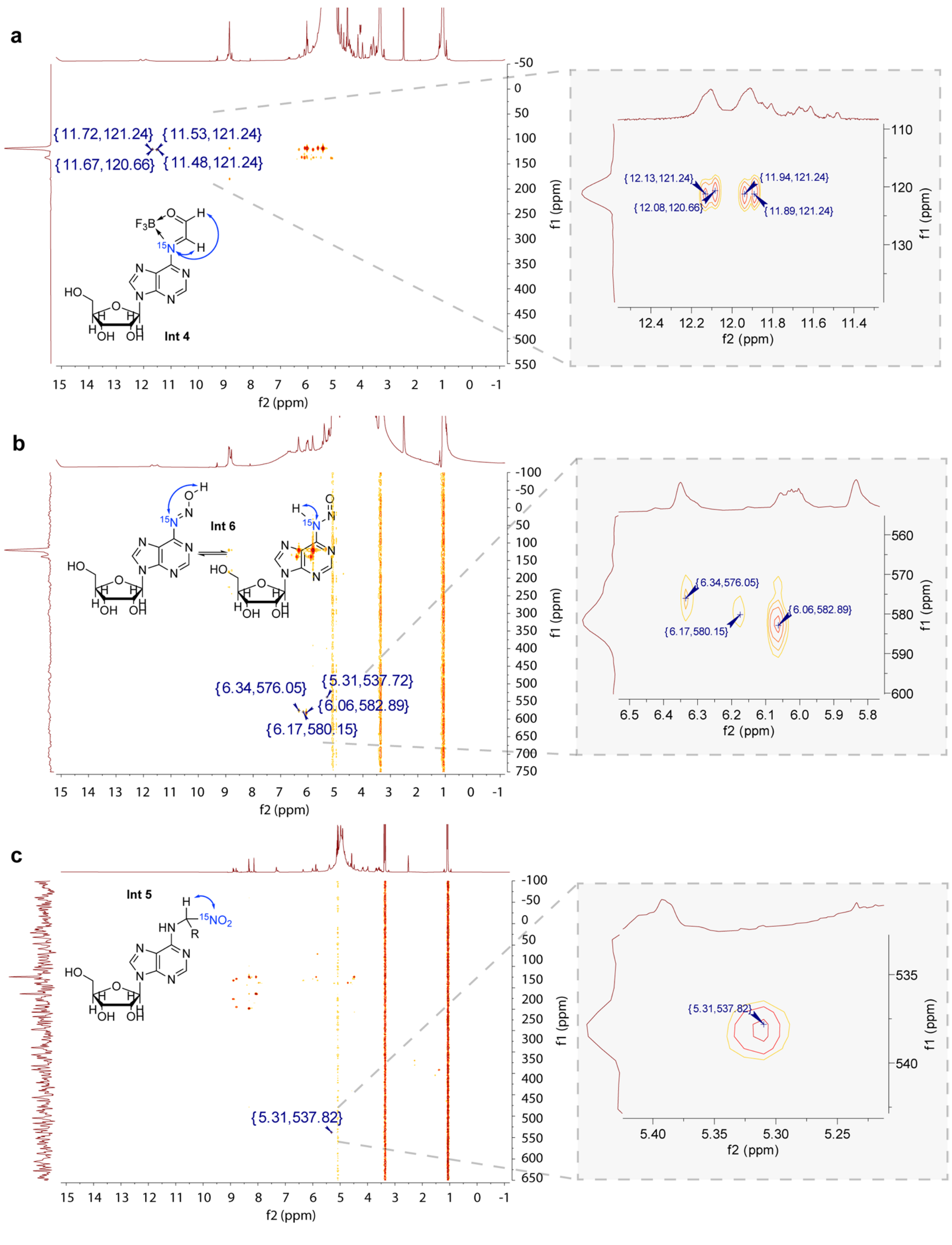

Supplementary Figure 5 | Mechanism study. a, ^{1}H-^{15}N HMBC experiments investigating the condensation of $^{15}N^{6}$-adenosine and glyoxal under Lewis acid catalysis. **b**, ^{1}H-^{15}N HMBC experiments investigating the deamination of the $^{15}N^{6}$-adenosine with $Na^{15}NO_2$ solution. The imine intermediates vanished immediately after the addition of nitrite ions to the reaction system, indicating a rapid reaction rate. Subsequent reactions are dependent on the formation of the imine intermediates. **c**, ^{1}H-^{15}N HMBC experiments of the deamination of adenosine with $Na^{15}NO_2$ solution.

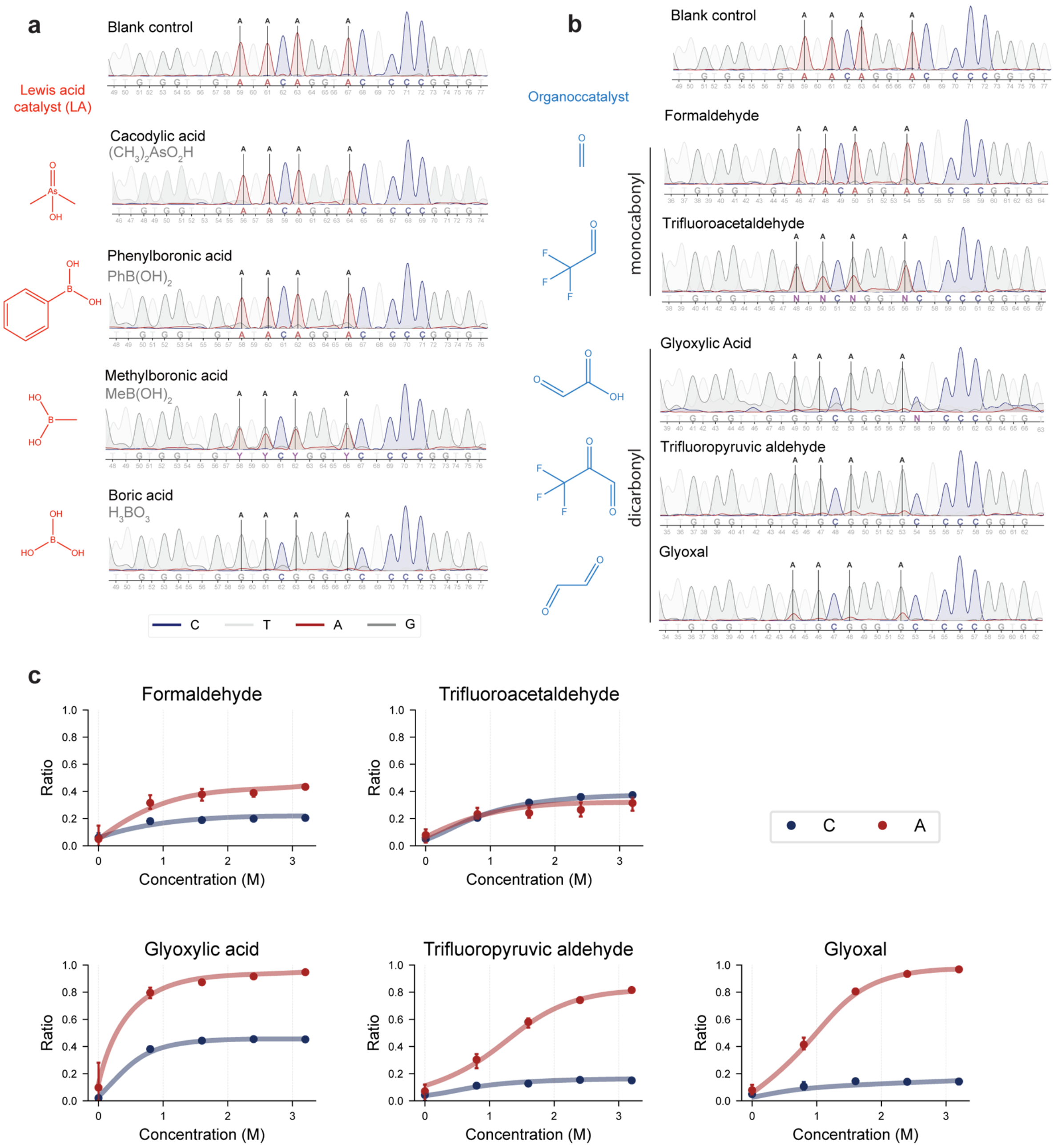

Supplementary Figure 6 | Optimization of CAM-seq reaction using DNA oligo. a, The effect of different Lewis acid catalyst on the A-to-G mutation ratio as shown by Sanger sequencing. Methyl and phenyl groups decrease the capability of accepting an electron pair, thereby reducing catalyst efficiency. Four A sites within this region were marked by vertical lines. **b**, The effect of various carbonyl organocatalysts on the A-to-G mutation ratio as shown by Sanger sequencing. **c**, LC-MS/MS results showing the conversion ratio and selectivity of A under various carbonyl organocatalyst conditions. Dicarbonyl organocatalysts (glyoxal, trifluoropyruvic aldehyde, and glyoxylic acid) exhibit higher selectivity in converting A over C compared to monocarbonyl organocatalysts (formaldehyde, trifluoroacetaldehyde). Data are presented as mean with 95% confidence interval (n = 3 biological replicates).

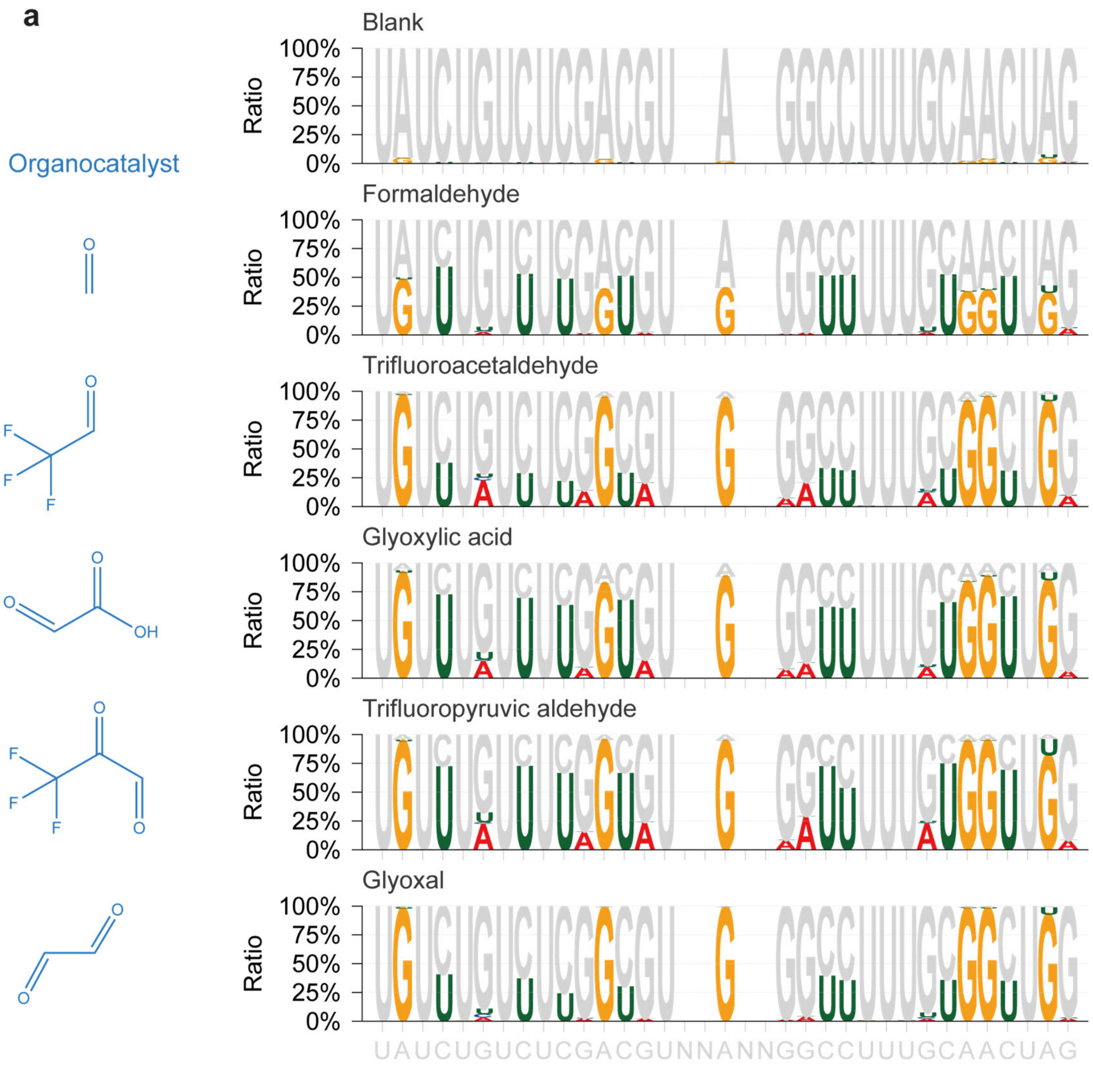

Supplementary Figure 7 | Optimization of A-to-I mutation ratio via a 35-mer RNA probe. a, The mutation ratio of all the nucleotides on a 35-mer RNA probe upon the treatment of various carbonyl organocatalysts. The monocarbonyl catalyst achieved a higher deamination ratio at C sites compared to A sites, demonstrating its potential in C/m^4C detection. The dicarbonyl organocatalyst exhibits greater selectivity in converting A over C, aligning with the LC-MS/MS results.

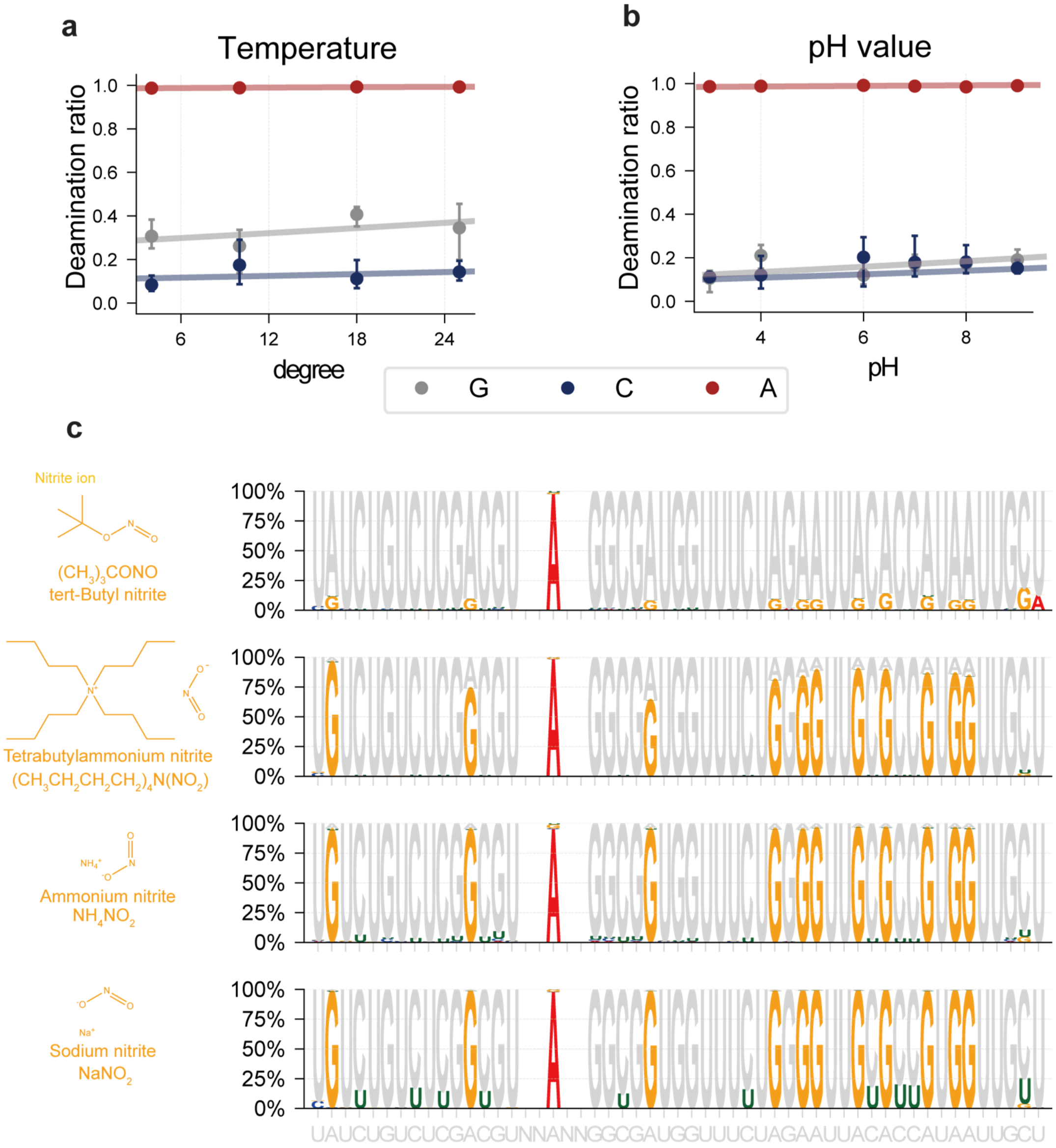

Supplementary Figure 8 | Optimization of deamination conversion conditions via oligos. a, Different ramping temperatures were tested during the deamination reaction, with the conversion ratio of A remaining unchanged from 4°C to 25°C. Data are presented as mean with 95% confidence interval (n = 3 biological replicates). **b**, Different buffers with varying pH values have been used during the deamination step. The conversion of A remaining unchanged from 3 to 9. Data are presented as mean with 95% confidence interval (n = 3 biological replicates). **c**, Different nitrite salts were tested under optimal deamination conditions. Ammonium nitrite and sodium nitrite showed the greatest conversion ratios, with ammonium nitrite exhibiting a lower deamination ratio at C sites, while sodium nitrite achieved a higher conversion ratio at A sites.

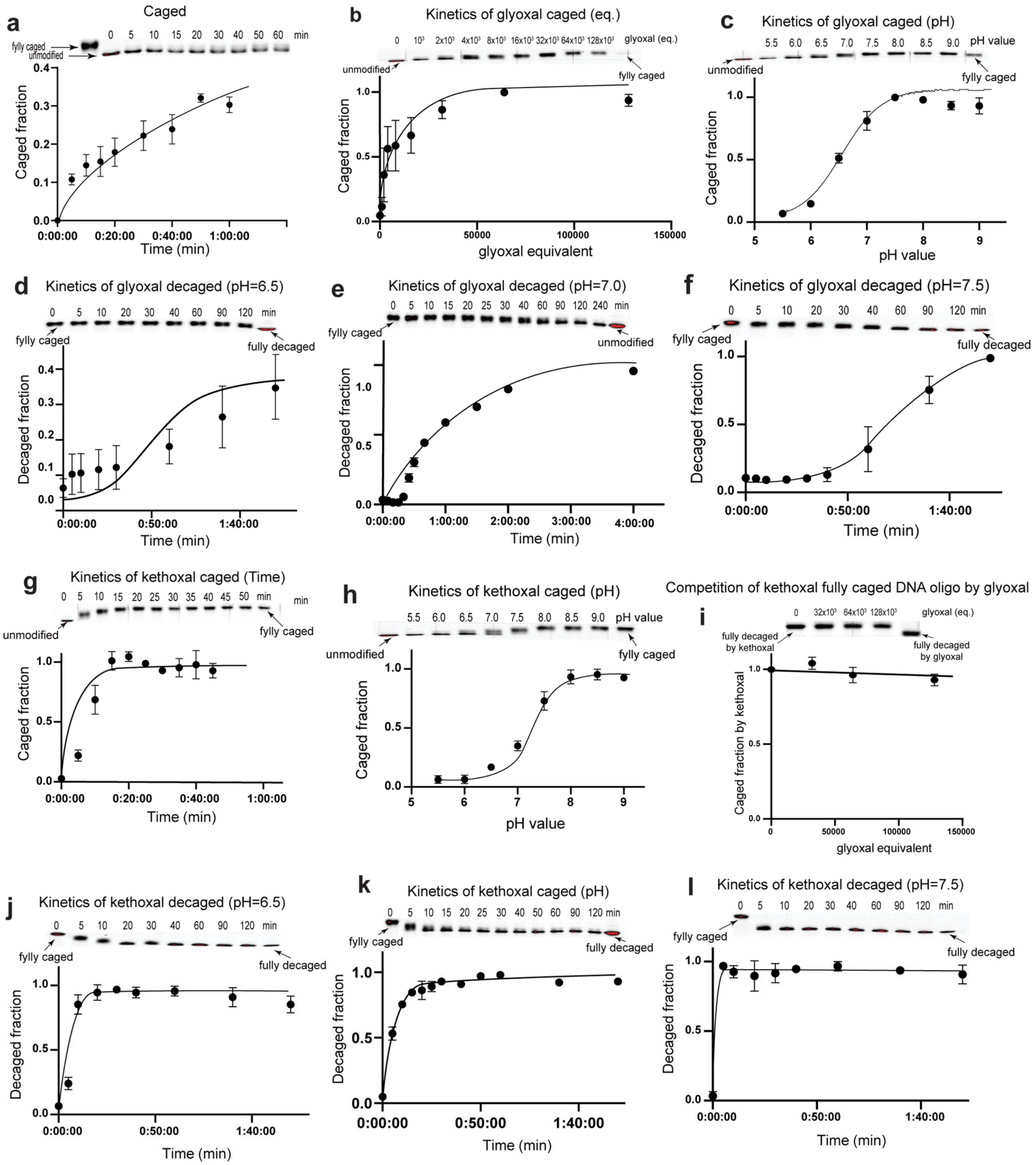

Supplementary Figure 9 | Protection and deprotection reaction of guanine on a FAM labeled oligo. a-**c**, Glyoxal caging assays of a FAM labeled DNA oligo. **a**, Caging kinetics were monitored by treating 100 pmol of a FAM-labeled DNA substrate with 1.0 μmol glyoxal (10^3 equiv.) in 1× sodium cacodylate buffer at 37 °C. At the indicated time points, reactions were analyzed using 20% denaturing PAGE. **b**, Caging kinetics were monitored by treating 100 pmol of a FAM-labeled DNA substrate with glyoxal (10^3, 2 ×10^3, 4 ×10^3, 8 ×10^3, 16 ×10^3, 32 ×10^3, 64 ×10^3, 128×10^3 equiv.) in 1× PBS buffer at 37 °C for 20min and analyzed by using 20% denaturing PAGE. **c**, Caging kinetics

were monitored by treating 100 pmol of a FAM-labeled DNA substrate with 0.2M glyoxal in different PBS buffers (pH = 5.5, 6.0, 6.5, 7.0, 7.5, 8.0, 8.5, 9.0.) at 37 °C for 20min and analyzed using 20% denaturing PAGE. **d-e**, Kinetic assays for decaging glyoxal from a fully caged DNA oligo. **d**, Representative images of the decaging of glyoxal under pH = 6.5 reaction buffer with 20% PAGE analysis. 20 pmol of a fully glyoxal-caged DNA strand was incubated at 70 °C and pH = 6.5 at the indicated times and immediately loaded onto a 20% polyacrylamide gel. Densitometric quantification of caging was measured as a function of time. **e**, Representative images of the decaging of glyoxal under pH = 7.0 reaction buffer with 20% PAGE analysis. 20 pmol of a glyoxal fully caged DNA strand was incubated at 70 °C and pH = 7.0 for the indicated times and immediately loaded onto a 20% polyacrylamide gel. Densitometric quantification of caging was measured as a function of time. **f**, Representative images of the decaging of glyoxal under pH = 7.5 reaction buffer with 20% PAGE analysis. 20 pmol of a glyoxal fully caged DNA strand was incubated at 70 °C and pH = 7.5 for the indicated times and immediately loaded onto a 20% polyacrylamide gel. Densitometric quantification of caging was measured as a function of time. **g-h**, Kethoxal caging assays of a FAM labeled DNA oligo. **g**, Caging kinetics were monitored by treating 100 pmol of a FAM-labeled DNA substrate with 1.0 μmol glyoxal (10^3 equiv.) in 1× sodium cacodylate buffer at 37 °C. Reactions were analyzed using 20% denaturing PAGE at the indicated time points. **h**, Caging kinetics were monitored by treating 100 pmol of a FAM-labeled DNA substrate with 0.2M glyoxal in different PBS buffers (pH = 5.5, 6.0, 6.5, 7.0, 7.5, 8.0, 8.5, 9.0.) at 37 °C for 20min and analyzed using 20% denaturing PAGE. **i**, Testing of fully kethoxal-caged DNA oligo by glyoxal under neutral condition. 20 pmol of a kethoxal fully caged DNA strand was incubated with glyoxal (32×10^3, 64×10^3, 128×10^3 equiv.) in 1× PBS buffer at 37 °C for 20min and analyzed by using 20% denaturing PAGE. **j-l**, Decaging assays of a fully kethoxal-caged DNA oligo. **j**, Representative images of the decaging of kethoxal under pH = 6.5 reaction buffer with 20% PAGE analysis. 20 pmol of a fully glyoxal-caged DNA strand was incubated at 70 °C and pH = 6.5 for the indicated times and immediately loaded onto a 20% polyacrylamide gel. Densitometric quantification of caging was measured as a function of time. **k**, Representative images of the decaging of kethoxal under pH = 7.0 reaction buffer with 20% PAGE analysis. 20 pmol of a fully glyoxal-caged DNA strand was incubated at 70 °C and pH = 7.0 for the indicated times and immediately loaded onto a 20% polyacrylamide gel. Densitometric quantification of caging was measured as a function of time. **l**, Representative images of the decaging of kethoxal under pH = 7.5 reaction buffer with 20% PAGE analysis. 20 pmol of a fully glyoxal-caged DNA strand was incubated at 70 °C and pH = 7.5 for the indicated times and immediately loaded onto a 20% polyacrylamide gel. Densitometric quantification of caging was measured as a function of time.

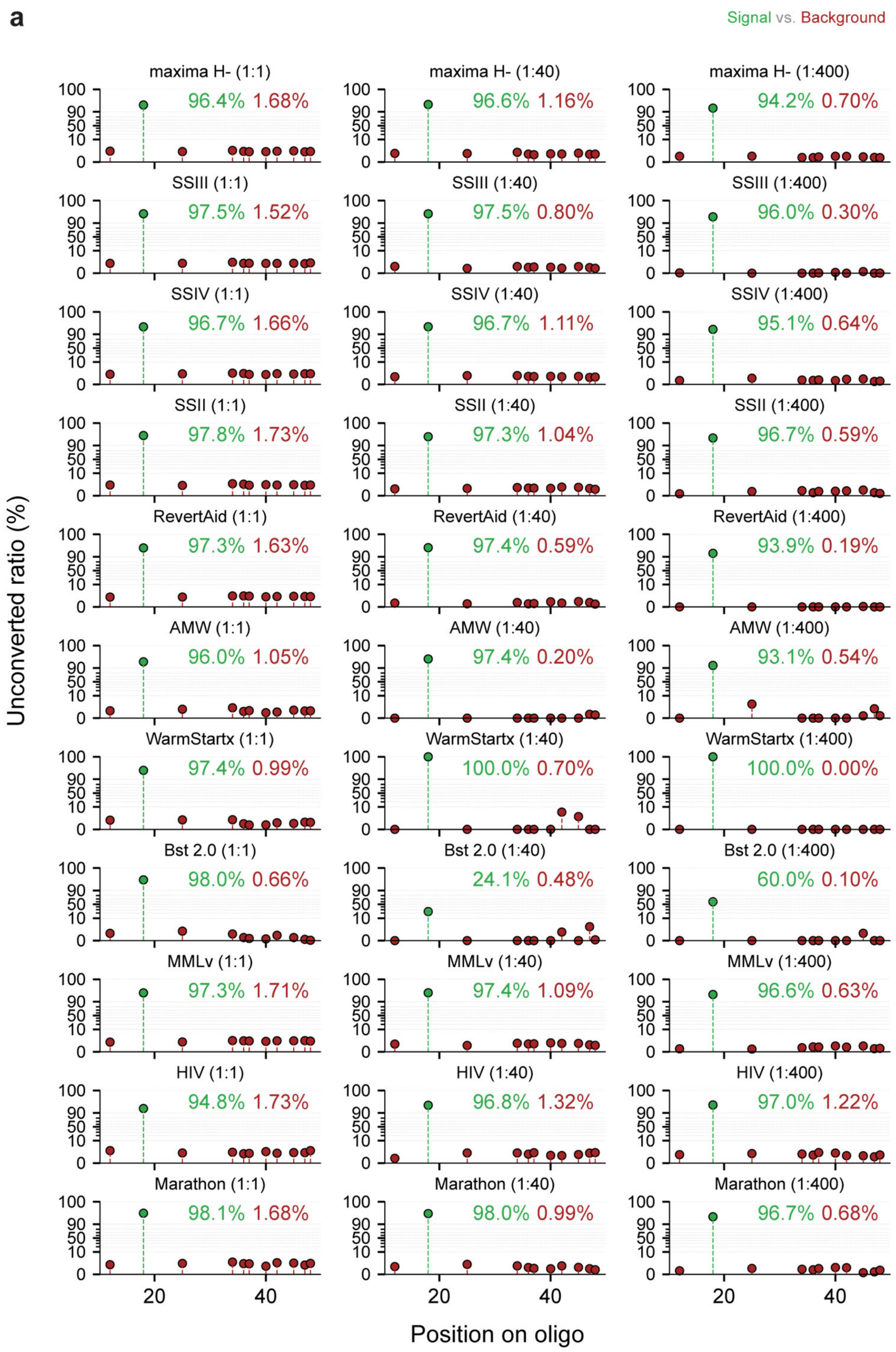

Supplementary Figure 10 | Optimization of reverse transcriptase conditions via an 100% m^{6}A-modified oligo. a, Eleven reverse transcriptase enzymes, with variable dTTP/dCTP ratios, were used in sequencing library preparation. The data for 100% m^{6}A-modified oligo is shown. The x-axis displays the actual modification levels, while the y-axis shows the unconverted ratio observed in sequencing data. The average signal on m^{6}A site (green) and noise on A sites (red) is marked on each panel.

a

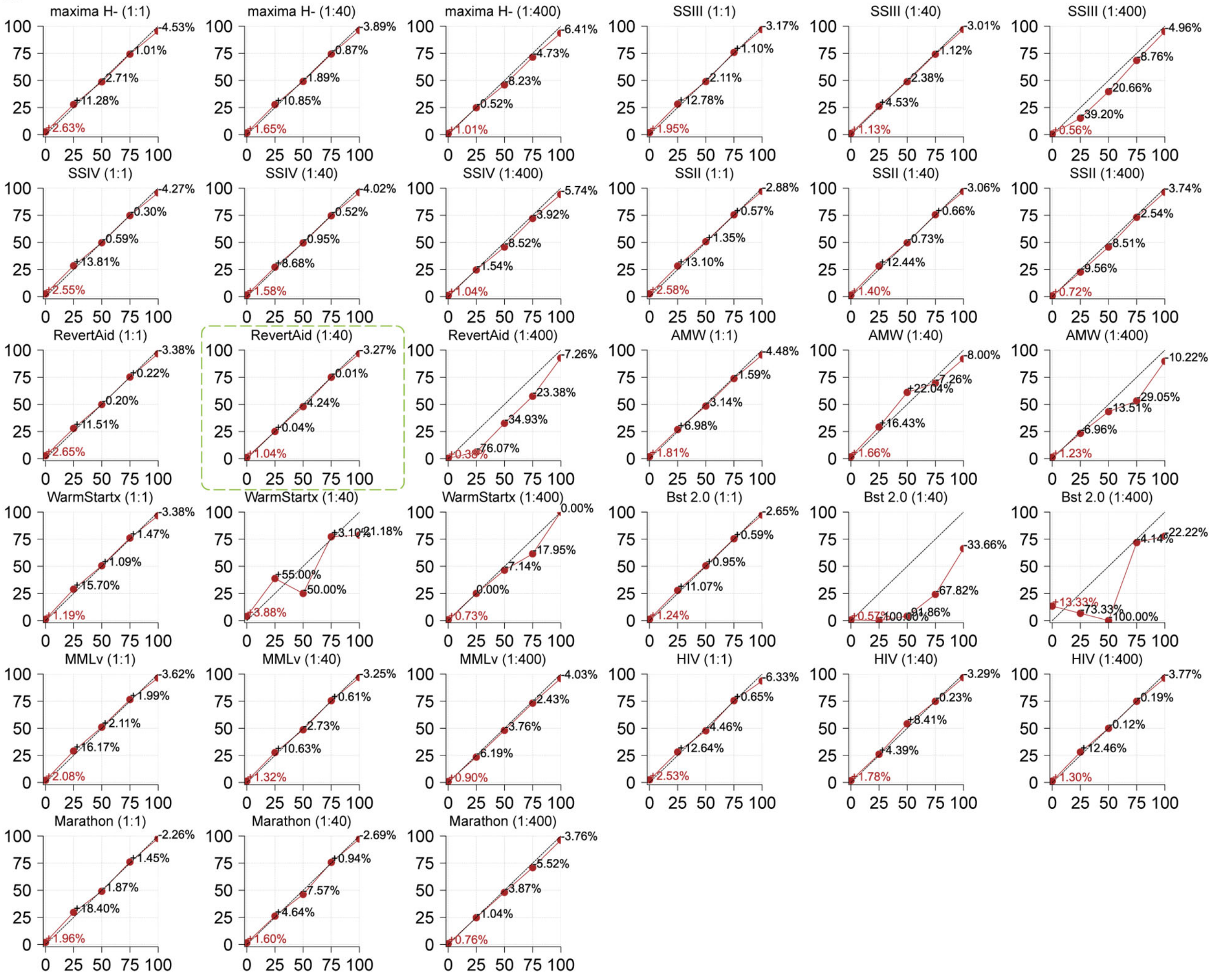

Supplementary Figure 11 | Optimization of reverse transcriptase conditions via spike-in oligo sequencing data. a, Eleven reverse transcriptase enzymes, with variable dTTP/dCTP ratios, were used in sequencing library preparation. Libraries were spiked with 0.05% RNA oligonucleotides at modification levels of 0%, 25%, 50%, 75%, and 100%. The x-axis displays the actual modification levels, while the y-axis shows the modification levels observed in sequencing data. The difference between observed and actual modification levels is indicated on each data point. Positive values indicate an overestimation of methylation levels, whereas negative values suggest underestimation. Background noise for the 0% oligonucleotide is highlighted in red.

a

		GAC	UAG	Ratio
maxima H- RT	1:1	7.49%	1.23%	6.1
	1:40	7.05%	0.72%	9.8
	1:400	6.20%	**0.37%**	16.7
SSIII RT	1:1	7.19%	1.07%	6.7
	1:40	6.17%	0.50%	12.4
	1:400	3.75%	**0.23%**	16.1
SSIV RT	1:1	7.14%	1.16%	6.2
	1:40	6.76%	0.70%	9.7
	1:400	5.98%	**0.35%**	16.9
SSII RT	1:1	7.32%	1.20%	6.1
	1:40	6.80%	0.69%	9.9
	1:400	5.82%	**0.35%**	16.8
RevertAid RT	1:1	7.34%	1.17%	6.3
	1:40	5.74%	**0.36%**	15.9
	1:400	2.06%	**0.13%**	15.6
AMW RT	1:1	6.16%	0.85%	7.2
	1:40	4.57%	**0.43%**	10.7
	1:400	2.61%	**0.21%**	12.4
WarmStart RTx	1:1	5.37%	0.70%	7.7
	1:40	4.85%	**0.43%**	11.4
	1:400	3.53%	**0.19%**	18.1
Bst 2.0	1:1	6.45%	**0.44%**	14.7
	1:40	0.64%	**0.23%**	2.8
	1:400	0.50%	**0.29%**	1.7
MMLv RT	1:1	7.37%	1.14%	6.5
	1:40	6.75%	0.69%	9.8
	1:400	5.78%	**0.36%**	15.9
HIV RT	1:1	6.99%	1.44%	4.9
	1:40	6.85%	1.01%	6.8
	1:400	6.48%	0.79%	8.2
Marathon RT	1:1	7.15%	1.20%	6.0
	1:40	6.39%	0.62%	10.2
	1:400	5.60%	**0.33%**	17.2

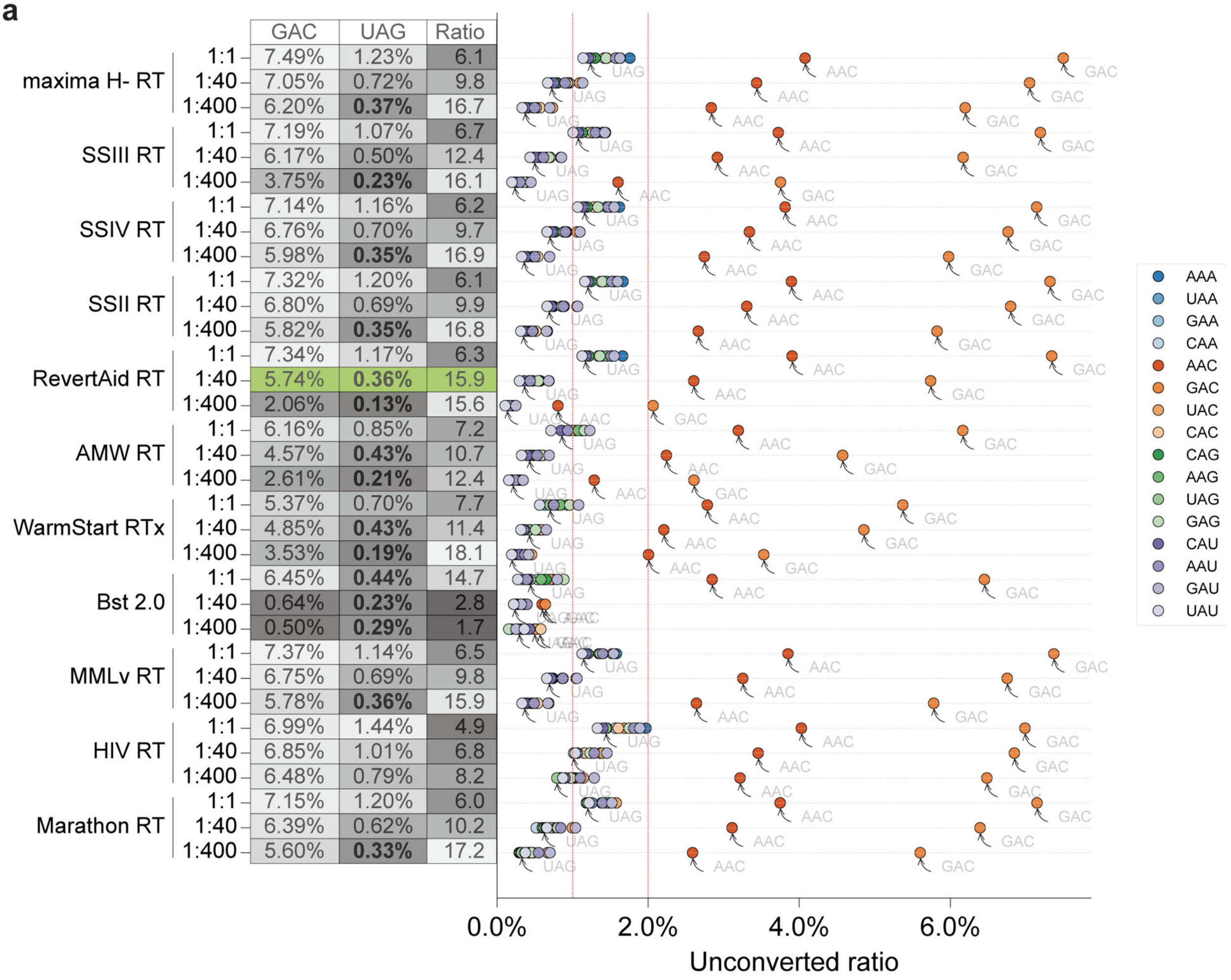

Supplementary Figure 12 | Optimization of reverse transcriptase conditions via mRNA sequencing data. a, Eleven reverse transcriptase enzymes, combined with varying ratios of dTTP/dCTP, were utilized to process human mRNA. The proportion of unconverted adenines (A) in GAC or AAC motifs was considered a true positive signal, while the frequency of unconverted As in UAG motifs and other motifs known for low methylation levels served as an approximate upper limit for false positive signals. Different motifs ($N = 16$) were analyzed and represented in various colors. The table within the figure displays the average ratios of unconverted A sites in GAC and UAG motifs. The relative ratio between these values approximates the signal-to-noise ratio. A standout condition (#14, RevertedAid RT at a 1:40 ratio) demonstrated a background noise level below 0.36%, with a signal-to-noise ratio reaching 15.9.

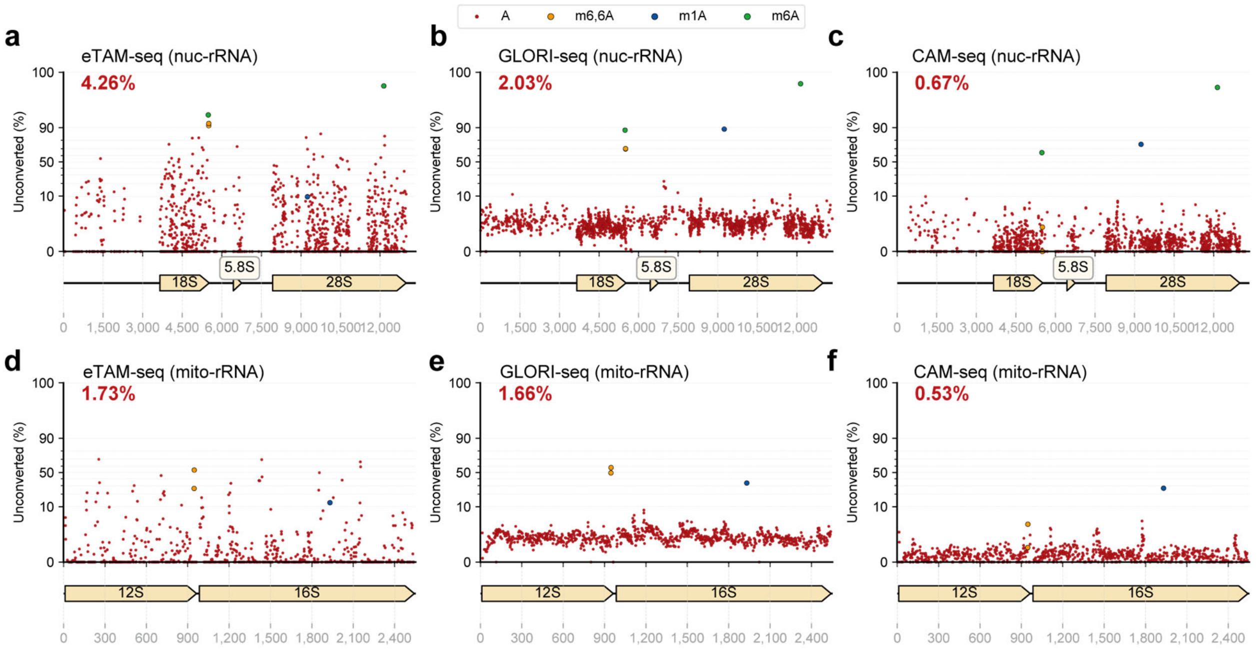

Supplementary Figure 13 | Comparison of deamination-based methods in m^{6}A sequencing using rRNA. a-c, The ratio for unconverted adenine (A) sites with sequencing coverage exceeding 20 on nuclear ribosomal RNA, distinguishing sites modified by m^{6}A (green), m^{1}A (blue), m^{6,6}A(orange), and other A sites (red). The x-axis represents rRNA unit locations, including the 18S, 5.8S, and 28S ribosomal RNA genes, while the y-axis depicts each site's unconverted ratio. Similarly, **d-f**, depict the unconverted ratios for mitochondrial rRNA. The eTAM-seq analysis involved reanalyzing poly(A) RNA from the FTO- sample replicate 1, sourced from GSE201064, utilizing the same pipeline as for CAM-seq. Likewise, for GLORI-seq, data from HEK293T was source from GSE210563.

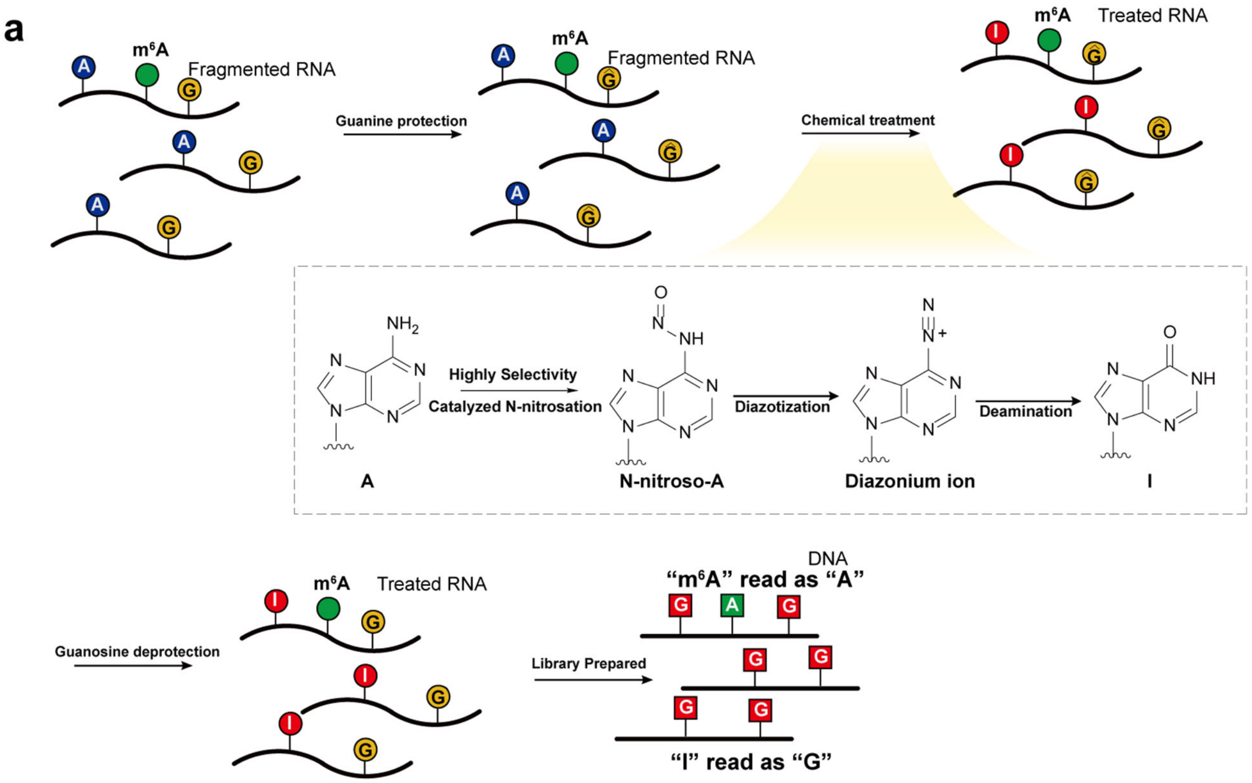

Supplementary Figure 14 | CAM-seq workflow. a, RNA fragments are initially treated with kethoxal to protect guanine (yellow), which prevents caging by glyoxal during the subsequent deamination step. The protected RNA is then treated, where adenine (blue) is catalyzed by a carbonyl organocatalyst to form N-nitroso-A, which subsequently transforms into hypoxanthine through diazotization and deamination. Following the deprotection of guanine, the RNA is purified and prepared for sequencing. Chemical conversion of adenine to guanine is analyzed in sequencing reads to identify unchanged m^{6}A sites. (The protected G site is marked by a caret (^))

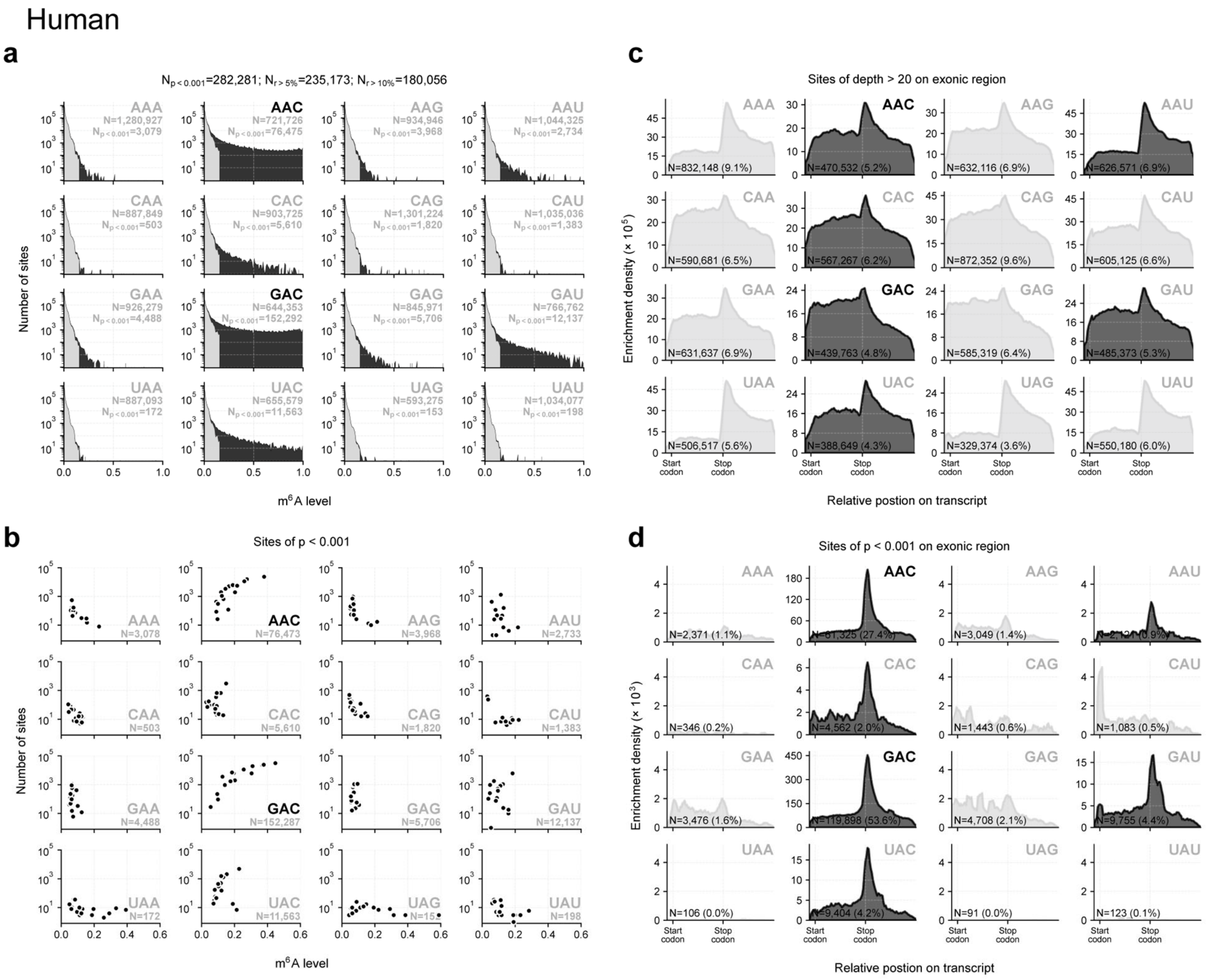

Supplementary Figure 15 | Analysis of m^{6}A sites in human sample across 16 motifs. a, With a sequencing depth cutoff of >20 and p-value <0.001, a total of 331,399 m^{6}A sites were identified in the HEK293T cell (human) transcriptome. Applying detection thresholds of >10% and >20% m^{6}A levels resulted in 272,477 and 200,550 sites, respectively. The total adenine (A) sites with adequate sequencing coverage (>20) are denoted for each motif. The frequency distributions of m^{6}A levels at these sites are presented in individual panels, with sites meeting the p-value threshold (p < 0.001) in black and those not meeting the threshold in gray. The number of detected m^{6}A sites per motif is also indicated. **b**, For tri-nucleotide motifs (e.g., G<u>A</u>C), average m^{6}A levels and site counts were aggregated by their corresponding pentanucleotide motifs (e.g., GG<u>A</u>CT, N=16), illustrating the relationship between m^{6}A levels and site counts for these extended motifs. The total number of m^{6}A sites for each motif is labeled. **c**, A-sites with sufficient sequencing depth (> 20) were plotted relative to their positions within the exonic region of transcripts. This mapping shows the distribution bias of motifs (with or without m^{6}A modifications) along the transcript, with motif abundance and proportions detailed for each panel. **d**, The distribution density of m^{6}A sites along transcripts is shown. The number and relative proportions of m^{6}A sites within these regions are specified.

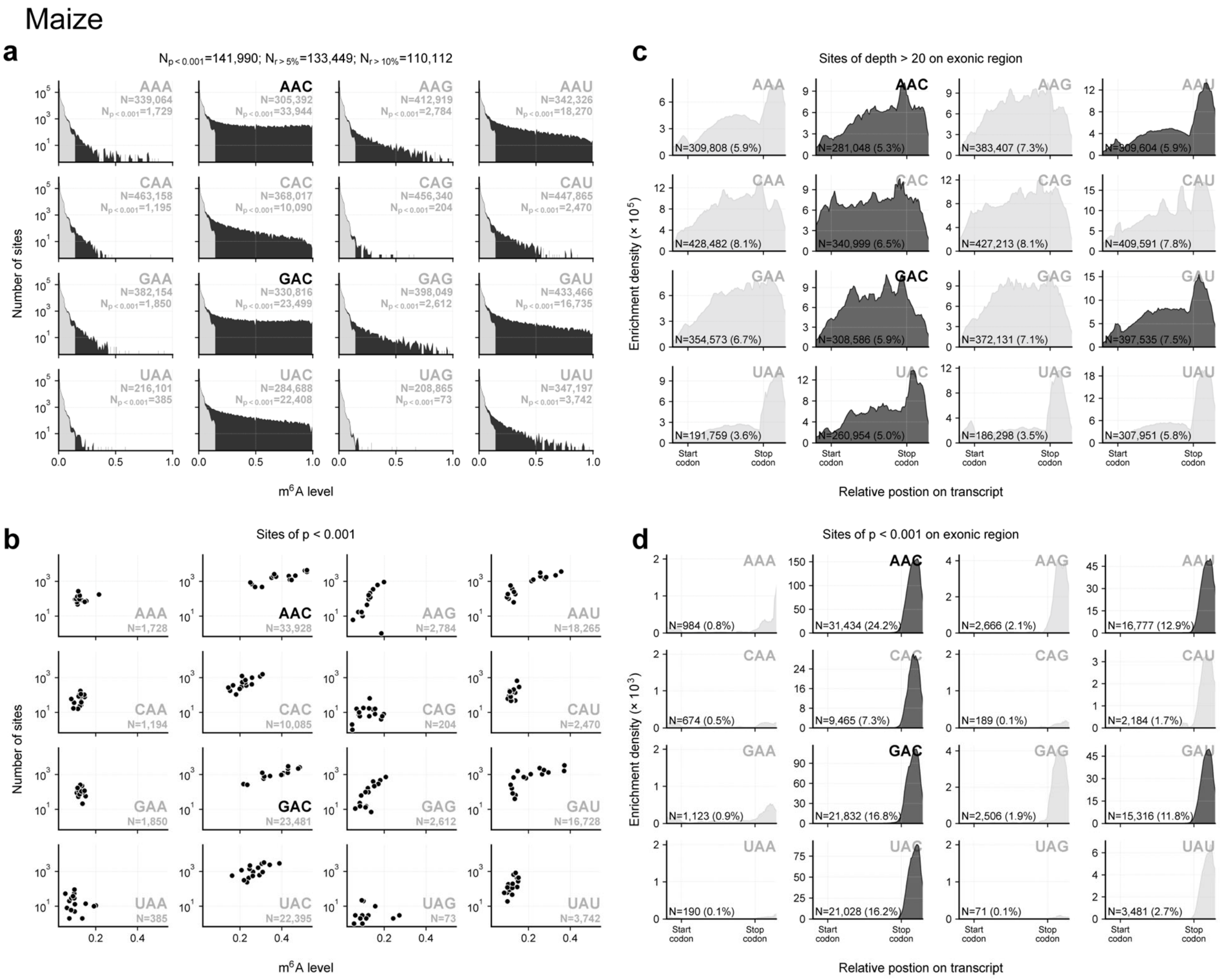

Supplementary Figure 16 | Analysis of m^{6}A sites in *Arabidopsis* sample across 16 motifs. a-d, Similar to **Fig. S5**, data for *Arabidopsis* sample is shown.

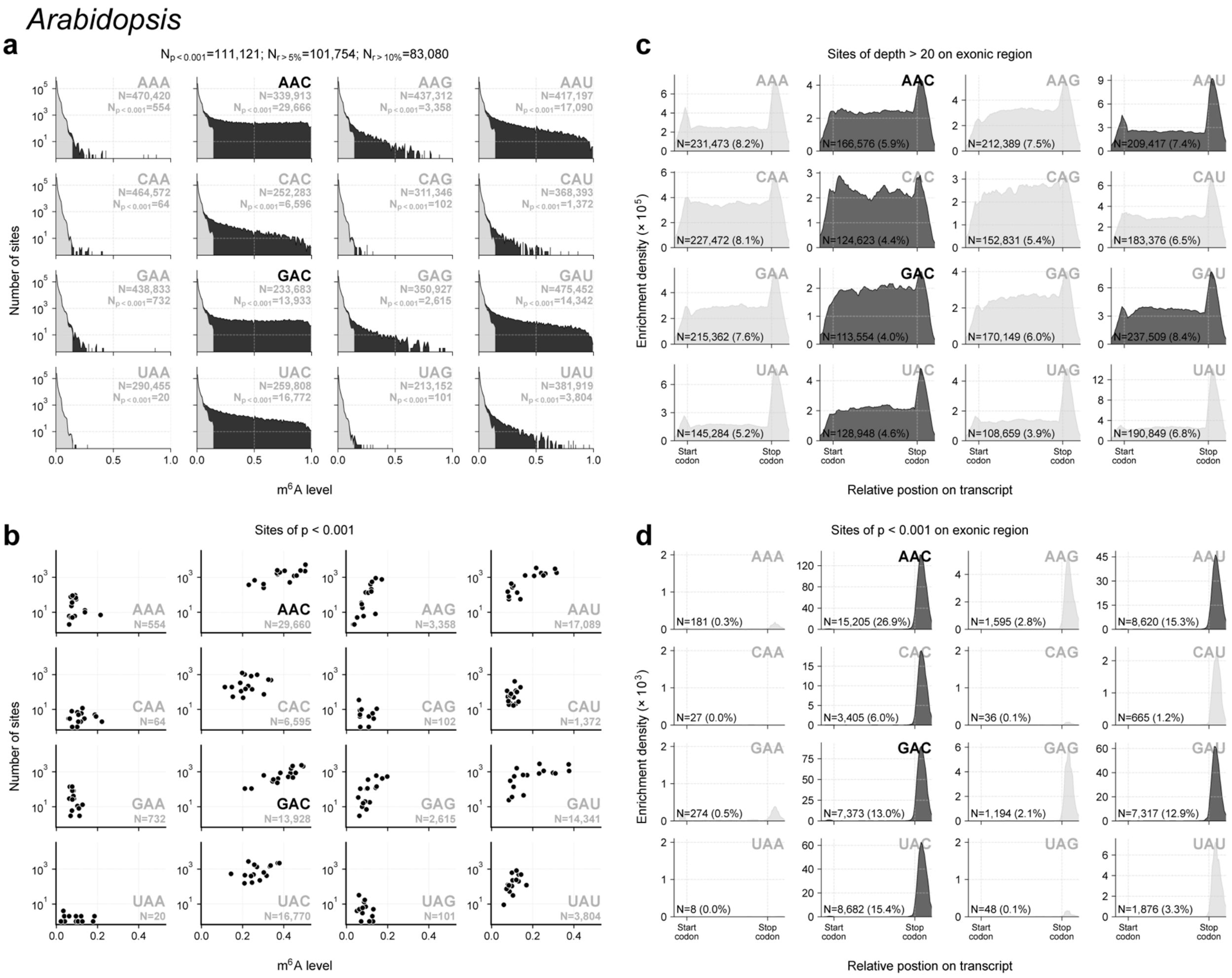

Supplementary Figure 17 | Analysis of m^{6}A sites in maize sample across 16 motifs. a-d, Similar to **Fig. S5**, data for maize sample is shown.

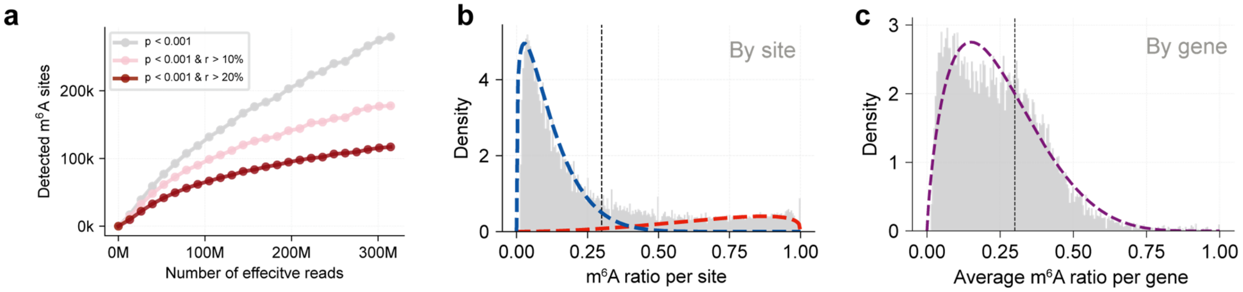

Supplementary Figure 18 | Saturation analysis of m^{6}A sites and classification of m^{6}A-modified genes by maximum modification level. (**A**) Saturation analysis by subsampling m^{6}A sequencing data. High confidential m^{6}A sites (binomial test, *p*-value < 0.001) with additional 10% and 20% of m^{6}A modification levels cutoffs are shown. (**B**) Distribution of m^{6}A modification level of each m^{6}A site from the HEK293T cell line. (**C**) Distribution of average m^{6}A modification level of all the genes.

Supplementary Tables

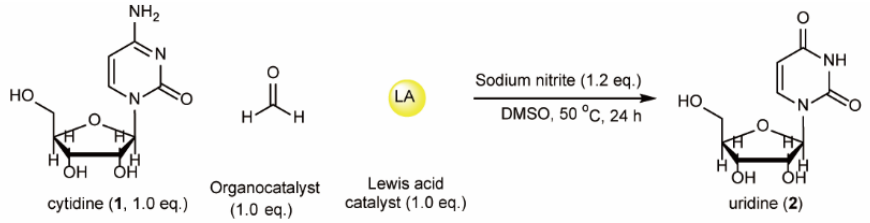

Entry	Organacatalyst	Lewis Acid	Sodium nitrite	Temperature	Time	Yield[a]
1	Formaldehyde	$Sc(OTf)_3$	1.0	25 °C	12 h	Trace
2	Formaldehyde	$Sc(OTf)_3$	1.0	37 °C	12 h	5%
3	Formaldehyde	$Sc(OTf)_3$	1.0	50 °C	12 h	20%
4	Formaldehyde	$Sc(OTf)_3$	2.0	50 °C	12 h	34%
5	-	$Sc(OTf)_3$	2.0	50 °C	24 h	3%
6	Formaldehyde	-	2.0	50 °C	24 h	Trace
7	Formaldehyde	50% $Sc(OTf)_3$	2.0	50 °C	24 h	27%

Supplementary Table 1 | Initial screening of optimal reaction conditions. Standard conditions: **1** (0.10 mmol, 1.0 equiv.), formaldehyde (organocatalyst, 1.0 equiv.), $Sc(OTf)_3$ (Lewis acid catalyst, 1.0 equiv.), sodium nitrite in DMSO (0.5 mL). [a]Yields were determined by LCMS analysis using hypoxanthine as the internal standard.

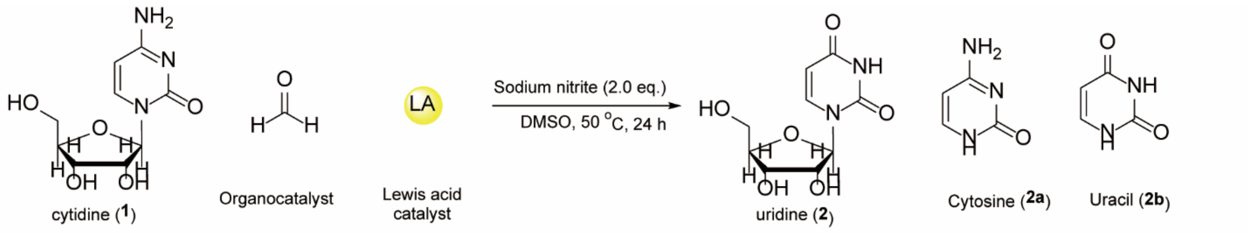

Entry	Organacatalyst	Lewis Acid	Temperature	Time	Conversion[a]	Yield (2)	Yield (2a)	Yield (2b)
1	Formaldehyde	$Sc(OTf)_3$	50 °C	24 h	57%	35%	5%	10%
2	Formaldehyde	$LuCl_3$	50 °C	24 h	19%	10%	2%	7%
3	Formaldehyde	$Y(OTf)_3$	50 °C	24 h	Trace	Trace	ND	ND
4	Formaldehyde	$FeCl_3$	50 °C	24 h	Trace	Trace	ND	ND
5	Formaldehyde	$YbCl_3$	50 °C	24 h	Trace	Trace	ND	ND
6	Formaldehyde	$RuCl_3$	50 °C	24 h	95%	Trace	Trace	Trace
7	Formaldehyde	$Ln(OTf)_3$	50 °C	24 h	25%	15%	2%	7%
8	Formaldehyde	$CuCl_2$	50 °C	24 h	Trace	Trace	ND	ND
9	Formaldehyde	$NiCl_2$	50 °C	24 h	Trace	Trace	ND	ND
10	Formaldehyde	$CoCl_2$	50 °C	24 h	Trace	Trace	ND	ND
11	Formaldehyde	$MnCl_2$	50 °C	24 h	Trace	Trace	ND	ND
12	Formaldehyde	$AlCl_3$	50 °C	24 h	23%	5%	2%	11%
13	Formaldehyde	$Sm(OTf)_3$	50 °C	24 h	17%	3%	2%	10%
14	Formaldehyde	$InCl_3$	50 °C	24 h	25%	Trace	Trace	Trace
15	Formaldehyde	AuCl	50 °C	24 h	Trace	Trace	ND	ND
16	Formaldehyde	$BF_3{\bullet}OEt_2$	50 °C	24h	68%	50%	3%	10%
17	Formaldehyde	$B(OCH_2CF_3)_3$	50 °C	24 h	Trace	Trace	ND	ND
18	Formaldehyde	$B(OH)_3$	50 °C	24 h	Trace	Trace	ND	ND
19	Formaldehyde	$B(C_6F_5)_3$	50 °C	24 h	10%	7%	Trace	Trace
20	Formaldehyde	$BF_3{\bullet}OEt_2$	37 °C	24 h	56%[b]	43%	3%	3%
21	Formaldehyde	$BF_3{\bullet}OEt_2$	37 °C	12 h	52%[c]	45%	1%	2%
22	Formaldehyde	$BF_3{\bullet}OEt_2$	37 °C	6 h	43%[d]	40%	Trace	Trace
23	Formaldehyde	$BF_3{\bullet}OEt_2$	37 °C	6 h	33%[e]	30%	Trace	Trace

Supplementary Table 2 | Optimal conditions screening for Lewis acid. Standard conditions: **1** (0.10 mmol, 1.0 equiv.), formaldehyde (0.1 mmol, 1.0 equiv.), Lewis acid catalyst (0.1 mmol, 1.0 equiv.), sodium nitrite (2.0 equiv.) in DMSO (0.5 mL). [a]Conversion were determined by LC/MS analysis using hypoxanthine as the internal standard. [b]Decreasing the reaction temperature reduces the deglycosylation byproduct. [c]Decreasing the Reaction time can reduce the deglycosylation byproduct. [d]Reducing the reaction time to 6 hours slightly reduces the yield of the target product. [e]Reducing the amount of sodium nitrite to 1.0 equiv. reduces the yield of the deamination product.

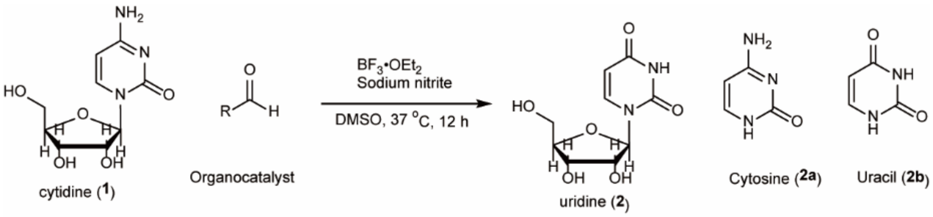

Entry	Organacatalyst	Conversion[a]	Yield (2)	Yield (2a)	Yield (2b)
1	Formaldehyde	52%	45%	1%	2%
2	Trifluoracetaldehyde	45%	40%	1%	3%
3	Trifluoropyruvaldehyde	87%	36%	21%	27%
4	Glyoxal	> 99%	85%	3%	10%
5	2-Pyridinecarboxaldehyde	43%	38%	Trace	2%
6	2-Formylpyrrole	32%	22%	Trace	3%
7	2-Thiophenecarboxaldehyde	45%	44%	Trace	Trace
8	8-Formylquinoline	41%	36%	Trace	2%
9	Furfural	63%	58%	Trace	Trace
10	Glyoxal	> 99%[b]	95%	Trace	Trace
11	Glyoxal	> 99%[c]	95%	Trace	Trace
12	Glyoxal	90%[d]	90%	Trace	Tace
13	Glyoxal	> 99%[e]	95%	Trace	Trace
14	Glyoxal	96%[f]	93%	ND	ND
15	Glyoxal	89%[g]	87%	ND	ND

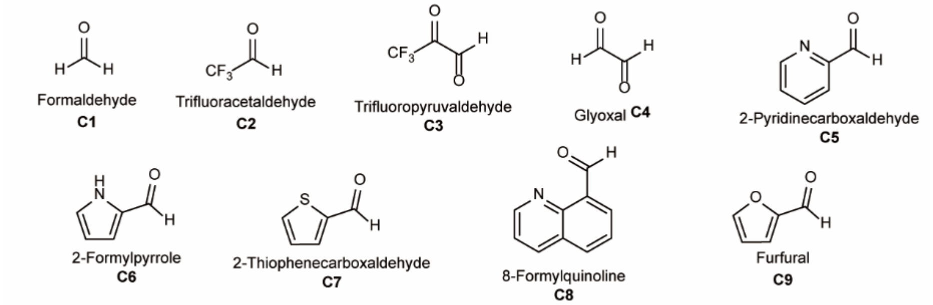

Supplementary Table 3 | Optimal conditions screening for organocatalysts. Standard conditions: **1** (0.10 mmol, 1.0 equiv.), organocatalyst (0.1 mmol, 1.0 equiv.), BF_3•Et_2O (0.1 mmol, 1.0 equiv.), sodium nitrite (2.0 equiv.) in DMSO (0.5 mL) at 37 °C for 12 hours. Yields were determined by LC-MS analysis using hypoxanthine as the internal standard. [a]Conversion was determined by LC-MS analysis using hypoxanthine as the internal standard. [b]Using a 20:1 DMSO: H_2O mixture solution. [c]Using a 9:1 DMSO: H_2O mixture solution. [d]Using a 5:1 DMSO: H_2O mixture solution. [e]Lewis acid catalyst and organocatalyst quantities were reduced to 0.5 equivalents each in a mixed solvent system of 9:1 DMSO: water. [f]Lewis acid catalyst and organocatalyst quantities were reduced to 0.3 equivalents each in a mixed solvent system of 9:1 DMSO: water. [g]Lewis acid catalyst and organocatalyst quantities were reduced to 0.2 equivalents each in a mixed solvent system of 9:1 DMSO: water.

Supplementary Note I: NMR

A. General Remarks

All purchased solvents and substrates were used without further treatment unless otherwise stated. Yields refer to chromatographically and spectroscopically (^{1}H NMR) homogeneous material, unless otherwise stated. The reactions were run in 4 mL vials under ambient air unless otherwise noted. Chemical reactions were monitored by LC/MS (Agilent 6130 ESI LC-MS), and thin layer chromatography (TLC). Analytical thin-layer chromatography (TLC) was conducted using 0.2 mm commercial silica gel plates (silica gel 60, F_{254}, EMD chemical). Analytical reversed-phase thin-layer chromatography (TLC) was carried out using 0.2 mm commercial C18 silica gel plates (silica gel 60 RP-18, $F_{254}S$). Vials (15 × 45 mm 1 dram (4 mL) /17 × 60 mm 3 dram (7.5 mL) with PTFE lined cap attached) were purchased from Qorpak and used as received. Nuclear magnetic resonance spectra (^{1}H NMR and ^{13}C NMR) were recorded with Bruker Model DMX 500 (500 MHz, ^{1}H at 500 MHz, ^{13}C at 126 MHz) or 400 (400 MHz, ^{1}H at400 MHz, ^{13}C at 101 MHz). Unless otherwise noted, all spectra were acquired in DMSO-d_6. Chemical shifts are reported in parts per million (ppm, δ) and are referenced to the residual solvent (DMSO, δ=2.50 ppm (^{1}H) and 39.52 ppm (^{13}C)). Coupling constants were reported in Hertz (Hz). Data for 1 H NMR spectra were reported as follows: chemical shift (ppm, referenced to protium, s = singlet, d = doublet, t = triplet, q = quartet, quin = quintet, oct = octet, dd = doublet of doublets, td = triplet of doublets, ddd = doublet of doublet of doublets, m = multiplet, coupling constant (Hz), and integration). Column chromatography was performed using E. Merck silica (60, particle size 0.043–0.063 mm), and pTLC was performed on Merck silica plates (60F-254). High-resolution mass spectra (HRMS) were recorded on an Agilent 6530 LC Q-TOF mass spectrometer using electrospray ionization with fragmentation voltage set at 75-130 V and processed with an Agilent MassHunter Operating System.

B. Detection of Side Product

General Procedure: To a 4 mL vial the substrate nucleobase (0.1 mmol, 1.0 equiv.) and mixture solvent DMSO: H_2O (0.45 mL DMSO and 0.05 mL H_2O) was added in air. The organocatalyst (glyoxal, 0.03 mmol, 0.3 equiv.) and Lewis acid catalyst (BF_3•Et_2O, 0.03 mmol, 0.3 equiv.) were then added in one portion at room temperature, followed by the addition of sodium nitrite (25 µL, saturated solution in water, 3.0 equiv.). The reaction vial was sealed again and incubated at 37 °C for 12 hours. The crude reaction mixture was then concentrated under reduced pressure (in vacuo) and purified by flash column chromatography on silica gel to obtain the side product.

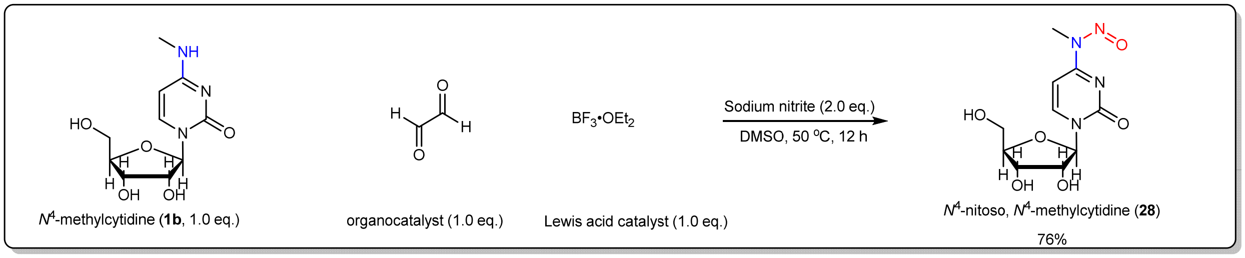

Side product with N^4-methylcytidine: ***N***

Side product with N^4-methylcytidine: **N^4-nitroso, N^4-methylcytidine (28):** Proceed according to the general procedure, with a 76% isolated yield (21.7 mg) as a slightly yellow solid.

R_f = 0.7 (DCM: MeOH = 10 : 1)

^{1}H-NMR (500 MHz, DMSO) δ 8.62 (d, J = 7.5 Hz, 1H), 7.13 (d, J = 7.5 Hz, 1H), 5.82 (d, J = 3.0 Hz, 1H), 5.58 (d, J = 5.0 Hz, 1H), 5.22 (t, J = 5.0 Hz, 1H), 5.08 (d, J = 6.0 Hz, 1H), 4.05 (td, J = 4.9, 2.9 Hz, 1H), 4.02 – 3.98 (m, 1H), 3.97 – 3.92 (m, 1H), 3.78 (ddd, J = 12.1, 5.0, 2.7 Hz, 1H), 3.63 (ddd, J = 12.3, 4.9, 2.9 Hz, 1H), 3.30 (s, 3H).

^{13}C NMR (126 MHz, DMSO) δ 165.03, 154.62, 145.83, 93.39, 90.96, 84.75, 75.06, 69.00, 60.20, 27.61.

HRMS (ESI-TOF) *m/z*: Calcd for $C_{10}H_{15}N_4O_6^+$ $[M+H]^+$: 287.0986, found: 287.0967.

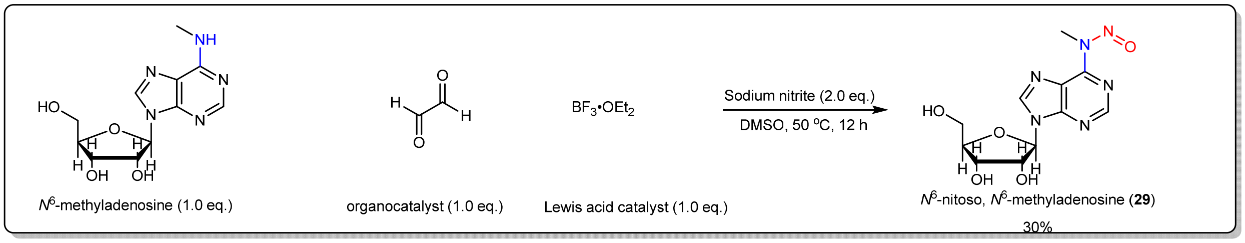

Side product with N^6-methyladenosine: **N^6-nitroso, N^6-methyladenosine (29):** Proceed according to the general procedure in 30% isolated yield (9.5 mg) as a slightly yellow solid.

R_f = 0.6 (DCM: MeOH = 10: 1)

^{1}H-NMR (500 MHz, DMSO) δ 8.94 (s, 1H), 8.89 (s, 1H), 6.12 (d, J = 5.5 Hz, 1H), 5.58 (d, J = 6.0 Hz, 1H), 5.26 (d, J = 5.1 Hz, 1H), 5.12 (t, J = 5.5 Hz, 1H), 4.62 (q, J = 5.5 Hz, 1H), 4.21 (q, J = 5.0 Hz, 1H), 4.00 (q, J = 3.9 Hz, 1H), 3.72 (dt, J = 12.0, 4.9 Hz, 1H), 3.59 (s, 4H).

^{13}C NMR (126 MHz, DMSO) δ 153.67, 151.91, 151.57, 145.66, 124.22, 88.24, 86.15, 74.41, 70.65, 61.59, 29.57.

HRMS (ESI-TOF) *m/z*: Calcd for $C_{11}H_{15}N_6O_5^+$ [M+H]$^+$: 311.1098, found: 311.1124.

C. Experimental Procedure, Isolation, and Characterization

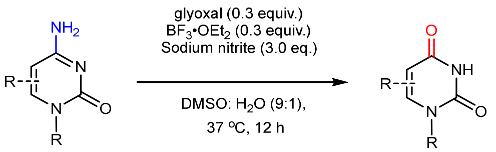

Standard reaction condition for cytidine analogs: To a 4 mL vial the substrate nucleobase (0.1 mmol, 1.0 equiv.) and mixture solvent DMSO: H_2O (0.45 mL DMSO and 0.05 mL H_2O) was added in air. The organocatalyst (glyoxal, 0.03 mmol, 0.3 equiv.) and Lewis acid catalyst (BF_3•Et_2O, 0.03 mmol, 0.3 equiv.) were then added in one portion at room temperature, followed by the addition of sodium nitrite (25 μL, saturated solution in water, 3.0 equiv.). The reaction vial was sealed and incubated at 37 °C for 12 hours. The crude reaction mixture was then concentrated under reduced pressure (in vacuo) and purified by flash column chromatography on silica gel to obtain the side product.

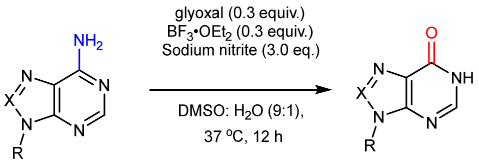

Standard reaction condition for adenosine analogs: To a 4 mL vial the substrate nucleobase (0.1 mmol, 1.0 equiv.) and mixture solvent DMSO: H_2O (0.45 mL DMSO and 0.05 mL H_2O) was added in air. The organocatalyst (glyoxal, 0.03 mmol, 0.3 equiv.) and Lewis acid catalyst (BF_3•Et_2O, 0.03 mmol, 0.3 equiv.) was then added in one portion at room temperature. After that, followed by the addition of sodium nitrite (25 μL, saturated solution in water, 3.0 equiv.). The reaction vial was sealed and incubated at 37 °C for 12 hours. The crude reaction mixture was then concentrated under reduced pressure (in vacuo) and purified by flash column chromatography on silica gel to obtain the side product.

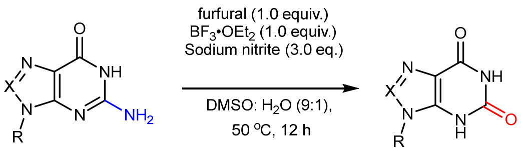

Standard reaction condition for guanosine analogs: To a 4 mL vial the substrate nucleobase (0.1 mmol, 1.0 equiv.) and mixture solvent DMSO: H_2O (0.45 mL DMSO and 0.05 mL H_2O) was added in air. The organocatalyst (2-furfural, 0.10mmol, 1.0 equiv.) and Lewis acid catalyst

(BF_3•Et_2O, 0.1 mmol, 1.0 equiv.) was then added in one portion at room temperature. After that, followed by the addition of sodium nitrite (16.6 μL, saturated solution in water, 2.0 equiv.). The reaction vial was sealed again and incubated at 50 °C for 6 hours before the second addition of catalysts. A solution of the organocatalyst (2-furfural, 0.05mmol, 0.5 equiv.) and Lewis acid catalyst (BF_3•Et_2O, 0.05 mmol, 0.5 equiv.) in 0.1 mL DMSO was added into the reaction, followed by the addition of sodium nitrite (8.4 μL, saturated solution in water, 1.0 equiv.) under ambient atmosphere. The reaction was then stirred at 50 °C for another 6 hours. The crude reaction mixture was then concentrated under reduced pressure (in vacuo) and purified by flash column chromatography on silica gel or C18 reversed phase silica gel to obtain the side product.

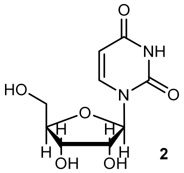

2

Uridine (2): Cytidine (CAS No. 65-46-3) was used as the starting material. Prepared according to the standard reaction condition for cytidine analogs in 91% isolated yield (22.1 mg) as a white powder. NMR data is consistent with the literature report[3].

R*f* = 0.3 (DCM/MeOH = 15:1).

¹H-NMR (500 MHz, DMSO) δ 11.30 (s, 1H), 7.88 (d, J = 8.1 Hz, 1H), 5.78 (d, J = 5.5 Hz, 1H), 5.64 (d, J = 8.1 Hz, 1H), 5.36 (d, J = 5.9 Hz, 1H), 5.07 (dd, J = 5.2, 2.8 Hz, 2H), 4.02 (q, J = 5.4 Hz, 1H), 3.96 (q, J = 4.6 Hz, 1H), 3.84 (q, J = 3.5 Hz, 1H), 3.62 (ddd, J = 12.0, 5.3, 3.3 Hz, 1H), 3.54 (ddd, J = 12.0, 5.1, 3.3 Hz, 1H).

¹³C NMR (126 MHz, DMSO) δ 163.58, 151.22, 141.19, 102.22, 88.14, 85.30, 74.00, 70.35, 61.31.

HRMS (ESI-TOF) *m/z*: Calcd for $C_9H_{13}N_2O_6^+$ $[M+H]^+$: 245.0768, found: 245.0765.

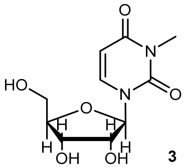

3

3-Methyluridine (3): 3-Methylcytidine (CAS No. 2140-64-9) was used as the starting material. Prepared according to the standard reaction condition for cytidine analogs in 92% isolated yield (23.5 mg) as a white powder. NMR data is consistent with the literature report[4].

R*f* = 0.3 (DCM/MeOH = 15:1).

¹H-NMR (500 MHz, DMSO) δ 7.96 (d, J = 8.1 Hz, 1H), 5.82 (d, J = 5.0 Hz, 1H), 5.77 (d, J = 8.1 Hz, 1H), 5.38 (d, J = 5.6 Hz, 1H), 5.15 – 5.06 (m, 2H), 4.03 (q, J = 5.1 Hz, 1H), 3.96 (q, J = 5.1 Hz, 1H), 3.89 – 3.82 (m, 1H), 3.65 (ddd, J = 12.0, 5.2, 3.1 Hz, 1H), 3.56 (ddd, J = 12.0, 5.0, 3.1 Hz, 1H), 3.16 (s, 3H).

¹³C NMR (126 MHz, DMSO) δ 162.59, 151.44, 139.45, 101.17, 89.40, 85.24, 74.25, 70.11, 61.10, 27.70.

HRMS (ESI-TOF) *m/z*: Calcd for $C_{10}H_{15}N_2O_6^+$ $[M+H]^+$: 259.0925, found: 259.0927.

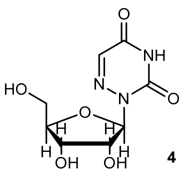

4

6-Azauridine (4): 6-Azacytidine (CAS No. 3131-60-0) was used as the starting material. Prepared according to the standard reaction condition for cytidine analogs in 87% isolated yield (21.2 mg) as a slightly yellow powder. NMR data is consistent with the literature report[5].

$\mathbf{R}_f$ = 0.3 (DCM/MeOH = 15:1).

^{1}H-NMR (400 MHz, DMSO) 12.24 (s, 1H), 7.56 (s, 1H), 5.88 (d, J = 3.9 Hz, 1H), 5.30 (d, J = 5.3 Hz, 1H), 5.08 (d, J = 5.9 Hz, 1H), 4.68 (t, J = 5.8 Hz, 1H), 4.22 (q, J = 5.2 Hz, 1H), 4.00 (q, J = 5.4 Hz, 1H), 3.79 (q, J = 5.6 Hz, 1H), 3.50 (dt, J = 11.8, 4.9 Hz, 1H), 3.38 (q, J = 5.9 Hz, 1H).

^{13}C NMR (101 MHz, DMSO) δ 156.49, 148.35, 136.31, 89.28, 84.55, 72.22, 70.29, 61.95.

HRMS (ESI-TOF) *m/z*: Calcd for $C_8H_{12}N_3O_6^+$[M+H]$^+$: 246.0721, found: 246.0746.

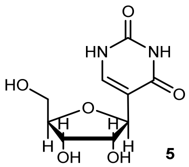

5

Pseudouridine (5): Pseudoisocytidine (CAS No. 57100-18-2) was used as the starting material. Prepared according to the standard reaction condition for cytidine analogs in 91% isolated yield (22.1 mg) as a white powder. NMR data is consistent with the literature report[6].

$\mathbf{R}_f$ = 0.3 (DCM/MeOH = 10:1).

^{1}H-NMR (400 MHz, DMSO) δ 11.11 (s, 1H), 10.88 (s, 1H), 7.52 (d, J = 0.8 Hz, 1H), 4.91 (d, J = 5.1 Hz, 1H), 4.82 – 4.75 (m, 1H), 4.69 (d, J = 5.7 Hz, 1H), 4.46 (dd, J = 4.5, 0.7 Hz, 1H), 3.91 (q, J = 4.8 Hz, 1H), 3.86 (q, J = 5.5 Hz, 1H), 3.73 – 3.65 (m, 1H), 3.59 (ddd, J = 11.8, 4.7, 3.2 Hz, 1H), 3.44 (ddd, J = 11.8, 6.4, 3.7 Hz, 1H).

^{13}C NMR (101 MHz, DMSO) δ 163.65, 151.13, 139.75, 111.12, 83.11, 78.85, 73.86, 70.34, 61.09.

HRMS (ESI-TOF) *m/z*: Calcd for $C_9H_{13}N_2O_6^+$[M+H]$^+$: 245.0768, found: 245.0769.

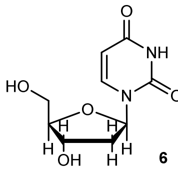

6

2’-Deoxyluridine (6): 2'-Deoxycytidine (CAS No. 951-77-9) was used as the starting material. Prepared according to the standard reaction condition for cytidine analogs in 91% isolated yield (20.7 mg) as a white powder. NMR data is consistent with the literature report[7].

$\mathbf{R}_f$ = 0.50 (DCM/MeOH = 10:1).

^{1}H-NMR (400 MHz, DMSO) δ 11.27 (s, 1H), 7.84 (d, J = 8.1 Hz, 2H), 6.15 (t, J = 6.8 Hz, 2H), 5.63 (d, J = 8.1 Hz, 2H), 5.25 (d, J = 4.2 Hz, 1H), 5.01 (t, J = 5.2 Hz, 1H), 3.77 (q, J = 3.8 Hz, 2H), 3.55 (q, J = 4.9 Hz, 2H).

^{13}C NMR (101 MHz, DMSO) δ 163.62, 150.92, 141.00, 102.22, 87.87, 84.60, 70.89, 61.75, 40.14.

HRMS (ESI-TOF) *m/z*: Calcd for $C_9H_{12}N_2O_5^+$ $[M+H]^+$: 229.0810, found: 229.0805.

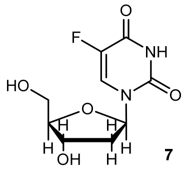

7

5-Fluoro-2’-deoxyluridine (7): 5-Fluoro-2'-deoxycytidine (CAS No. 10356-76-0) was used as the starting material. Prepared according to the standard reaction condition for cytidine analogs in 90% isolated yield (22.5 mg) as a white powder. NMR data is consistent with the literature report[8].

R*f* = 0.40 (DCM/MeOH = 20:1).

^{1}H-NMR (400 MHz, DMSO) δ 11.80 (d, J = 5.1 Hz, 1H), 8.21 (d, J = 7.2 Hz, 1H), 6.12 (td, J = 6.7, 2.0 Hz, 1H), 5.24 (brs, 1H), 5.14 (brs, 1H), 4.23 (h, J = 3.0 Hz, 1H), 3.78 (q, J = 3.4 Hz, 1H), 3.60 (td, J = 11.1, 10.3, 3.5 Hz, 2H), 2.10 (dd, J = 6.7, 4.7 Hz, 3H).

^{13}C NMR (101 MHz, DMSO) δ 157.50 (d, J = 26.2 Hz), 157.37, 149.45, 140.40 (d, J = 229.7 Hz), 125.20 (d, J = 34.3 Hz), 87.95, 85.00, 70.59, 61.47, 40.25.

HRMS (ESI-TOF) *m/z*: Calcd for $C_9H_{12}N_2O_5F^+$ $[M+H]^+$: 247.0725, found: 247.0770.

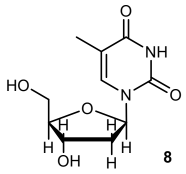

8

5-Methyl-2’-deoxyluridine (8): 5-Methyl-2'-deoxycytidine (CAS No. 838-07-3) was used as the starting material. Prepared according to the standard reaction condition for cytidine analogs in 88% isolated yield (21.2 mg) as a white powder. NMR data is consistent with the literature report[9].

R*f* = 0.40 (DCM/MeOH = 20:1).

^{1}H-NMR (400 MHz, DMSO) δ 11.26 (s, 1H), 7.69 (q, J = 1.3 Hz, 1H), 6.16 (dd, J = 7.6, 6.2 Hz, 1H), 5.22 (brs, 1H), 5.01 (brs, 1H), 4.23 (dt, J = 5.8, 3.2 Hz, 1H), 3.75 (dd, J = 4.0, 2.9 Hz, 1H), 3.56 (qd, J = 11.8, 3.9 Hz, 1H), 2.12 – 1.94 (m, 2H), 1.77 (d, J = 1.3 Hz, 3H).

^{13}C NMR (101 MHz, DMSO) δ 164.21, 150.93, 136.58, 109.83, 87.70, 84.20, 70.90, 61.80, 39.87, 12.72.

HRMS (ESI-TOF) *m/z*: Calcd for $C_{10}H_{15}N_2O_5^+$ $[M+H]^+$: 243.0975, found: 243.0966.

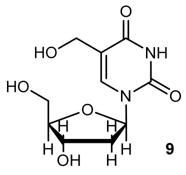

9

5-Hydroxymethyl-2’-deoxyluridine (9): 5-Hydroxymethyl-2'-deoxycytidine (CAS No. 7226-77-9) was used as the starting material. Prepared according to the standard reaction condition for cytidine analogs in 82% isolated yield (21.1 mg) as a white powder. NMR data is consistent with the literature report[10].

R*f* = 0.20 (DCM/MeOH = 20:1).

^{1}H-NMR (400 MHz, DMSO) δ 11.31 (s, 1H), 7.72 (d, J = 1.2 Hz, 1H), 6.18 (t, J = 6.9 Hz, 1H), 5.25 (d, J = 4.2 Hz, 1H), 4.97 (t, J = 5.1 Hz, 1H), 4.90 (t, J = 5.5 Hz, 1H), 4.27 – 4.19 (m, 1H),

4.13 (dd, J = 5.5, 1.1 Hz, 2H), 3.77 (dd, J = 4.2, 2.8 Hz, 1H), 3.61 – 3.51 (m, 2H), 2.14 – 2.01 (m, 2H).

^{13}C NMR (101 MHz, DMSO) δ 163.11, 150.83, 137.23, 114.72, 87.75, 84.37, 71.01, 61.91, 56.47, 39.91.

HRMS (ESI-TOF) *m/z*: Calcd for $C_{10}H_{15}N_2O_6^+$ $[M+H]^+$: 259.0925, found: 259.0922.

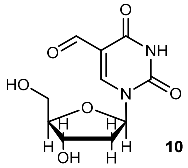

10

5-formyl-2'-deoxyuridine (10): 2'-Deoxy-5-formylcytidine (CAS No. 137017-45-9) was used as the starting material. Prepared according to the standard reaction condition for cytidine analogs in 79% isolated yield (18.5 mg) as a white powder. NMR data is consistent with the literature report[11].

R*f* = 0.20 (DCM/MeOH = 20:1).

^{1}H-NMR (400 MHz, DMSO) δ 11.29 (s, 1H), 9.31 (s, 1H), 8.26 (s, 1H), 5.64 (t, J = 6.3 Hz, 1H), 4.82 (s, 1H), 4.66 (s, 1H), 3.79 (s, 1H), 3.41 (q, J = 3.4 Hz, 1H), 3.22 – 3.11 (m, 2H), 1.84 – 1.68 (m, 2H).

^{13}C NMR (101 MHz, DMSO) δ 186.64, 162.20, 150.05, 147.58, 111.15, 88.42, 86.34, 70.30, 61.17, 41.1

HRMS (ESI-TOF) *m/z*: Calcd for $C_{10}H_{13}N_2O_6^+$ $[M+H]^+$: 257.0768, found: 257.0764.

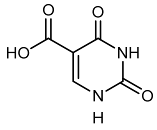

11

5-Carboxyuracil (11): 5-Carboxycytosine (CAS No. 3650-93-9) was used as the starting material. Prepared according to the standard reaction condition for cytidine analogs in 46% isolated yield (7.2 mg) as a white powder. NMR data is consistent with the literature report[12].

R*f* = 0.3 (DCM/MeOH = 10:1).

^{1}H-NMR (400 MHz, DMSO) δ 12.72 (brs, 1H), 12.00 – 11.95 (m, 2H), 8.25 (s, 1H).

^{13}C NMR (101 MHz, DMSO) δ 165.67, 163.95, 150.82, 150.63, 101.70.

HRMS (ESI-TOF) *m/z*: Calcd for $C_5H_5N_2O_4^+$ $[M+H]^+$: 157.0244, found: 157.0248.

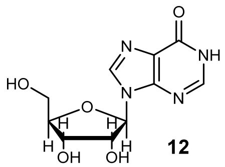

12

Inosine (12): Adenosine (CAS No. 58-61-7) was used as the starting material. Prepared according to the standard reaction condition for adenosine analogs in 86% isolated yield (23.1 mg) as a white powder. NMR data is consistent with the literature report[13].

R*f* = 0.40 (DCM/MeOH = 10:1).

^{1}H-NMR (400 MHz, DMSO) δ 12.39 (s, 1H), 8.34 (s, 1H), 8.08 (s, 1H), 5.87 (d, J = 5.8 Hz, 1H), 5.52 – 5.46 (m, 1H), 5.21 (s, 1H), 5.08 (s, 1H), 4.53 – 4.45 (m, 1H), 4.13 (t, J = 4.4 Hz, 1H), 3.94 (q, J = 3.9 Hz, 1H), 3.66 (dd, J = 12.0, 4.1 Hz, 1H), 3.55 (dd, J = 12.0, 4.1 Hz, 1H).

^{13}C NMR (101 MHz, DMSO) δ 157.04, 148.68, 146.38, 139.23, 124.88, 87.91, 86.08, 74.57, 70.77, 61.75.

HRMS (ESI-TOF) *m/z*: Calcd for $C_{10}H_{13}N_4O_5^+$ $[M+H]^+$: 269.0880, found: 269.0870.

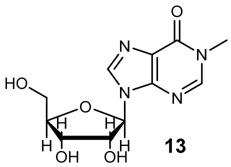

1-Methylinosine (13): 1-Methyl adenosine (CAS No. 15763-06-1) was used as the starting material. Prepared according to the standard reaction condition for adenosine analogs in 93% isolated yield (26.2 mg) as a white powder. NMR data is consistent with the literature report[4].

R*f* = 0.70 (DCM/MeOH = 10:1).

^{1}H-NMR (400 MHz, DMSO) δ 8.41 (s, 1H), 8.34 (s, 1H), 5.86 (d, J = 5.8 Hz, 1H), 5.49 (d, J = 6.2 Hz, 1H), 5.22 (d, J = 5.0 Hz, 1H), 5.11 – 5.04 (m, 1H), 4.17 – 4.11 (m, 1H), 3.65 (ddd, J = 12.0, 5.2, 4.0 Hz, 1H), 3.58 – 3.52 (m, 1H), 3.51 (s, 3H).

^{13}C NMR (101 MHz, DMSO) δ 156.44, 148.82, 147.61, 139.23, 123.64, 87.41, 85.67, 74.09, 70.33, 61.32, 33.57.

HRMS (ESI-TOF) *m/z*: Calcd for $C_{11}H_{15}N_4O_5^+$ $[M+H]^+$: 283.1037, found: 283.1020.

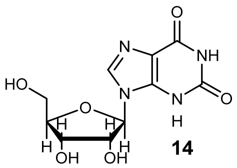

Xanthosine (14): Prepared according to the standard reaction condition for adenosine analogs with 2-aminoadenosine (CAS: 2096-10-8) as starting material in 8% isolated yield. Prepared according to the standard reaction condition for guanosine analogs with 2-aminoadenosine (CAS: 2096-10-8) as starting material, yielding 44% (12.5 mg). Prepared according to the standard reaction condition for adenosine analogs with isoguanosine (CAS: 3373-53-3) as starting material, yielding 77% (21.9 mg) as white powder. NMR data is consistent with the literature report[14].

R*f* = 0.8 (H_2O/MeOH = 20:1), reversed phase TLC; 0.2 (DCM/MeOH = 1:1), silica gel TLC

^{1}H-NMR (400 MHz, DMSO) δ 11.73 (s, 1H), 10.88 (s, 1H), 7.87 (s, 1H), 6.09 (s, 0H), 5.74 (d, J = 6.8 Hz, 1H), 5.49 (d, J = 6.4 Hz, 1H), 5.31 – 5.25 (m, 2H), 4.22 (q, J = 5.5 Hz, 1H), 4.06 (d, J = 5.4 Hz, 1H), 4.01 (q, J = 2.5 Hz, 1H), 3.66 (d, J = 2.7 Hz, 2H).

^{13}C NMR (101 MHz, DMSO) δ 158.36, 150.93, 139.81, 136.15, 116.76, 89.18, 86.57, 74.42, 71.34, 61.74.

HRMS (ESI-TOF) *m/z*: Calcd for $C_{10}H_{13}N_4O_6^+$ $[M+H]^+$: 285.0830, found: 285.0850.

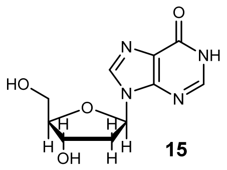

15

2′-Deoxyinosine (15): 2'-Deoxyadenosine (CAS No. 16373-93-6) was used as the starting material. Prepared according to the standard reaction condition for adenosine analogs in 82% isolated yield (20.3 mg) as a white powder. NMR data is consistent with the literature report[15].

R*f* = 0.5 (DCM/MeOH = 8:1).

^{1}H-NMR (400 MHz, DMSO) δ 12.39 (s, 1H), 8.34 (s, 1H), 8.08 (s, 1H), 5.87 (d, J = 5.8 Hz, 1H), 5.52 – 5.46 (m, 1H), 5.21 (s, 1H), 5.08 (s, 1H), 4.53 – 4.45 (m, 1H), 4.13 (t, J = 4.4 Hz, 1H), 3.94 (q, J = 3.9 Hz, 1H), 3.66 (dd, J = 12.0, 4.1 Hz, 1H), 3.55 (dd, J = 12.0, 4.1 Hz, 1H).

^{13}C NMR (101 MHz, DMSO) δ 157.04, 148.68, 146.38, 139.23, 124.88, 87.91, 86.08, 74.57, 70.77, 61.75.

HRMS (ESI-TOF) *m/z*: Calcd for $C_{10}H_{13}N_4O_4^+$ $[M+H]^+$: 253.0931, found: 253.0909.

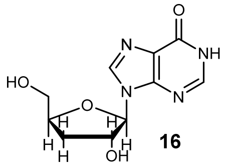

16

3'-deoxyinosine (16): Cordycepin (CAS No. 73-03-0) was used as the starting material. Prepared according to the standard reaction condition for adenosine analogs in 86% isolated yield (20.3 mg) as a white powder. NMR data is consistent with the literature report[15].

R*f* = 0.5 (DCM/MeOH = 8:1).

^{1}H-NMR (400 MHz, DMSO) δ 12.36 (s, 1H), 8.34 (s, 1H), 8.07 (s, 1H), 5.87 (d, J = 2.1 Hz, 1H), 5.70 (s, 1H), 5.04 (s, 1H), 4.51 (dt, J = 5.3, 2.5 Hz, 1H), 4.36 (td, J = 5.9, 2.6 Hz, 1H), 3.70 (dd, J = 11.9, 3.4 Hz, 1H), 2.21 (ddd, J = 13.1, 9.3, 5.6 Hz, 1H), 1.90 (ddd, J = 13.2, 6.2, 2.7 Hz, 1H).

^{13}C NMR (101 MHz, DMSO) δ 157.10, 148.16, 146.22, 138.65, 124.75, 91.22, 81.55, 75.67, 62.72, 34.30.

HRMS (ESI-TOF) *m/z*: Calcd for $C_{10}H_{13}N_4O_4^+$ $[M+H]^+$: 253.0931, found: 253.0911.

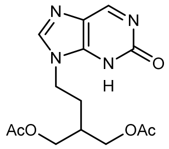

17

9-(4-Acetoxy-3-acetoxymethylbutyl)- 2-hydroxypurine (17): Famciclovir (CAS No. 104227-87-4) was used as the starting material. Prepared according to the standard reaction condition for adenosine analogs in 77% isolated yield (24.5 mg) as a yellow powder. NMR data is consistent with the literature report.

R*f* = 0.4 (DCM/MeOH = 25:1).

^{1}H-NMR (400 MHz, DMSO) δ 11.86 (s, 1H), 8.41 (s, 1H), 8.23 (s, 1H), 4.08 – 3.96 (m, 6H), 2.00 (s, 6H), 1.96 – 1.92 (m, 1H), 1.83 (q, J = 6.7 Hz, 2H).

^{13}C NMR (101 MHz, DMSO) δ 170.37, 159.36, 156.04, 147.62, 138.59, 123.11, 63.44, 40.03, 34.43, 27.50, 20.61.

HRMS (ESI-TOF) *m/z*: Calcd for $C_{14}H_{19}N_4O_5^+$ $[M+H]^+$: 323.1350, found: 323.1363.

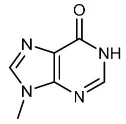
18

9-Methylhypoxanthine (18): 3-Methyladenine (CAS No. 700-00-5) was used as the starting material. Prepared according to the standard reaction condition for adenosine analogs in 85% isolated yield (12.9 mg) as a white powder. NMR data is consistent with the literature report[16].

R$_f$ = 0.5 (DCM/MeOH = 20:1).

^{1}H-NMR (400 MHz, DMSO) δ 12.25 (s, 1H), 8.03 (s, 1H), 8.03 (s, 1H), 3.72 (s, 3H).

^{13}C NMR (101 MHz, DMSO) δ 158.73, 148.99, 147.04, 140.06, 123.88, 29.59.

HRMS (ESI-TOF) *m/z*: Calcd for $C_6H_7N_4O^+$ $[M+H]^+$: 151.0614, found: 151.0612.

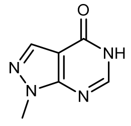
19

1-Methyl-1,5-dihydro-pyrazolo[3,4-d]pyrimidin-4-one (19): 1-Methyl-1H-pyrazolo[3,4-d]pyrimidin-4-ylamine (CAS No. 5334-99-6) was used as the starting material. Prepared according to the standard reaction condition for adenosine analogs in 88% isolated yield (13.2 mg) as a white powder. NMR data is consistent with the literature report[17].

R$_f$ = 0.5 (DCM/MeOH = 20:1).

^{1}H-NMR (400 MHz, DMSO) δ 12.15 (s, 1H), 8.07 (d, J = 3.9 Hz, 1H), 8.04 (s, 1H), 3.90 (s, 3H).

^{13}C NMR (101 MHz, DMSO) δ 157.73, 152.26, 148.33, 134.53, 106.14, 34.49.

HRMS (ESI-TOF) *m/z*: Calcd for $C_6H_7N_4O^+$ $[M+H]^+$: 151.0614, found: 151.0619.

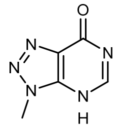
20

3-Methyl-3H-[1,2,3]triazolo[4,5-d]pyrimidine-7(6H)-one (20): 6-Amino-9-methyl-8-azapurin (CAS No. 6312-55-6) was used as the starting material. Prepared according to the standard reaction condition for adenosine analogs in 92% isolated yield (13.8 mg) as a yellow powder.

R$_f$ = 0.5 (DCM/MeOH = 25:1).

^{1}H-NMR (400 MHz, DMSO) δ 12.66 (s, 1H), 8.24 (d, J = 3.9 Hz, 1H), 4.12 (s, 3H).

^{13}C NMR (101 MHz, DMSO) δ 155.37, 149.52, 148.72, 129.46, 32.98.

HRMS (ESI-TOF) *m/z*: Calcd for $C_5H_6N_5O^+$ $[M+H]^+$: 152.0567, found: 152.0588.

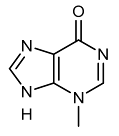

21

3-Methylhypoxanthine (21): 3-Methyladenine (CAS No. 5142-23-4) was used as the starting material. Prepared according to the standard reaction condition for adenosine analogs in 87% isolated yield (13.1 mg) as a white powder. NMR data is consistent with the literature report[18].

R*f* = 0.4 (DCM/7N NH_3 in MeOH = 10:1).

^{1}H-NMR (400 MHz, DMSO) δ 8.29 (s, 1H), 7.76 (s, 1H), 3.89 (s, 3H).

^{13}C NMR (101 MHz, DMSO) δ 155.42, 152.88, 150.81, 144.21, 120.74, 36.26.

HRMS (ESI-TOF) *m/z*: Calcd for $C_6H_7N_4O^+$ $[M+H]^+$: 151.0614, found: 151.0617.

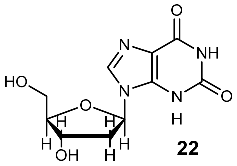

22

2'-Deoxyxanthosine (22): 2’-Deoxylguanosine (CAS No. 312693-72-4) was used as the starting material. Prepared according to the standard reaction condition for guanosine analogs in 47% isolated yield (12.5 mg) as a white powder. NMR data is consistent with the literature report[19].

R*f* = 0.4 (DCM/7N NH_3 in MeOH = 10:1).

^{1}H-NMR (400 MHz, DMSO) δ 10.71 (s, 1H), 7.87 (s, 1H), 6.18 (dd, J = 8.0, 5.9 Hz, 1H), 5.33 (d, J = 3.7 Hz, 1H), 4.34 (dd, J = 5.7, 2.8 Hz, 1H), 3.90 (q, J = 3.0 Hz, 1H), 3.67 – 3.56 (m, 2H), 2.22 (ddd, J = 13.3, 6.0, 2.7 Hz, 1H).

^{13}C NMR (101 MHz, DMSO) δ 158.50, 151.52, 140.73, 135.47, 116.54, 88.36, 85.62, 71.36, 61.92, 40.73.

HRMS (ESI-TOF) *m/z*: Calcd for $C_{10}H_{13}N_4O_5^+$ $[M+H]^+$: 269.0880, found: 269.0891.

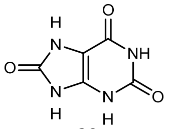

23

Uric acid (23): 8-Hydroxyguanine (CAS No. 5614-64-2) was used as the starting material. Prepared according to the standard reaction condition for guanosine analogs in 47% isolated yield (7.9 mg) as a white powder.

R*f* = 0.7 (H_2O/MeOH = 20:1), reversed phase TLC; 0.2 (DCM/MeOH = 8:1), silica gel TLC

^{1}H-NMR (400 MHz, DMSO) δ 11.55 (s, 2H), 10.69 (s, 1H), 10.53 (s, 1H).

^{13}C NMR (101 MHz, DMSO) δ 153.89, 152.81, 150.66, 137.17, 97.63.

HRMS (ESI-TOF) *m/z*: Calcd for $C_5H_5N_4O_3^+$ $[M+H]^+$: 169.0356, found: 169.1082.

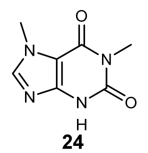

24

Paraxanthine (24): 2-Amino-1,7-dimethyl-6,7-dihydro-1H-purin-6-one (CAS No. 26758-00-9) was used as the starting material. Prepared according to the standard reaction condition for guanosine analogs in 54% isolated yield (9.7 mg) as a white powder. NMR data is consistent with the literature report[20].

R*f* = 0.6 (DCM/MeOH = 10:1)

^{1}H-NMR (400 MHz, DMSO) δ 11.82 (s, 0H), 7.91 (d, J = 0.7 Hz, 1H), 3.87 – 3.82 (m, 3H), 3.17 (s, 3H).

^{13}C NMR (101 MHz, DMSO) δ 155.84, 151.60, 147.84, 143.45, 106.98, 33.40, 27.21.

HRMS (ESI-TOF) *m/z*: Calcd for $C_7H_9N_4O_2^+$ [M+H]$^+$: 181.0720, found: 181.0700.

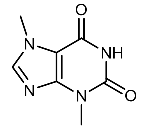

25

Theobromine (25): 2-Amino-3,7-dimethyl-3H-purin-6(7H)-one (CAS No. 19143-67-0) was used as the starting material. Prepared according to the standard reaction condition for guanosine analogs in 57% isolated yield (10.2 mg) as a white powder. NMR data is consistent with the literature report[21].

R*f* = 0.6 (DCM/MeOH = 15:1)

^{1}H-NMR (400 MHz, DMSO) δ 11.10 (s, 0H), 7.97 (d, J = 0.7 Hz, 1H), 3.85 (s, 2H), 3.33 (s, 3H).

^{13}C NMR (101 MHz, DMSO) δ 154.98, 151.03, 149.82, 142.78, 107.10, 33.08, 28.41.

HRMS (ESI-TOF) *m/z*: Calcd for $C_7H_9N_4O_2^+$ [M+H]$^+$: 181.0720, found: 181.0731.

D. Synthesis of kethoxal

The synthesis of both kethoxal and N_3-kethoxal followed a previously established protocol[22]. We tested the reactivity of both compounds with guanosine and found that they exhibit the exact same reactivity. However, N_3-kethoxal offers additional potential for use in DBCO-bead purification, which could greatly simplify subsequent purification and deprotection steps. Thus, we used either of these as the protective reagent.

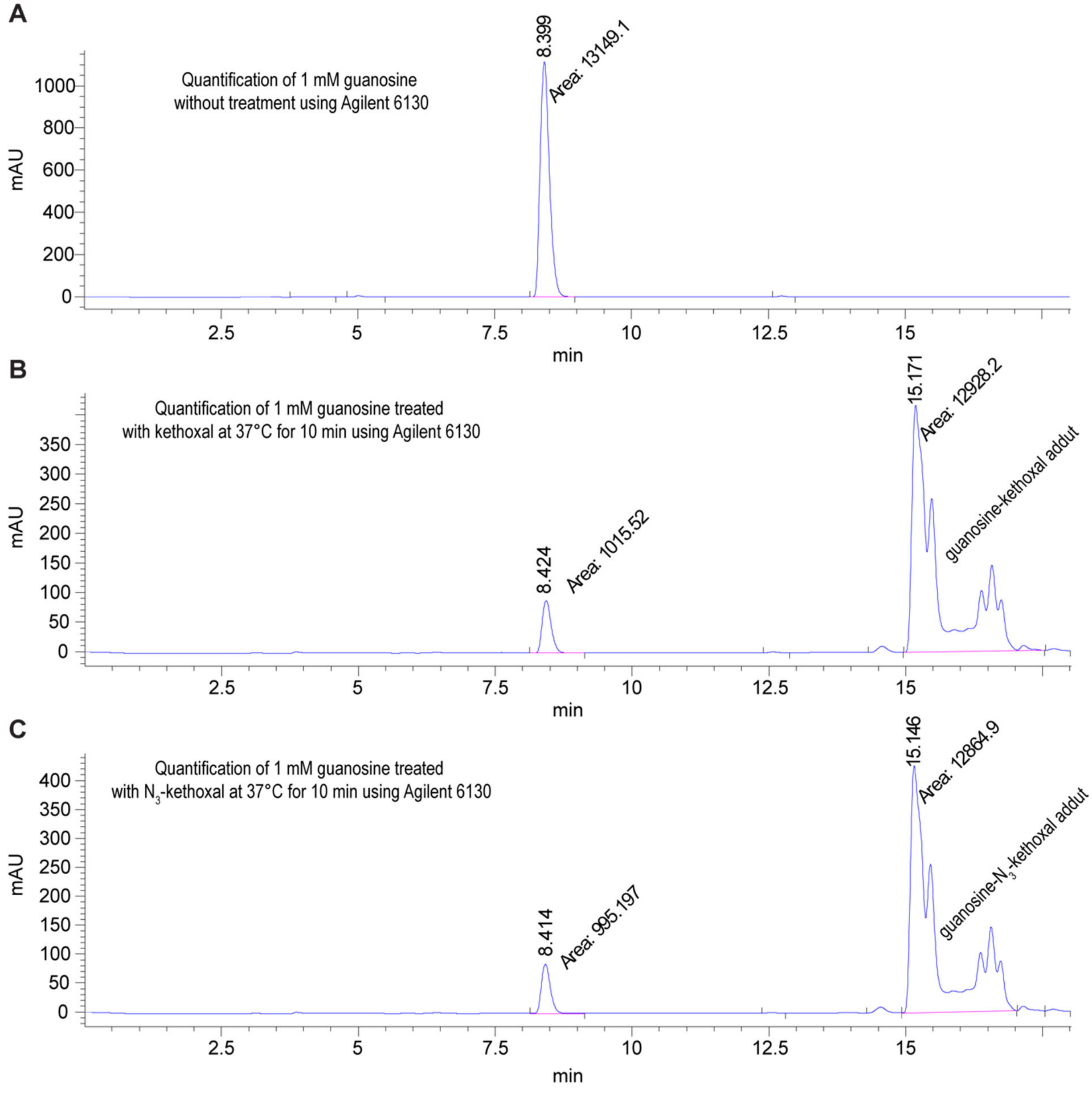

Note Figure 1 | LC-MS spectrum for kethoxal and N_3-kethoxal activity test.

E. NMR Spectra

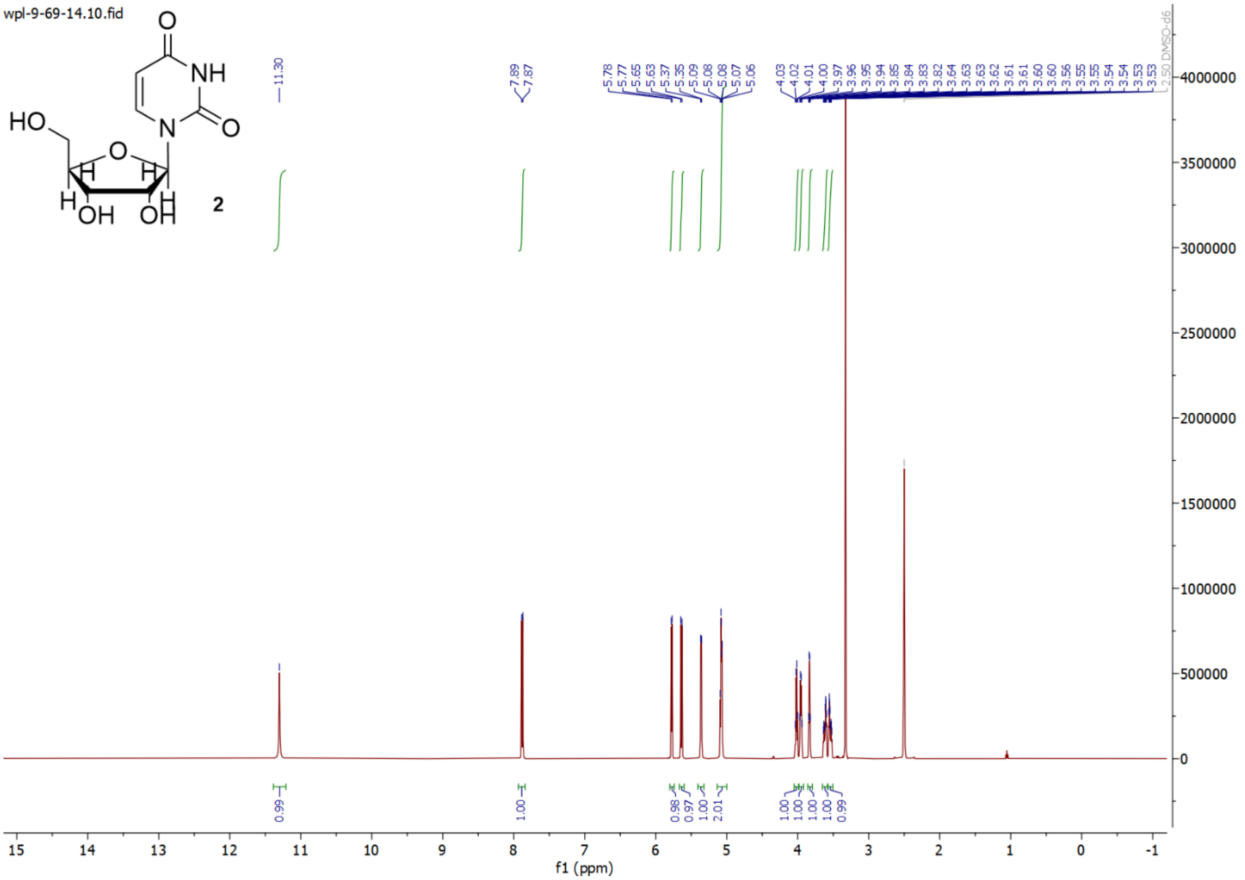

Note Figure 2 | ^{1}H NMR spectrum of product 2.

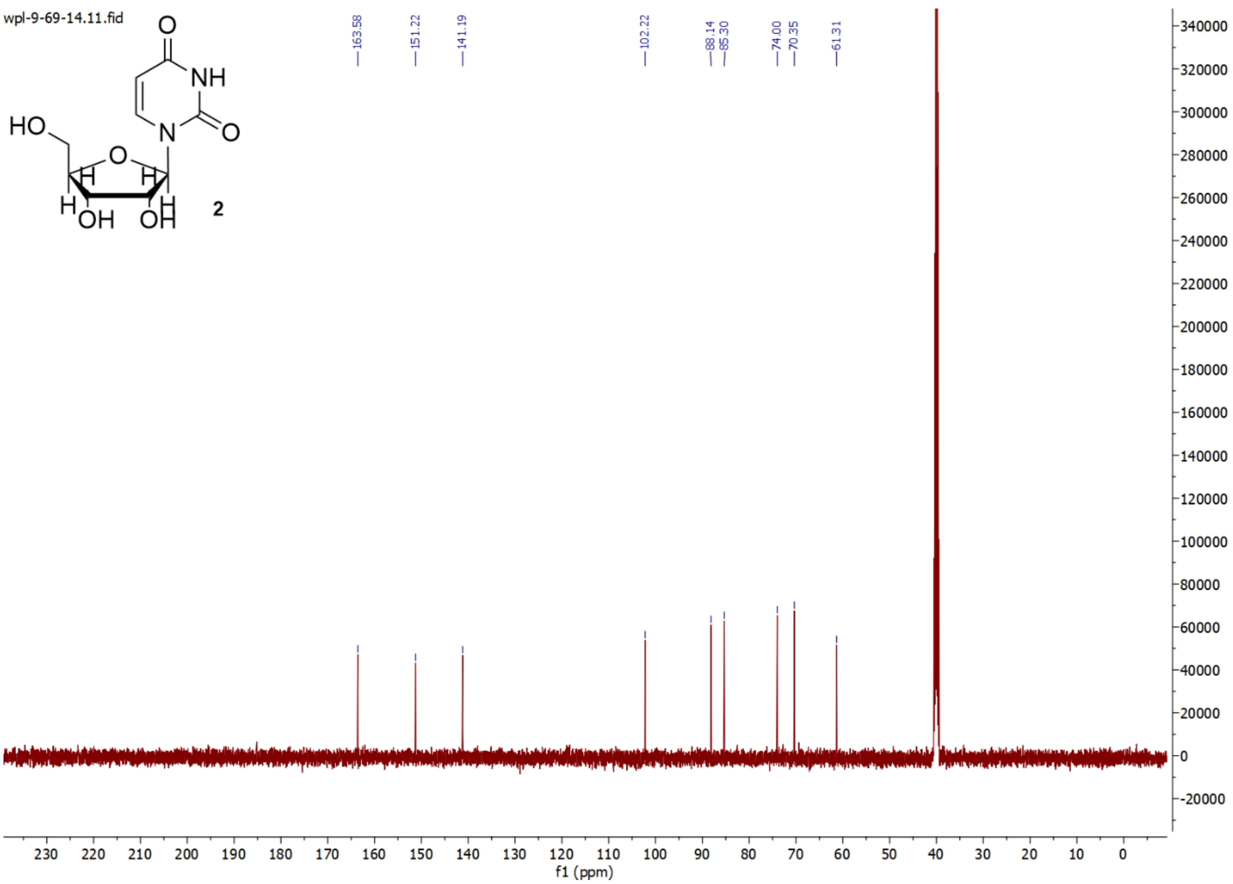

Note Figure 3 | ^{13}C NMR spectrum of product 2.

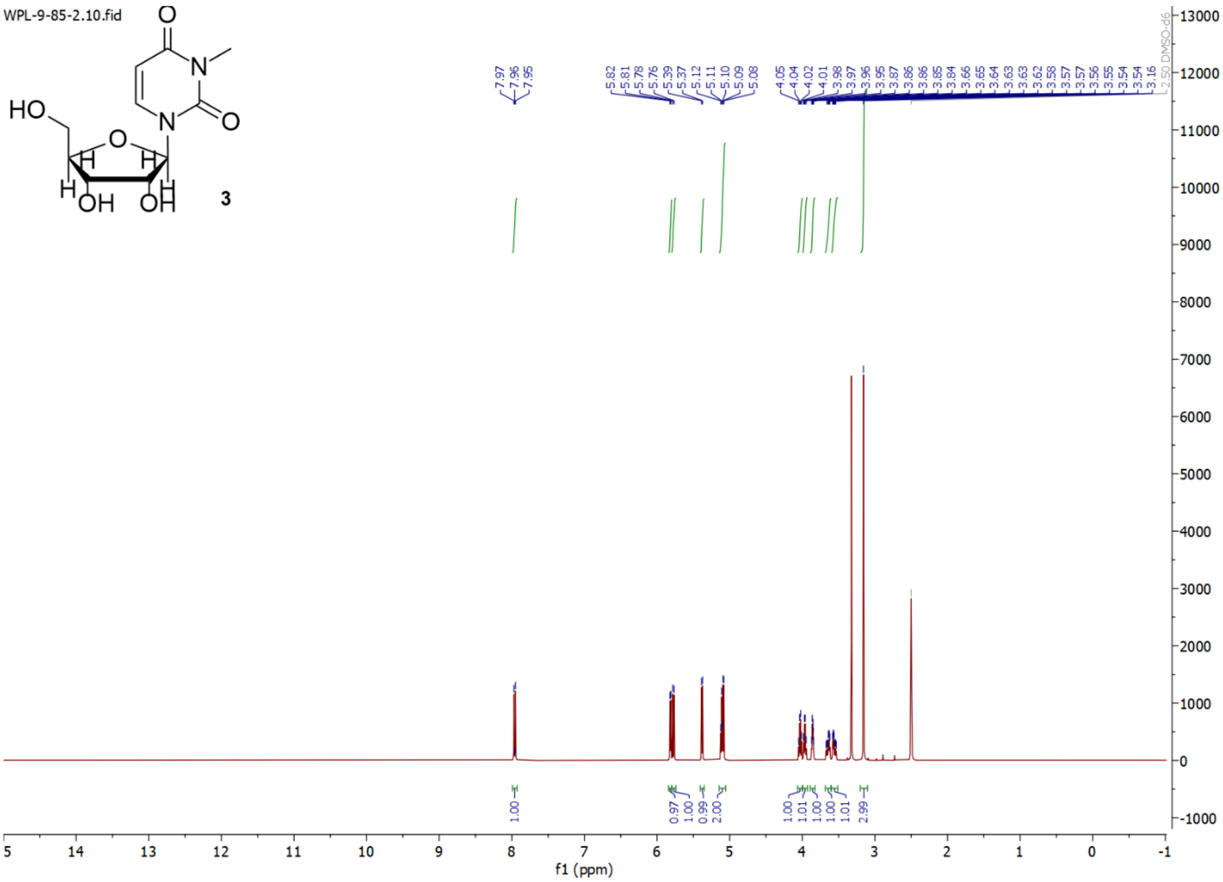

Note Figure 4 | ¹H NMR spectrum of product 3.

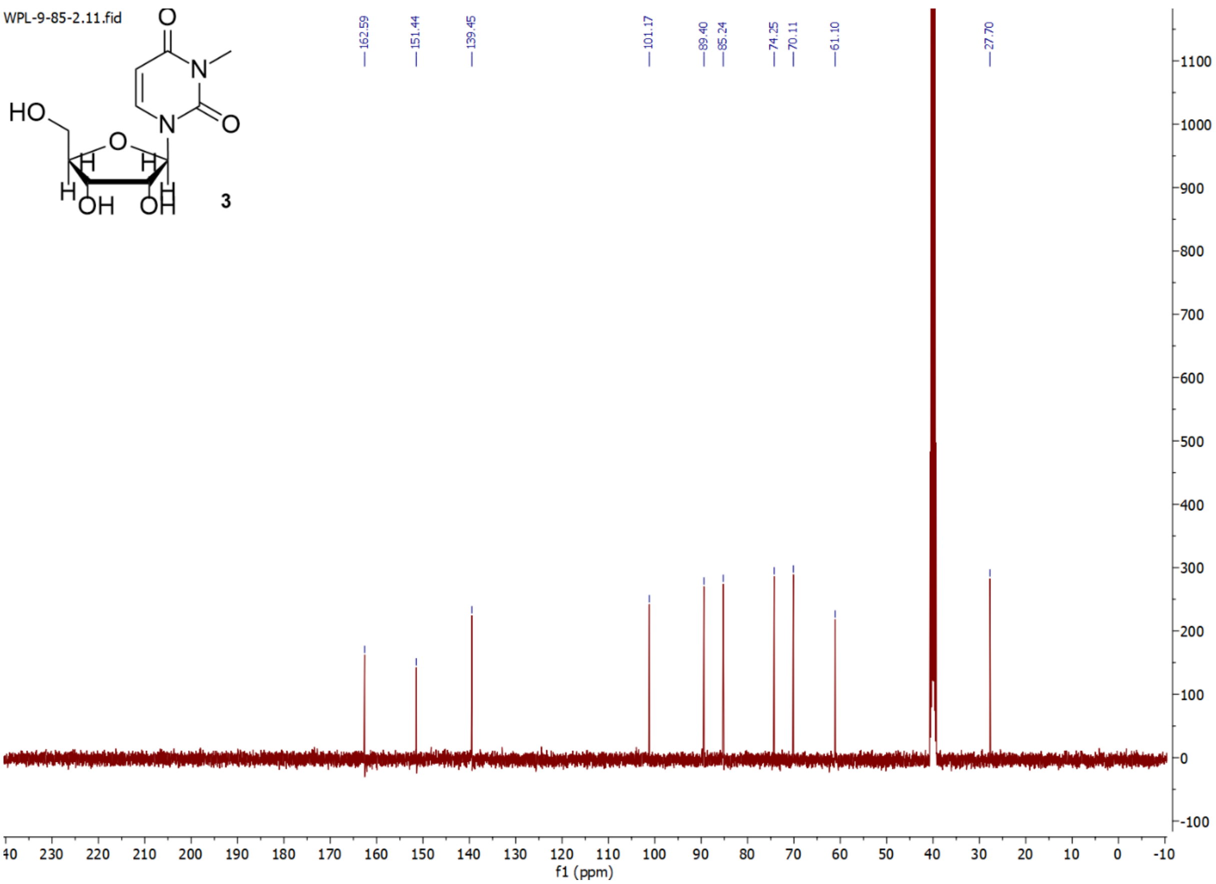

Note Figure 5 | ^{13}C NMR spectrum of product 3.

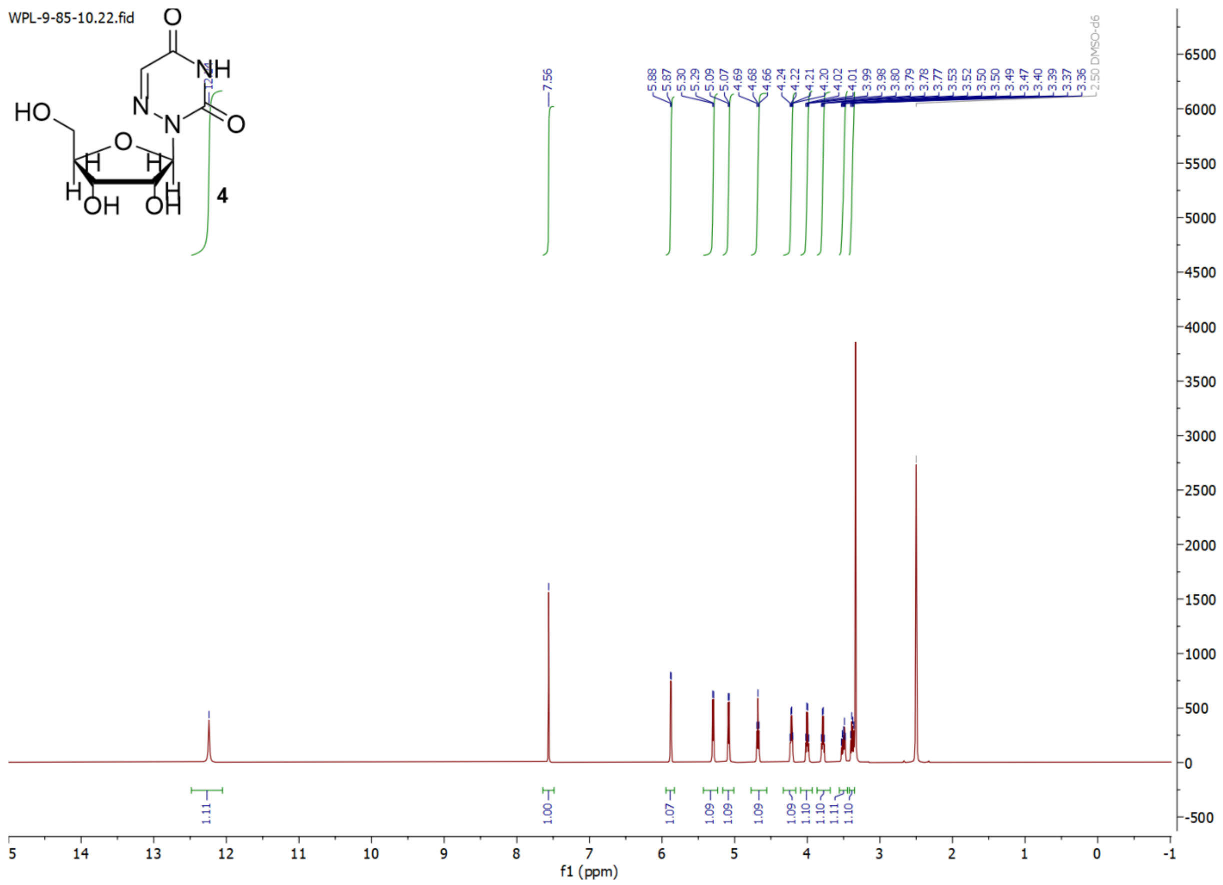

Note Figure 6 | ^{1}H NMR spectrum of product 4.

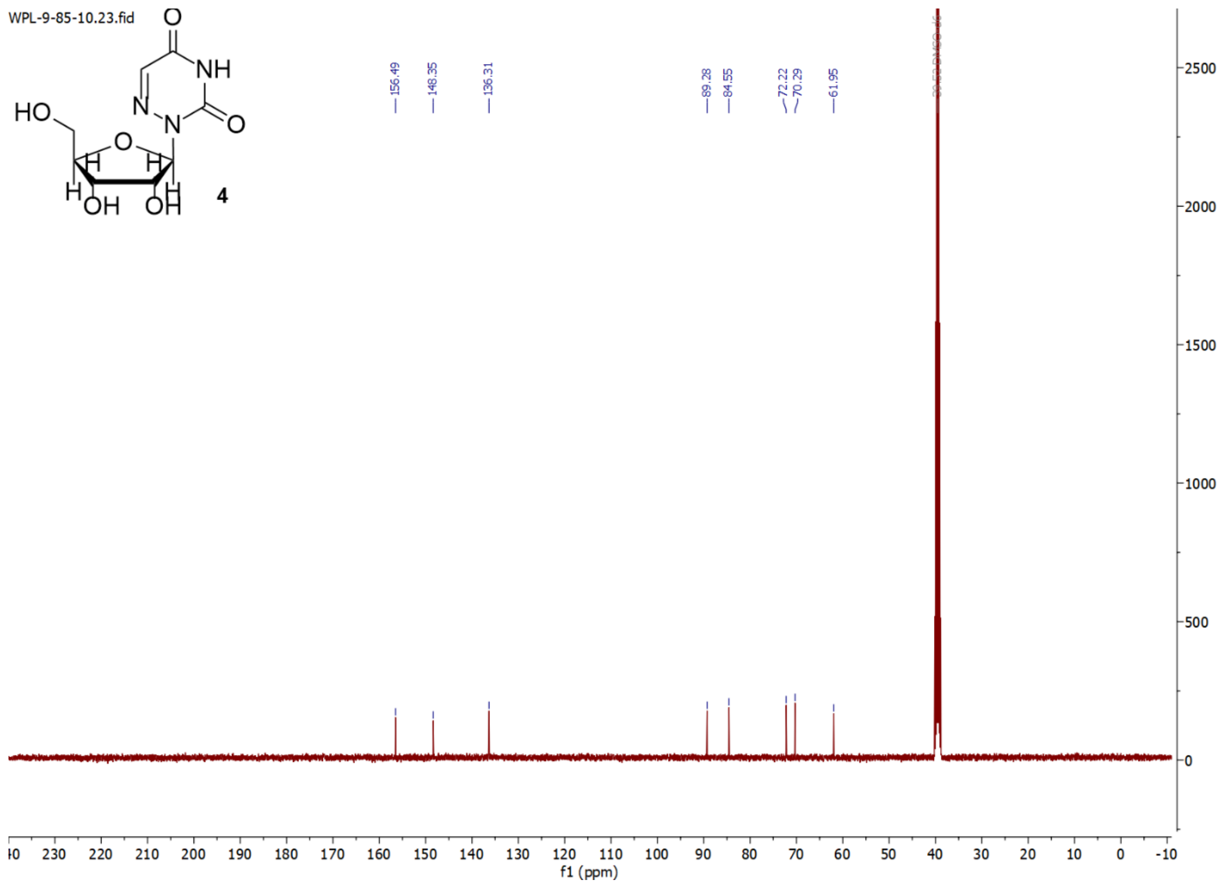

Note Figure 7 | ^{13}C NMR spectrum of product 4.

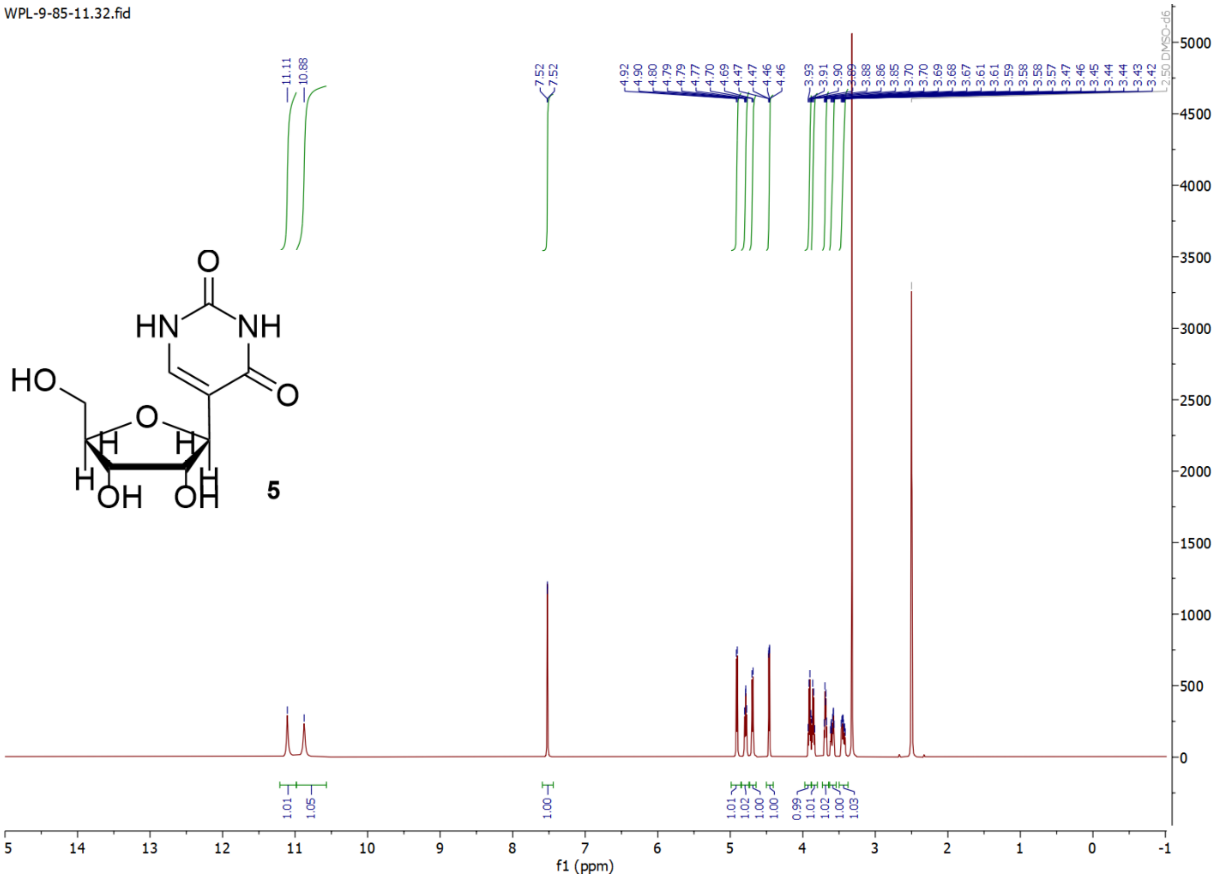

Note Figure 8 | ^{1}H NMR spectrum of product 5.

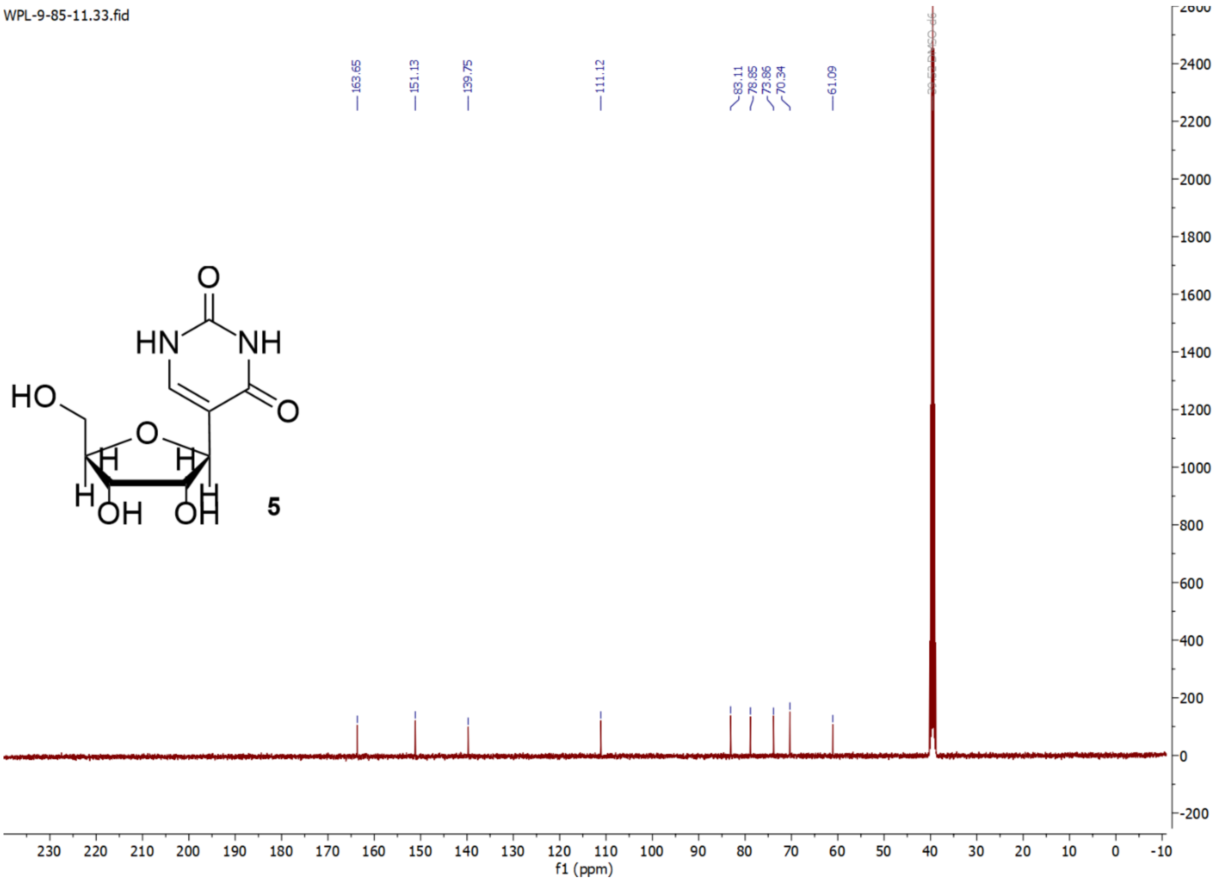

Note Figure 9 | ^{13}C NMR spectrum of product 5.

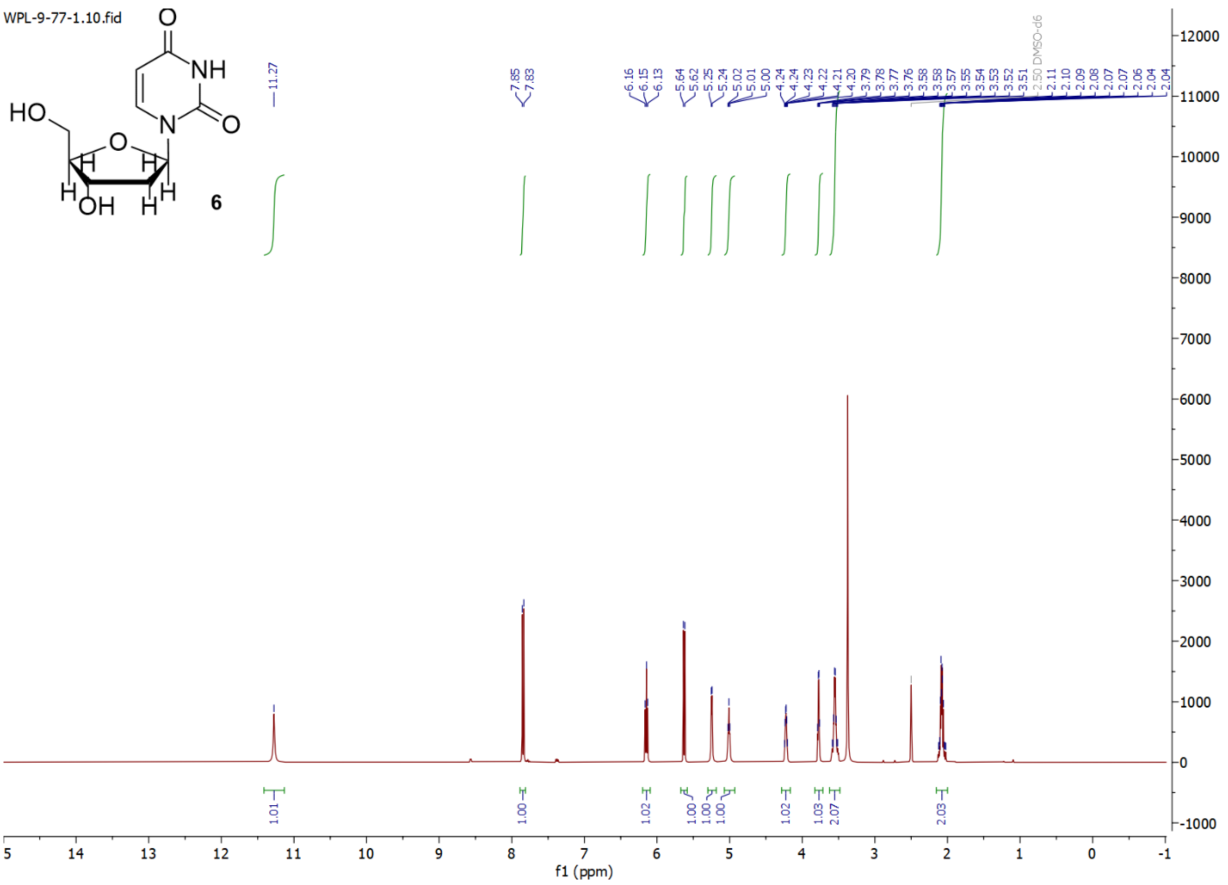

Note Figure 10 | ^{1}H NMR spectrum of product 6.

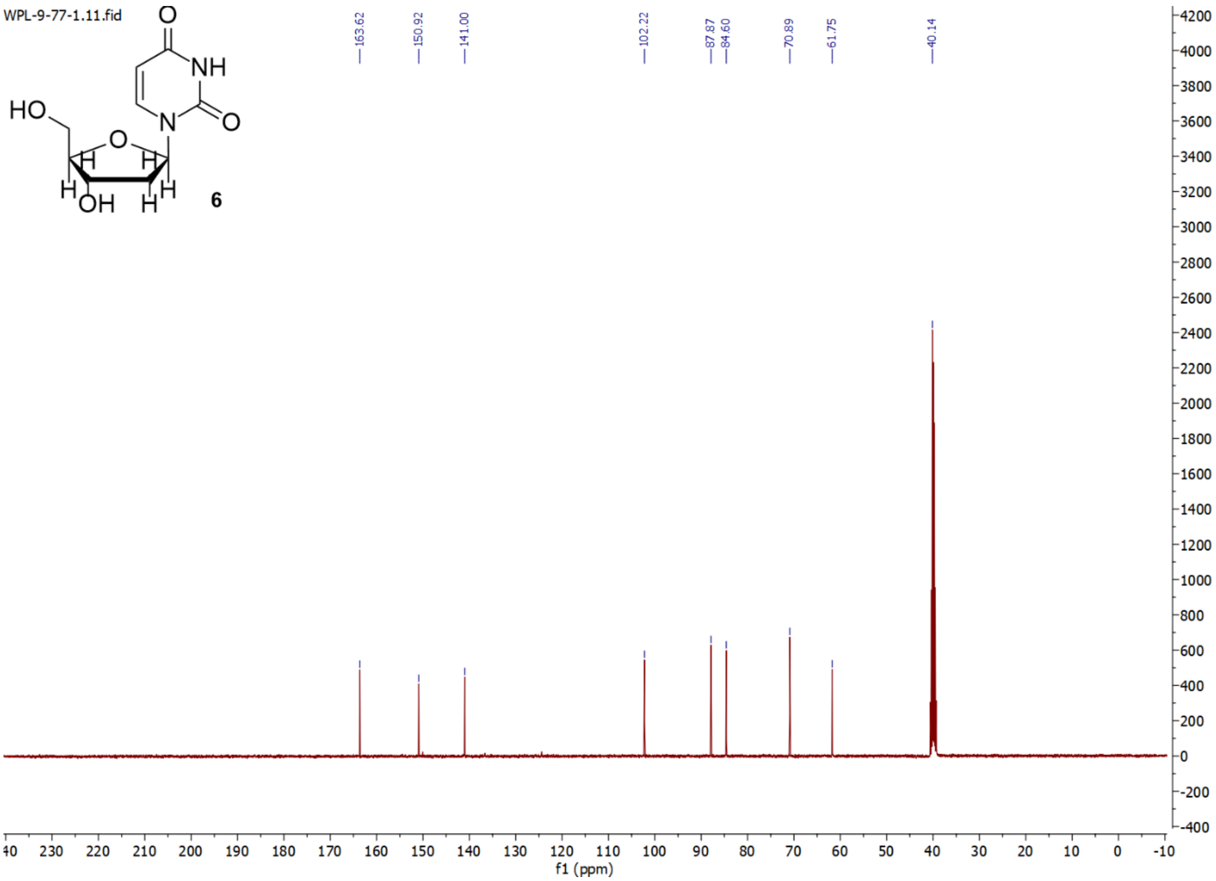

Note Figure 11 | ^{13}C NMR spectrum of product 6.

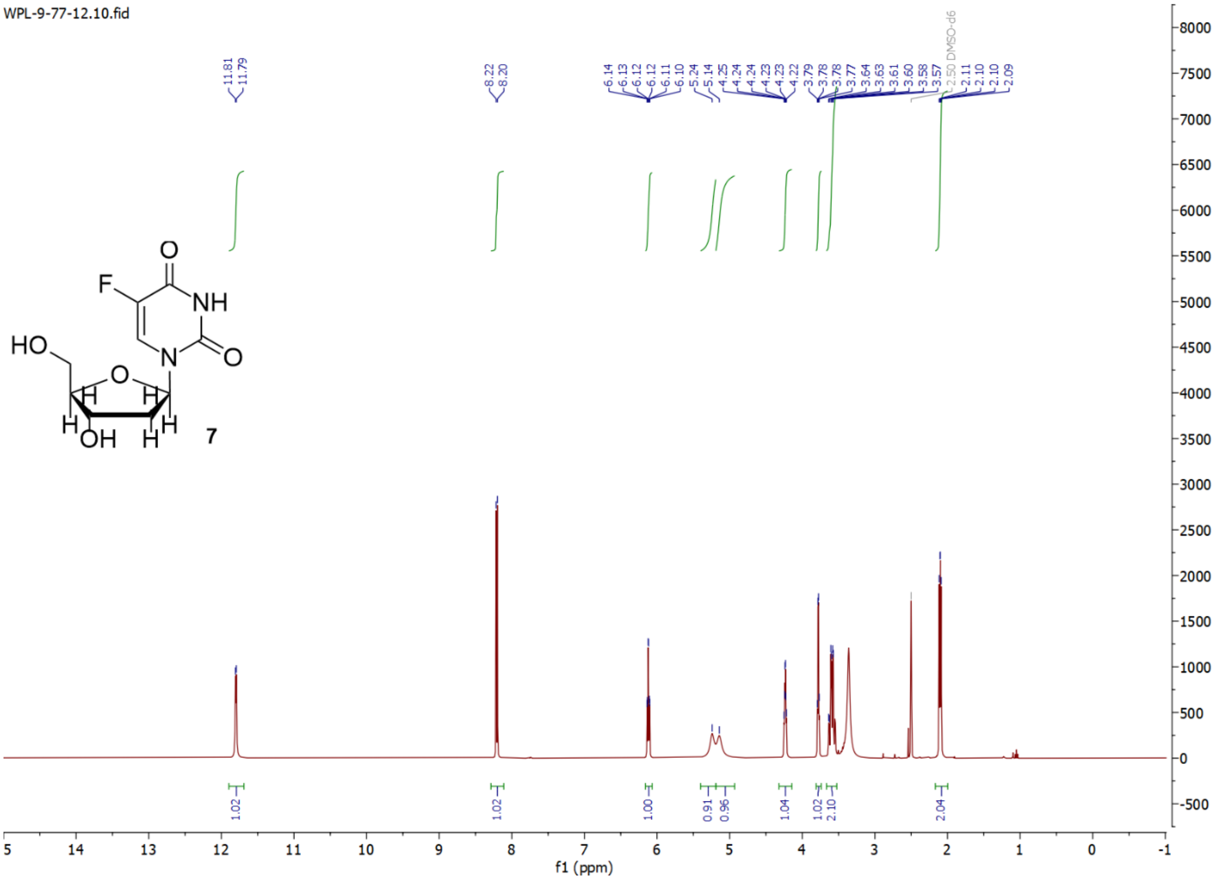

Note Figure 12 | ^{1}H NMR spectrum of product 7.

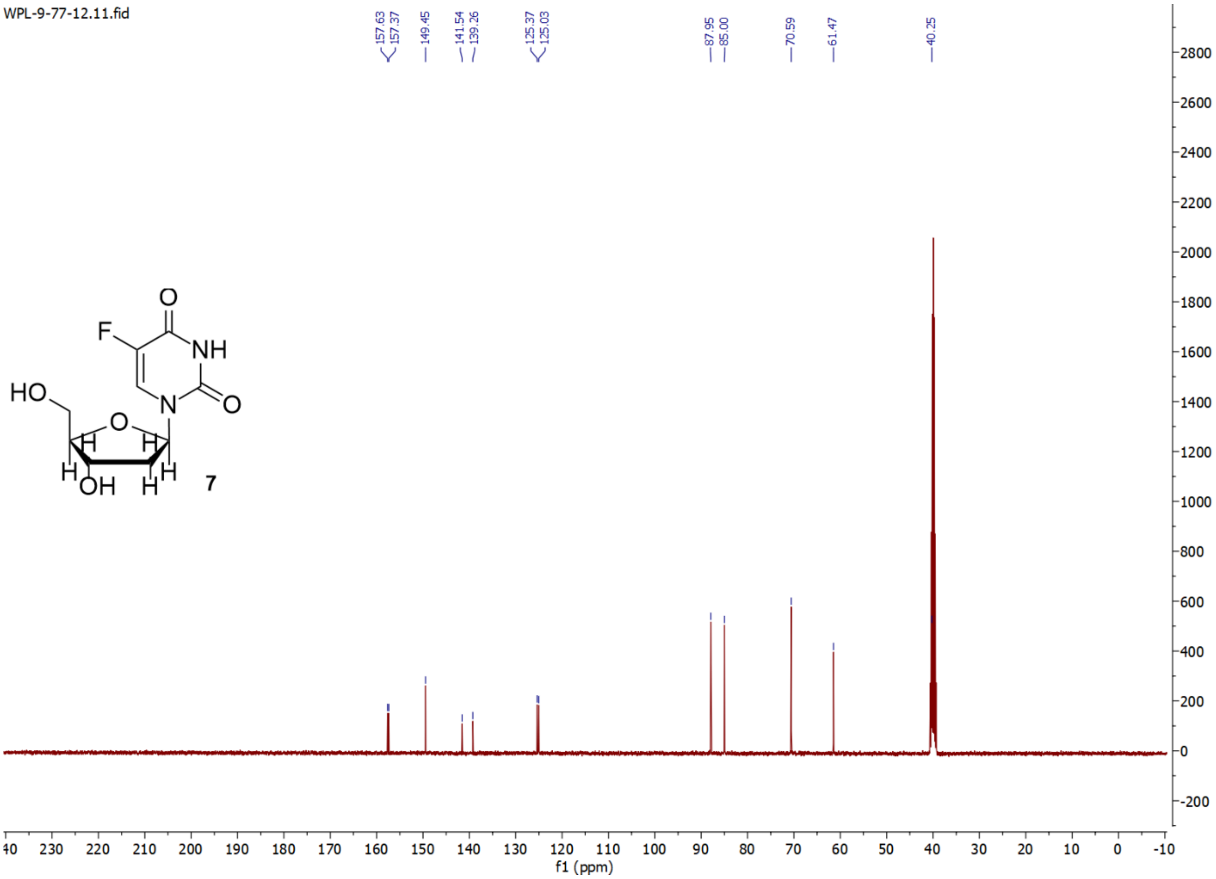

Note Figure 13 | ^{13}C NMR spectrum of product 7.

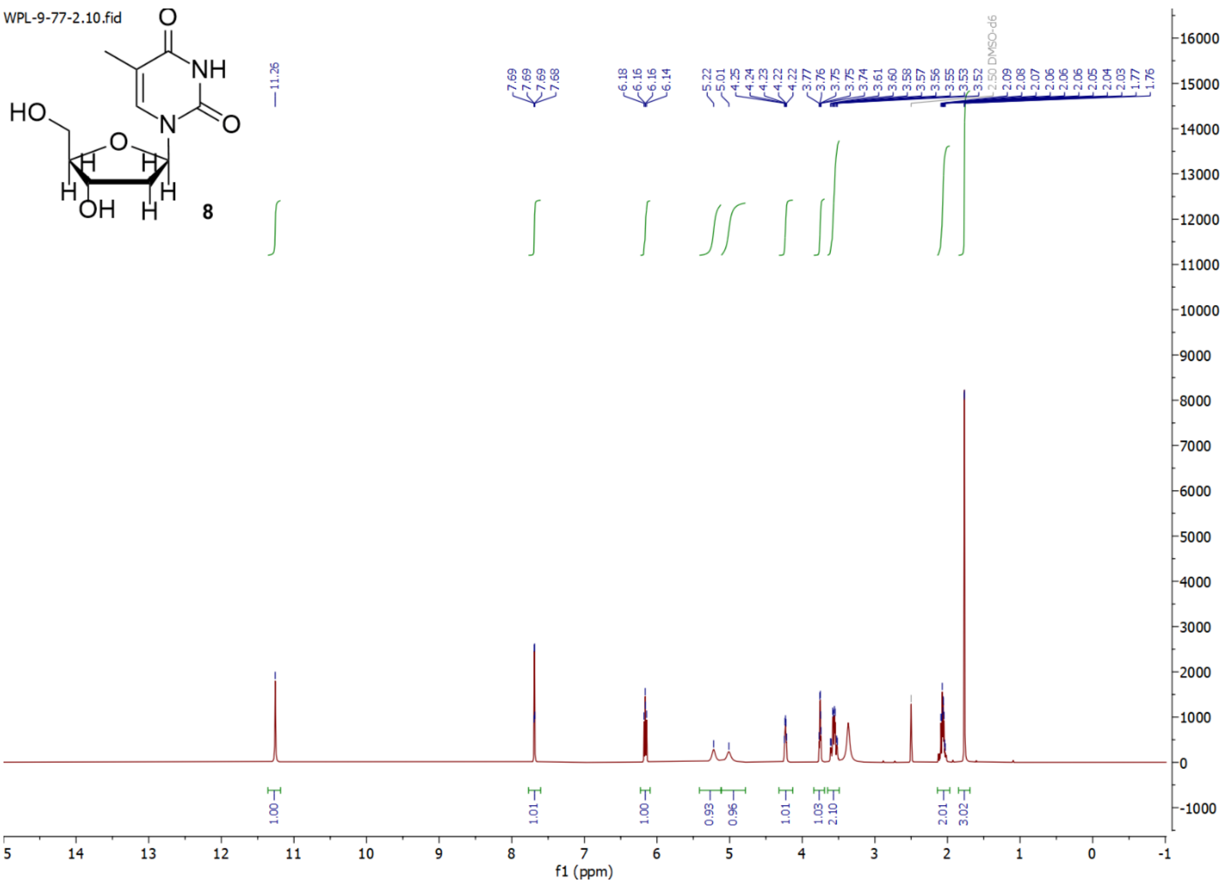

Note Figure 14 | ^{13}C NMR spectrum of product 8.

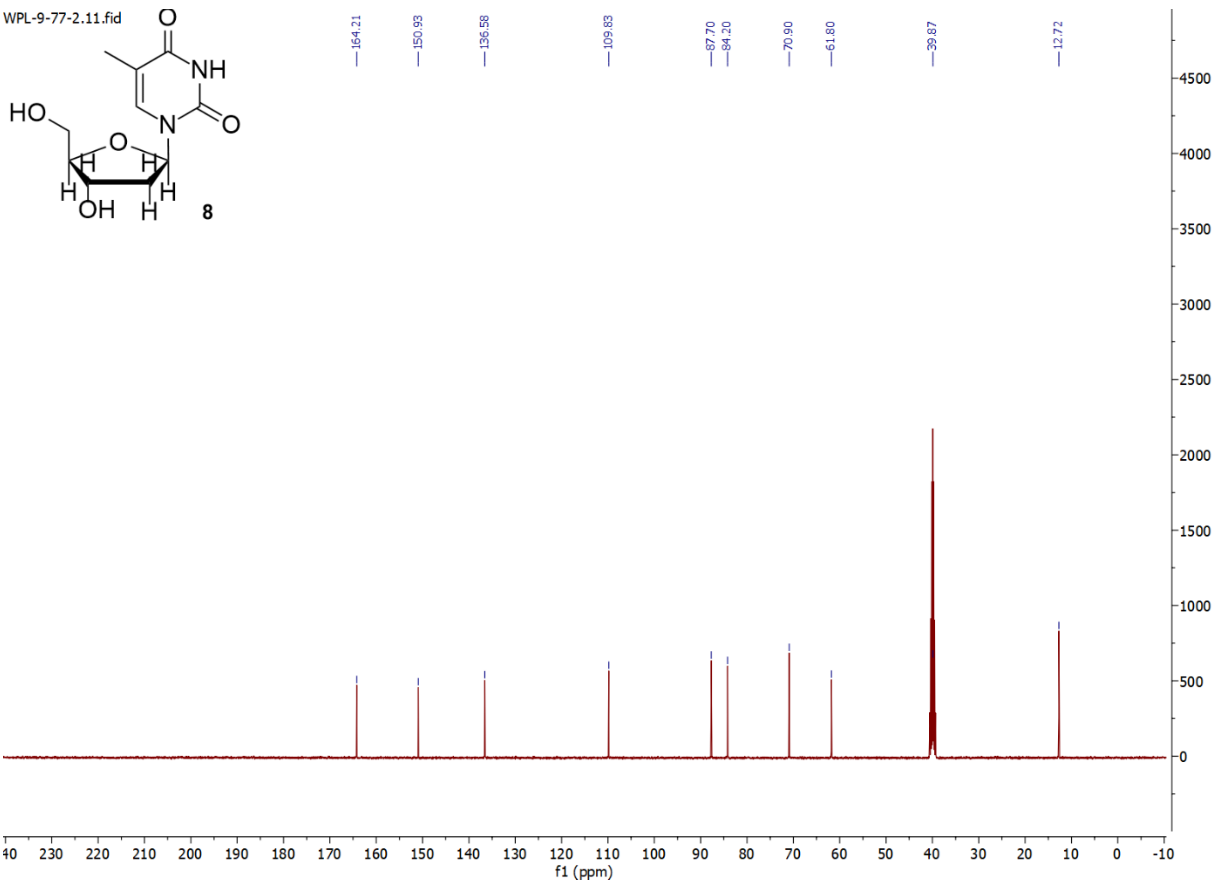

Note Figure 15 | ^{13}C NMR spectrum of product 8.

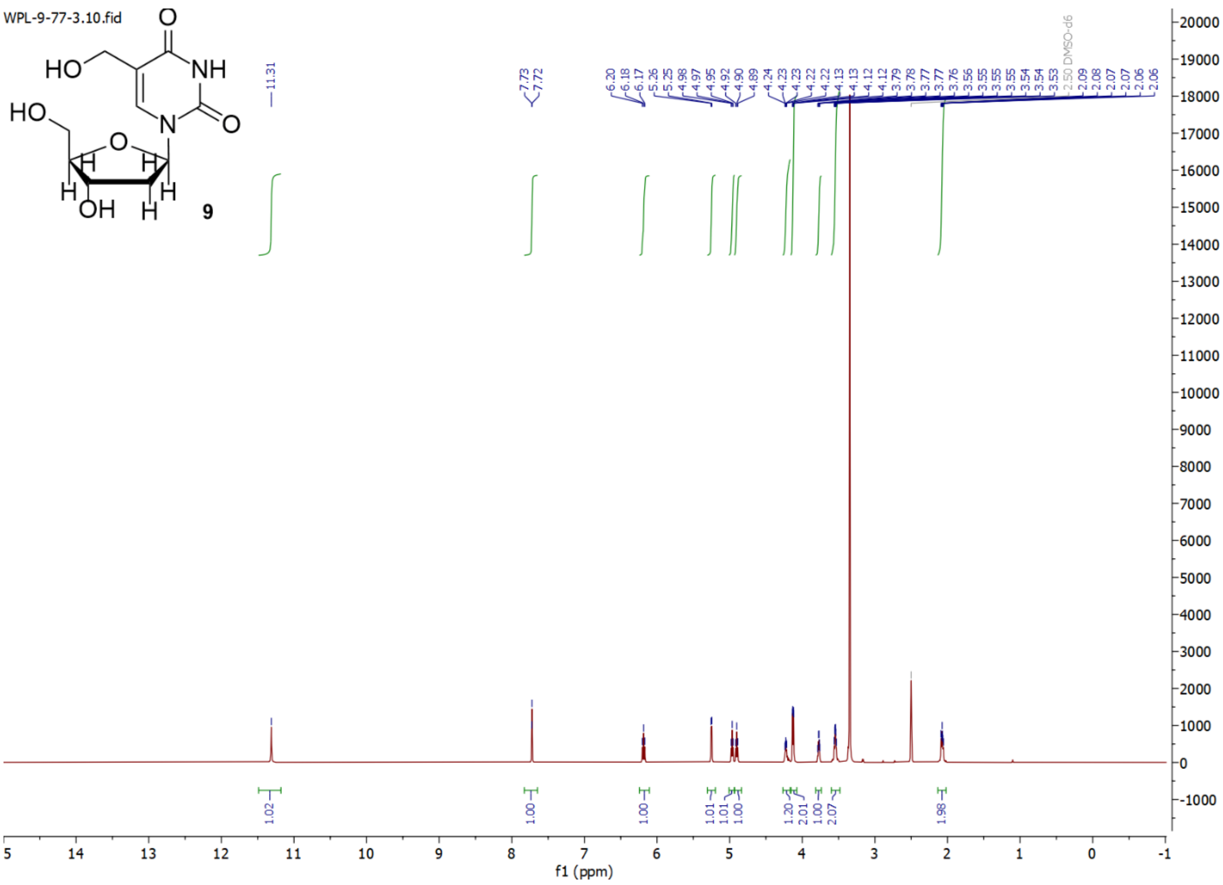

Note Figure 16 | ^{1}H NMR spectrum of product 9.

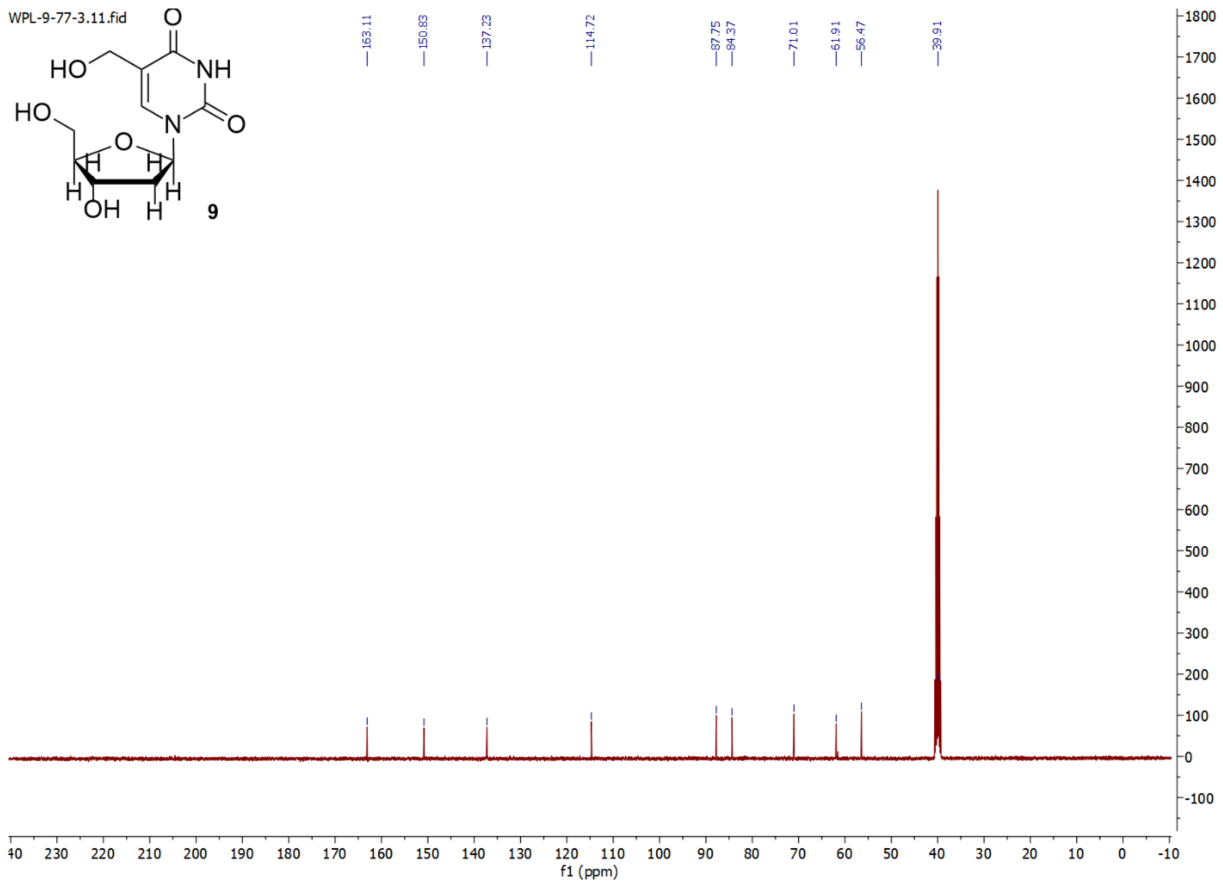

Note Figure 17 | ^{13}C NMR spectrum of product 9.

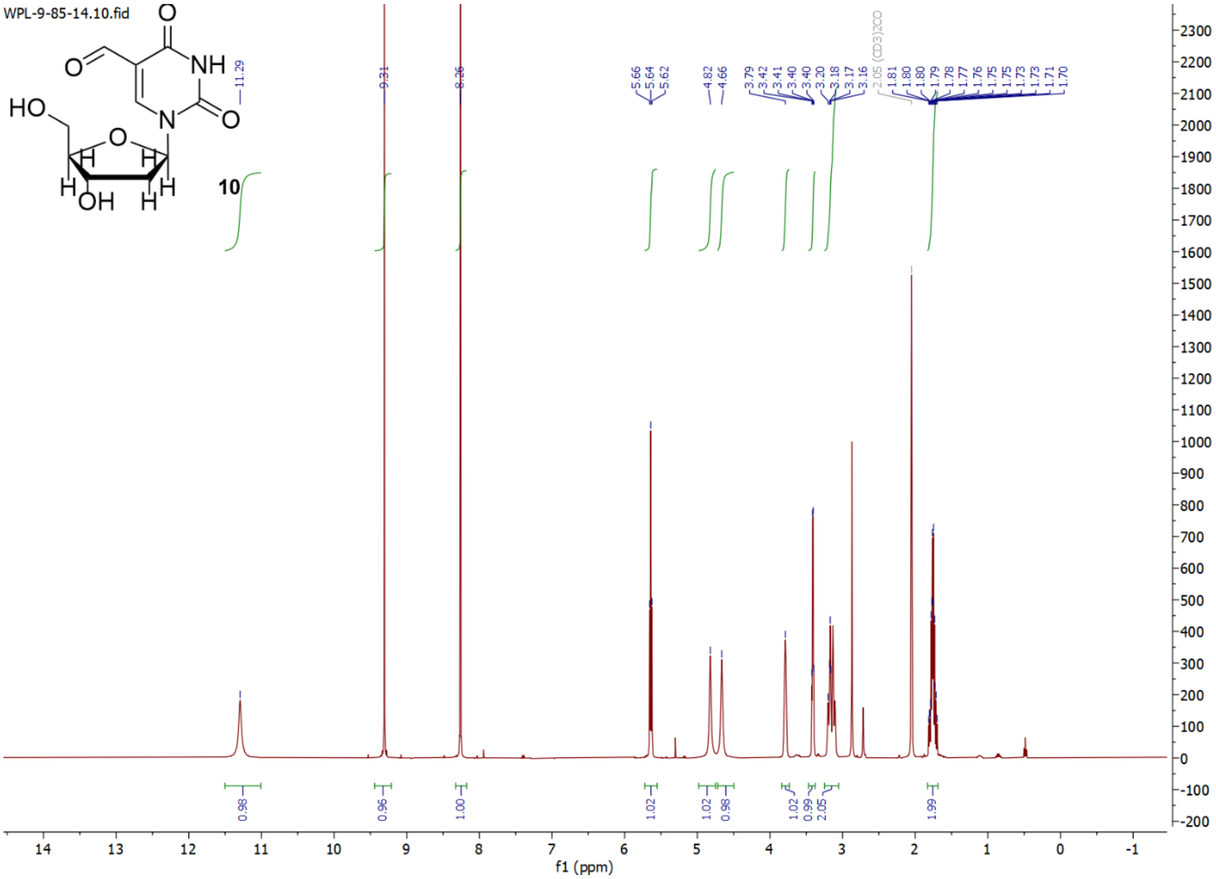

Note Figure 18 | ^{1}H NMR spectrum of product 10.

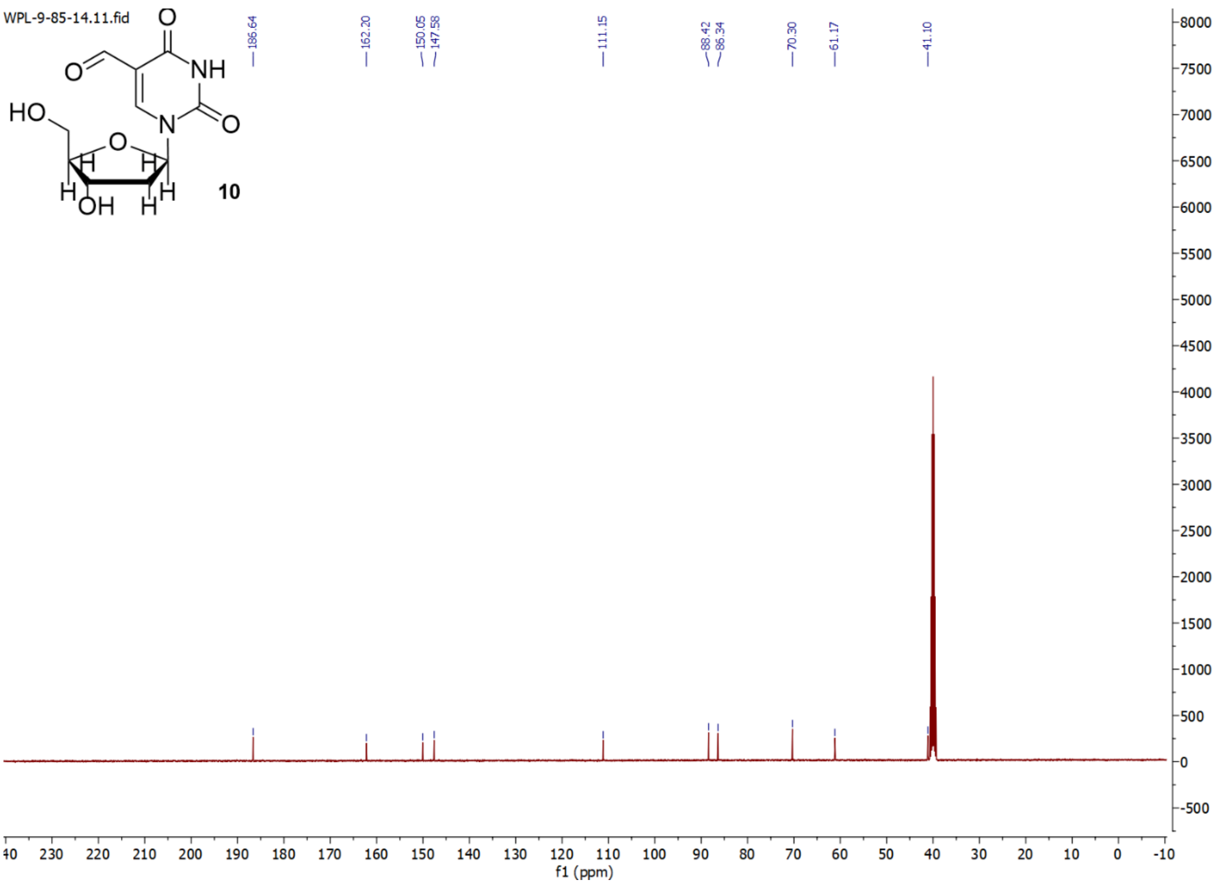

Note Figure 19 | ^{13}C NMR spectrum of product 10.

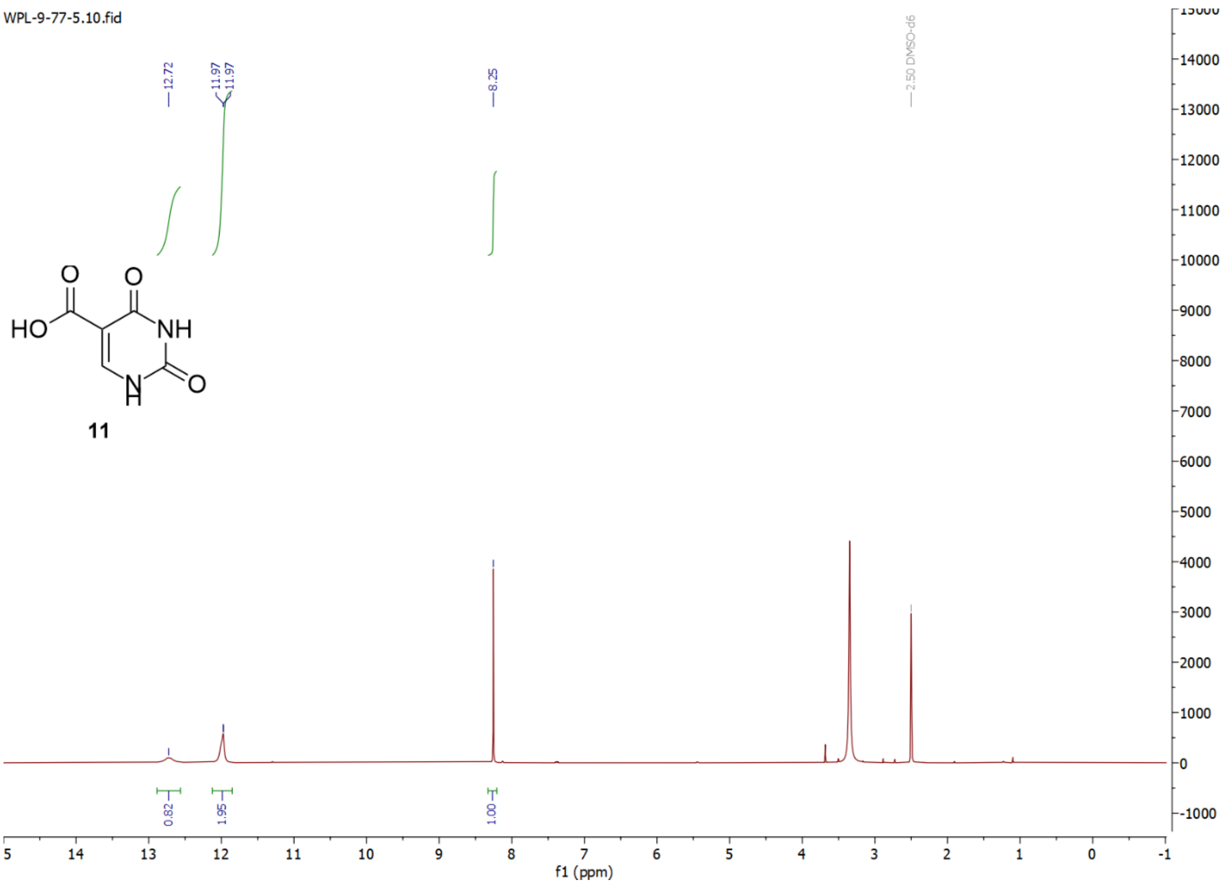

Note Figure 20 | ^{1}H NMR spectrum of product 11.

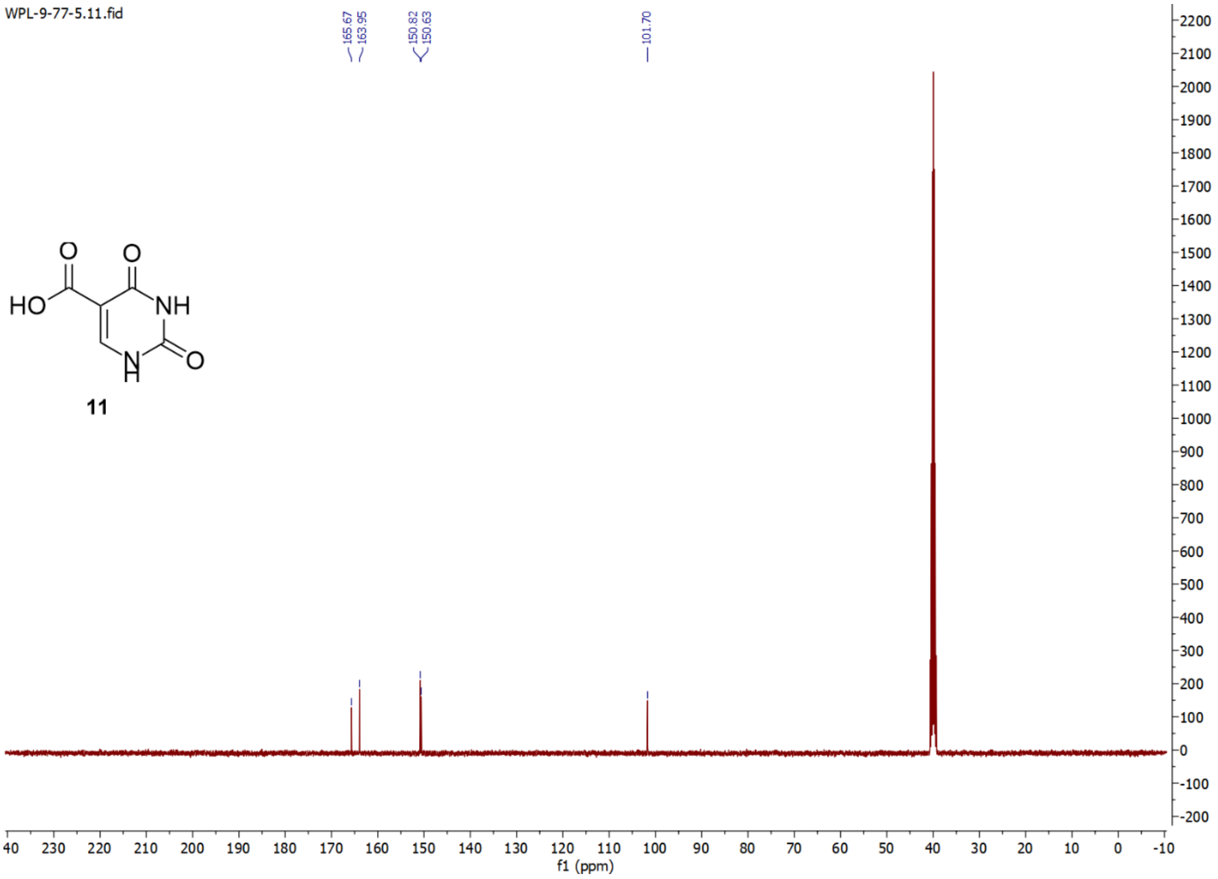

Note Figure 21 | ^{13}C NMR spectrum of product 11.

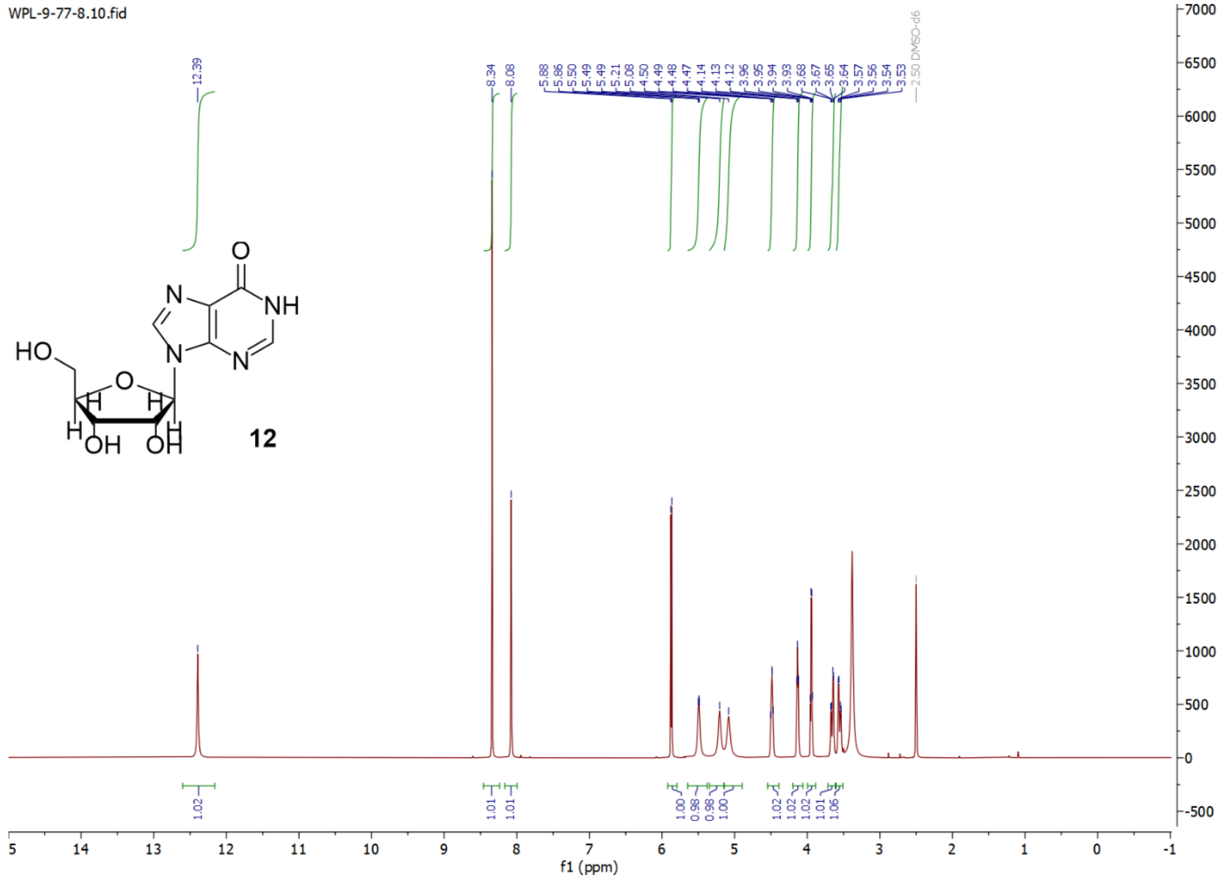

Note Figure 22 | ^{1}H NMR spectrum of product 12.

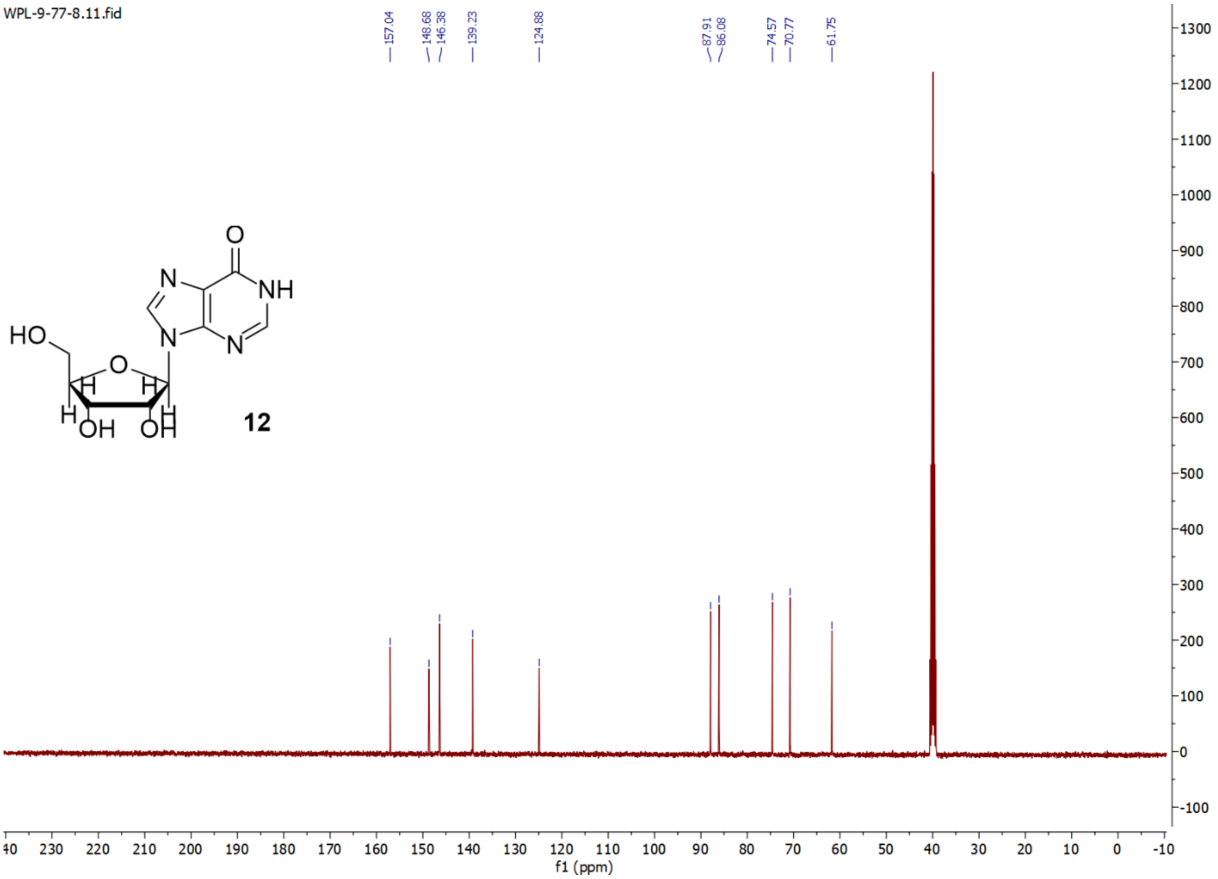

Note Figure 23 | ^{13}C NMR spectrum of product 12.

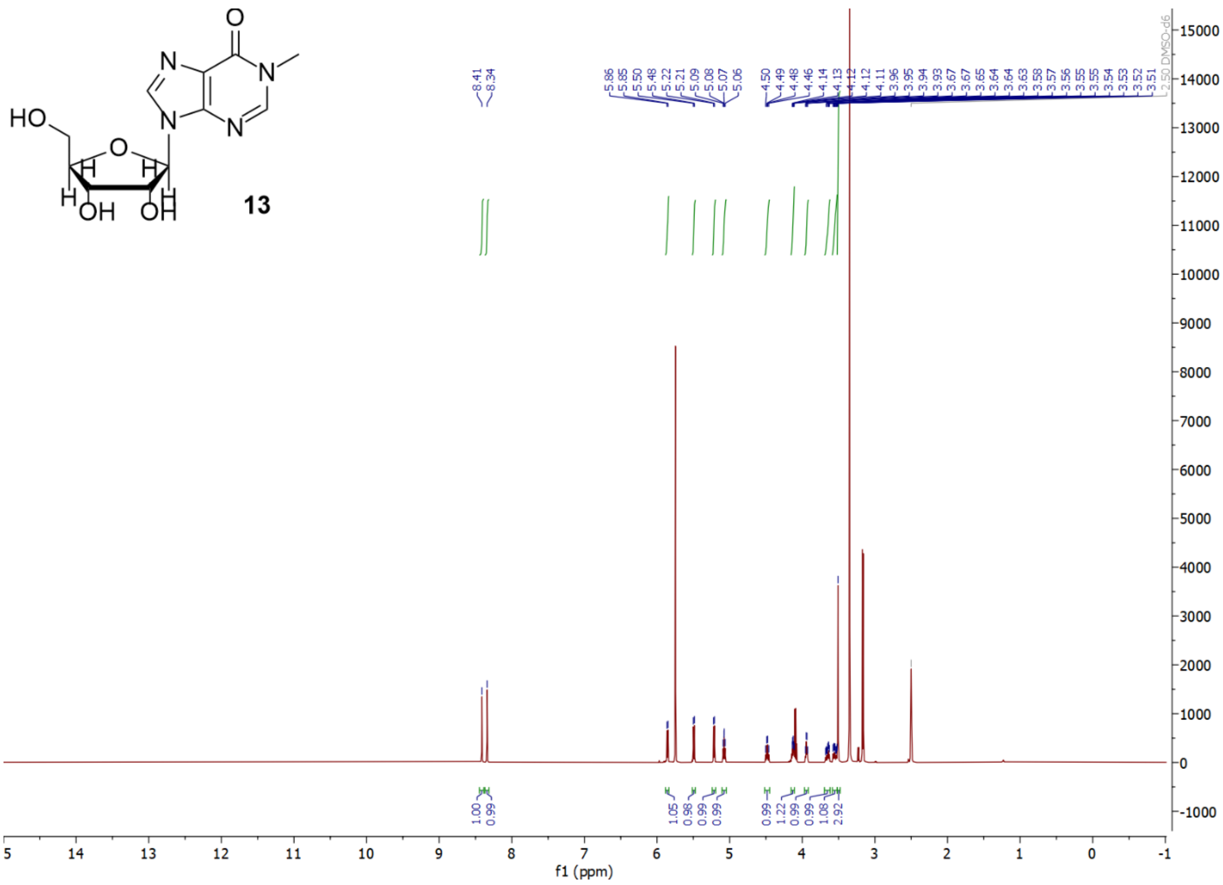

Note Figure 24 | ^{1}H NMR spectrum of product 13.

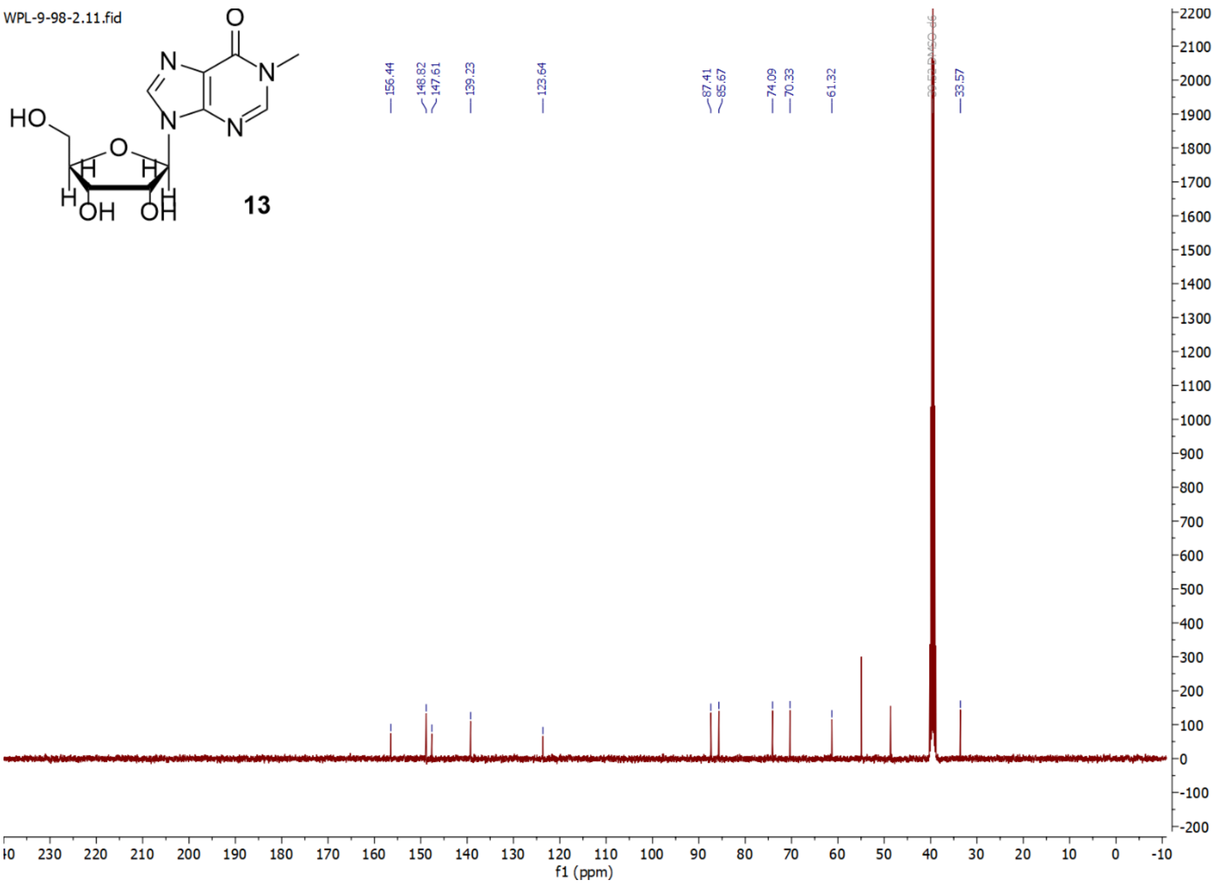

Note Figure 25 | ^{13}C NMR spectrum of product 13.

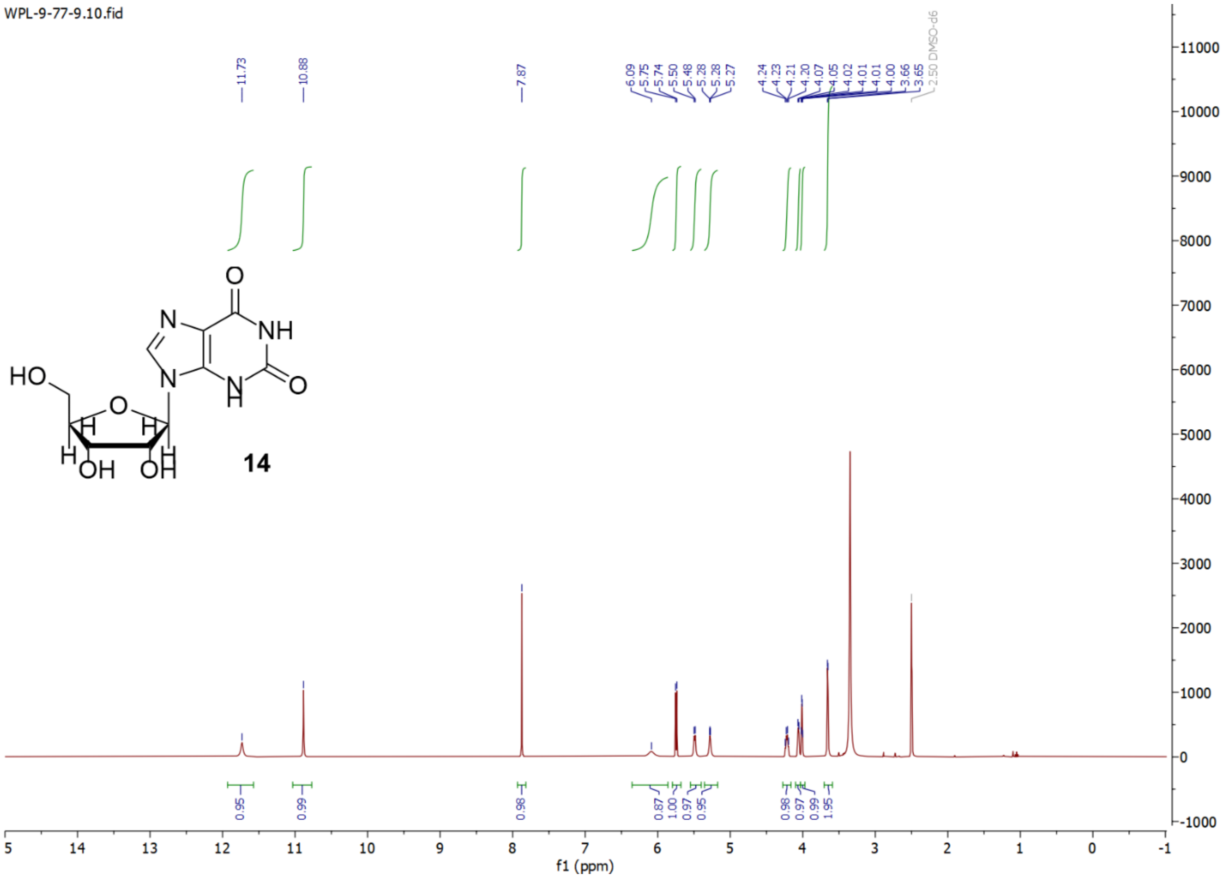

Note Figure 26 | ^{1}H NMR spectrum of product 14.

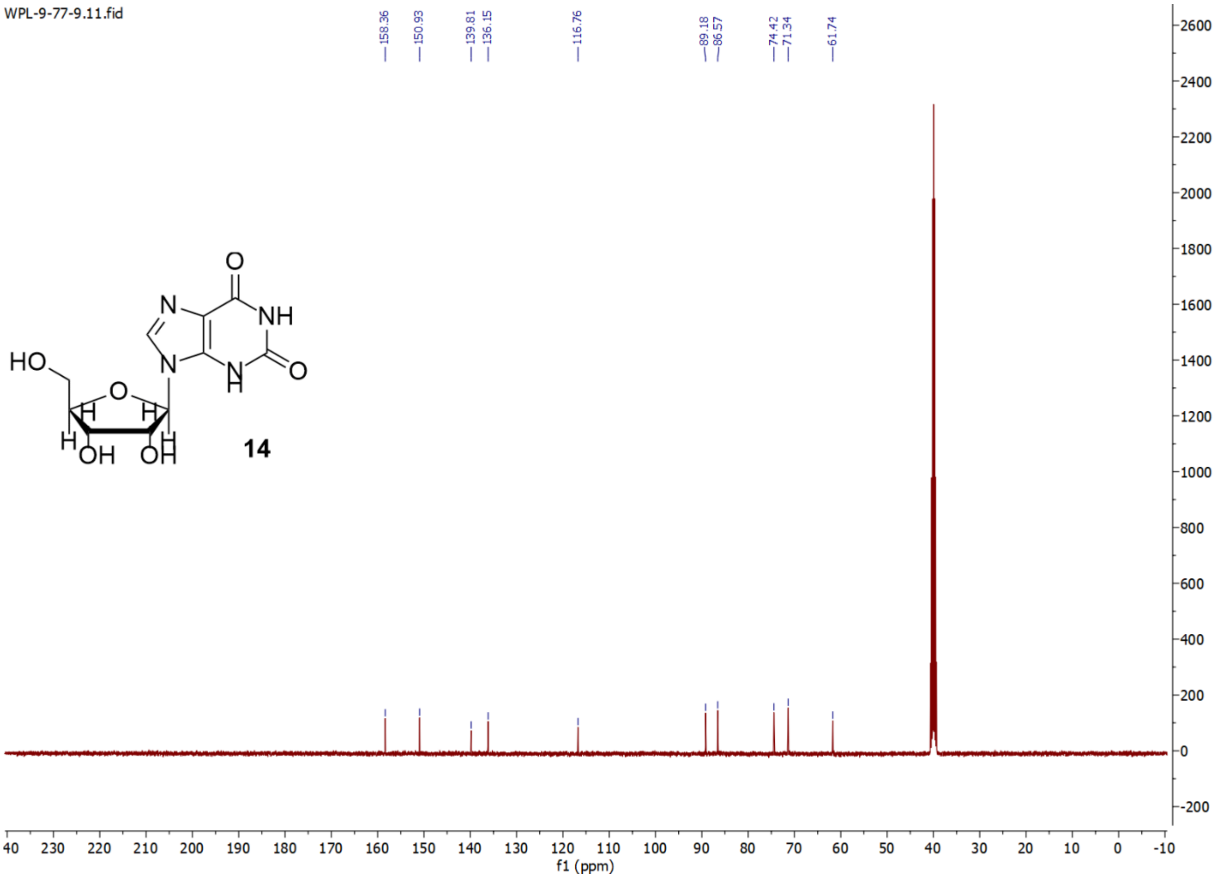

Note Figure 27 | ^{13}C NMR spectrum of product 14.

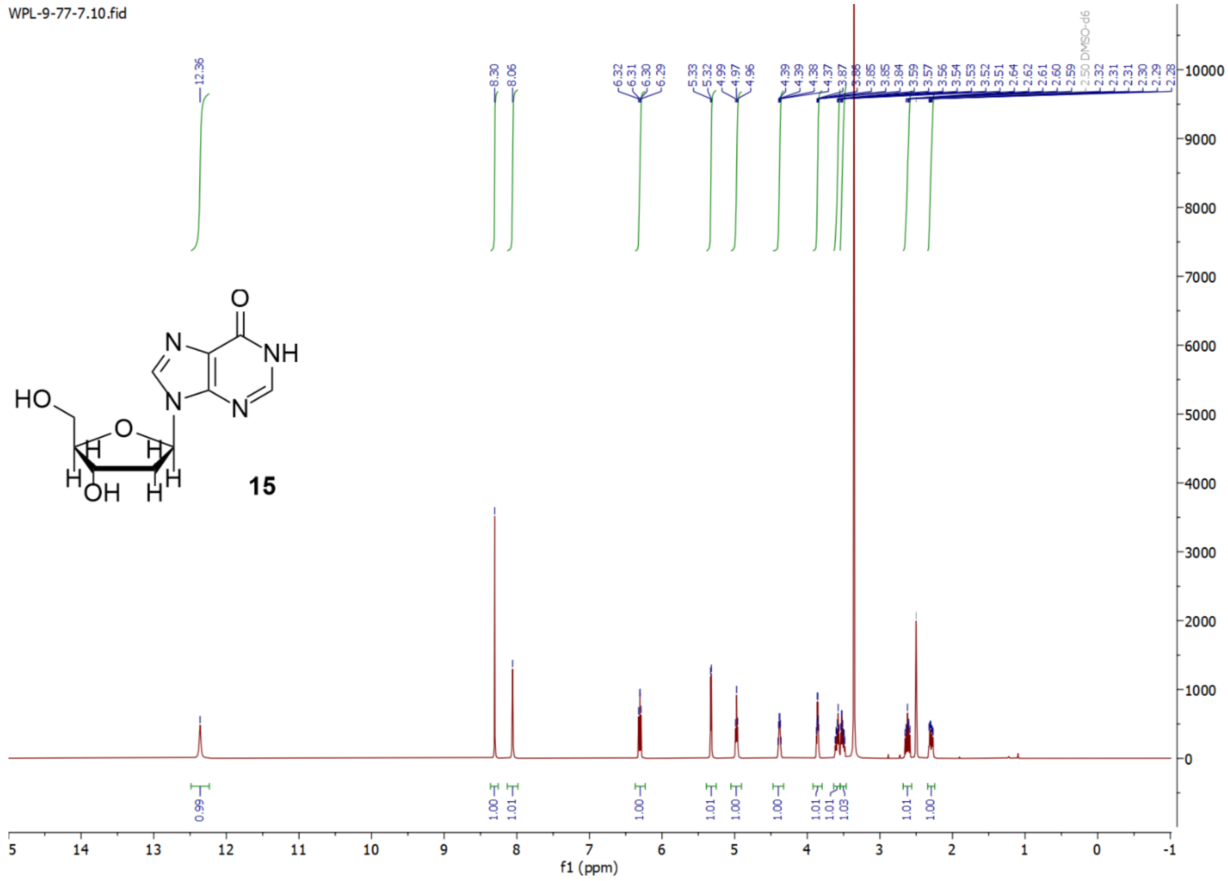

Note Figure 28 | ^{1}H NMR spectrum of product 15.

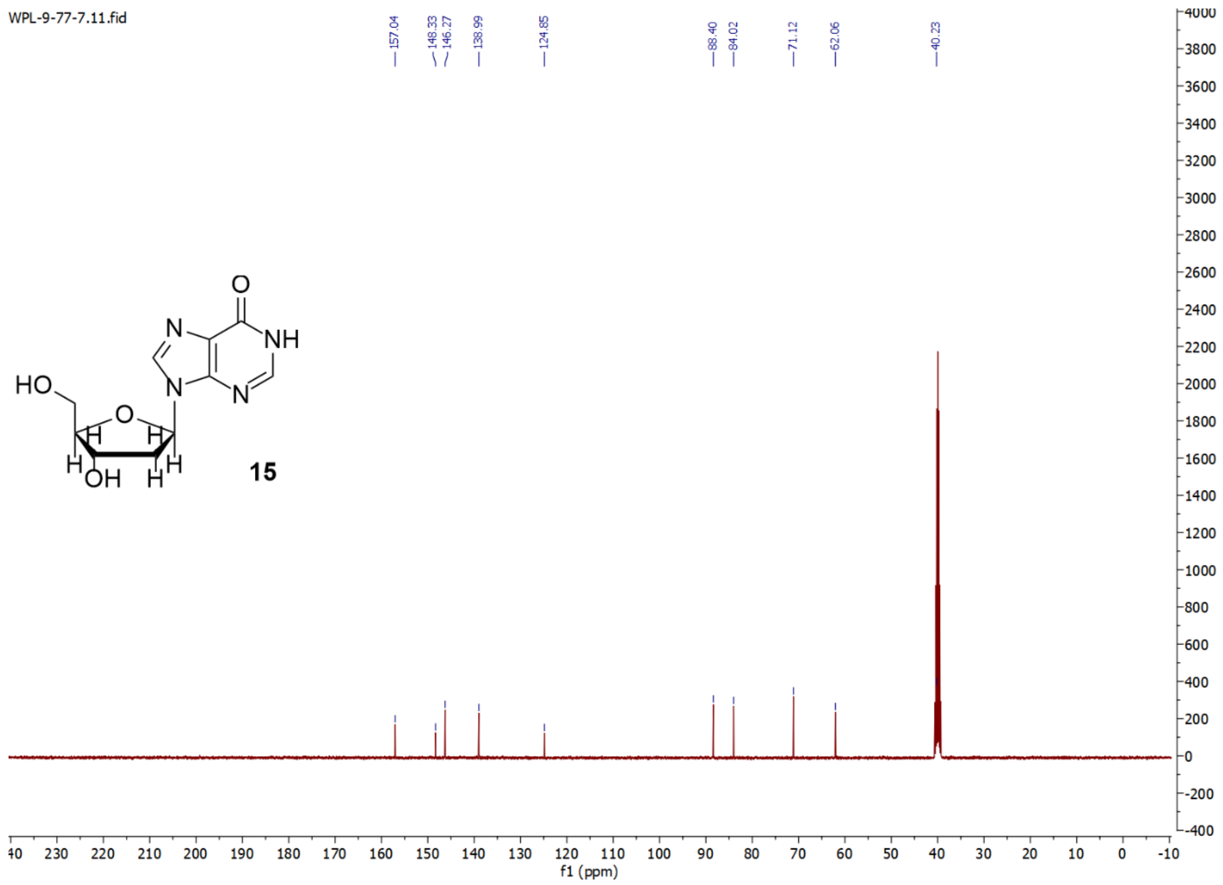

Note Figure 29 | ^{13}C NMR spectrum of product 15.

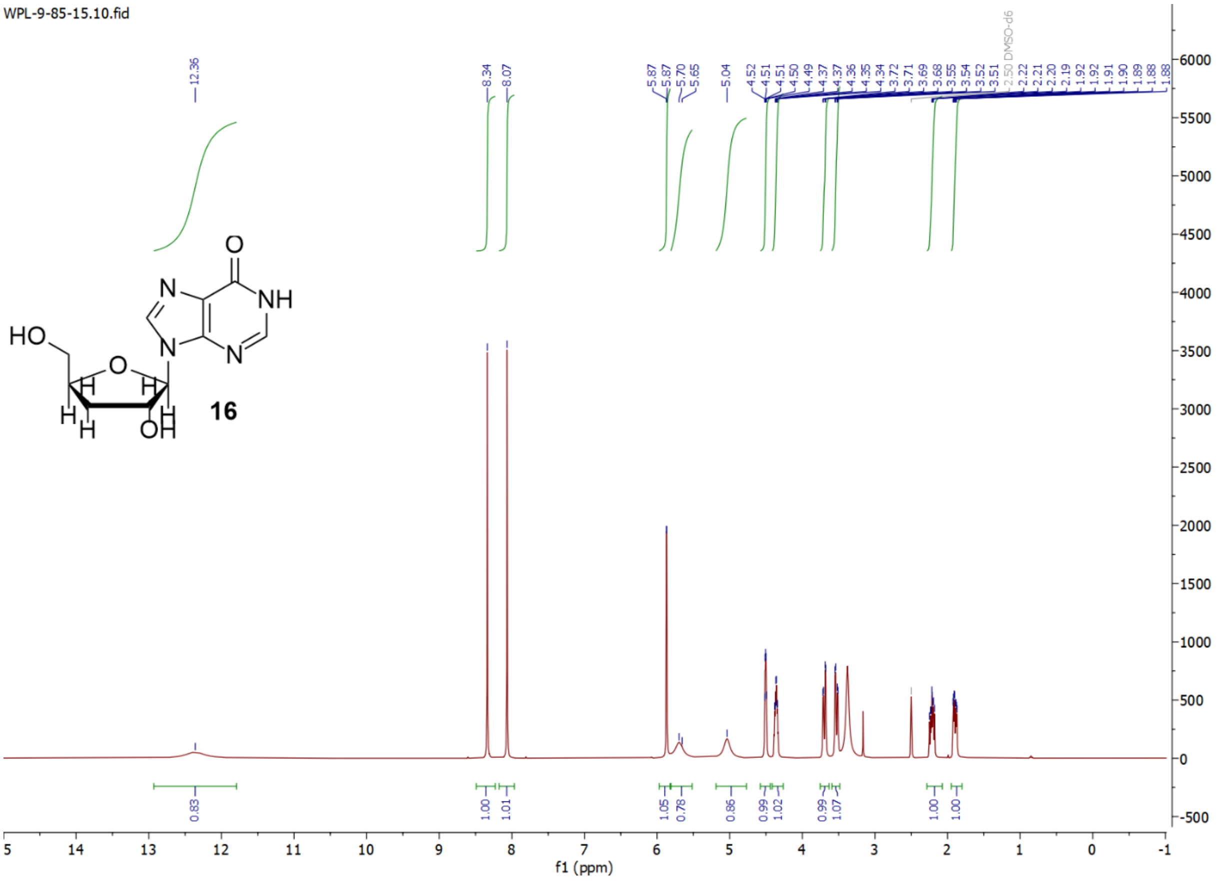

Note Figure 30 | ^{1}H NMR spectrum of product 16.

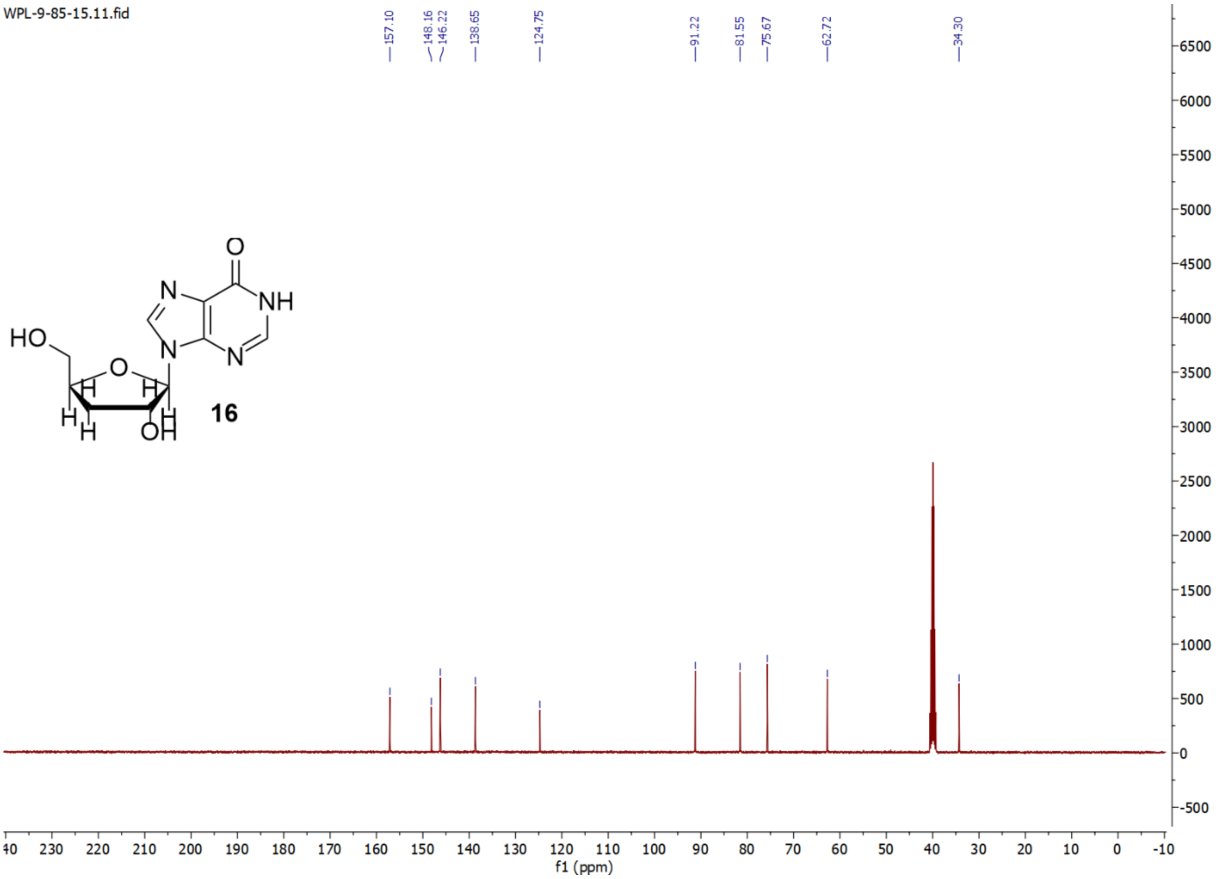

Note Figure 31 | ^{13}C NMR spectrum of product 16.

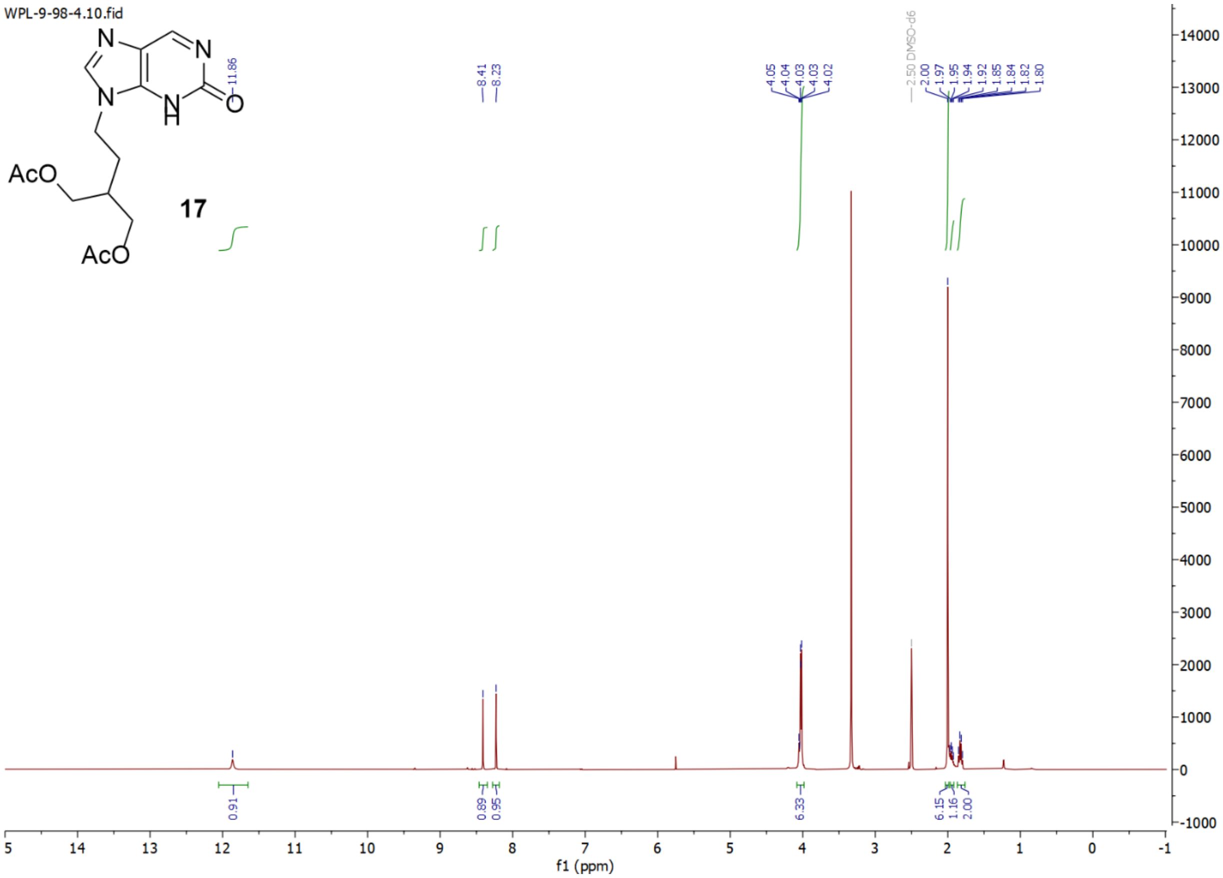

Note Figure 32 | ^{1}H NMR spectrum of product 17.

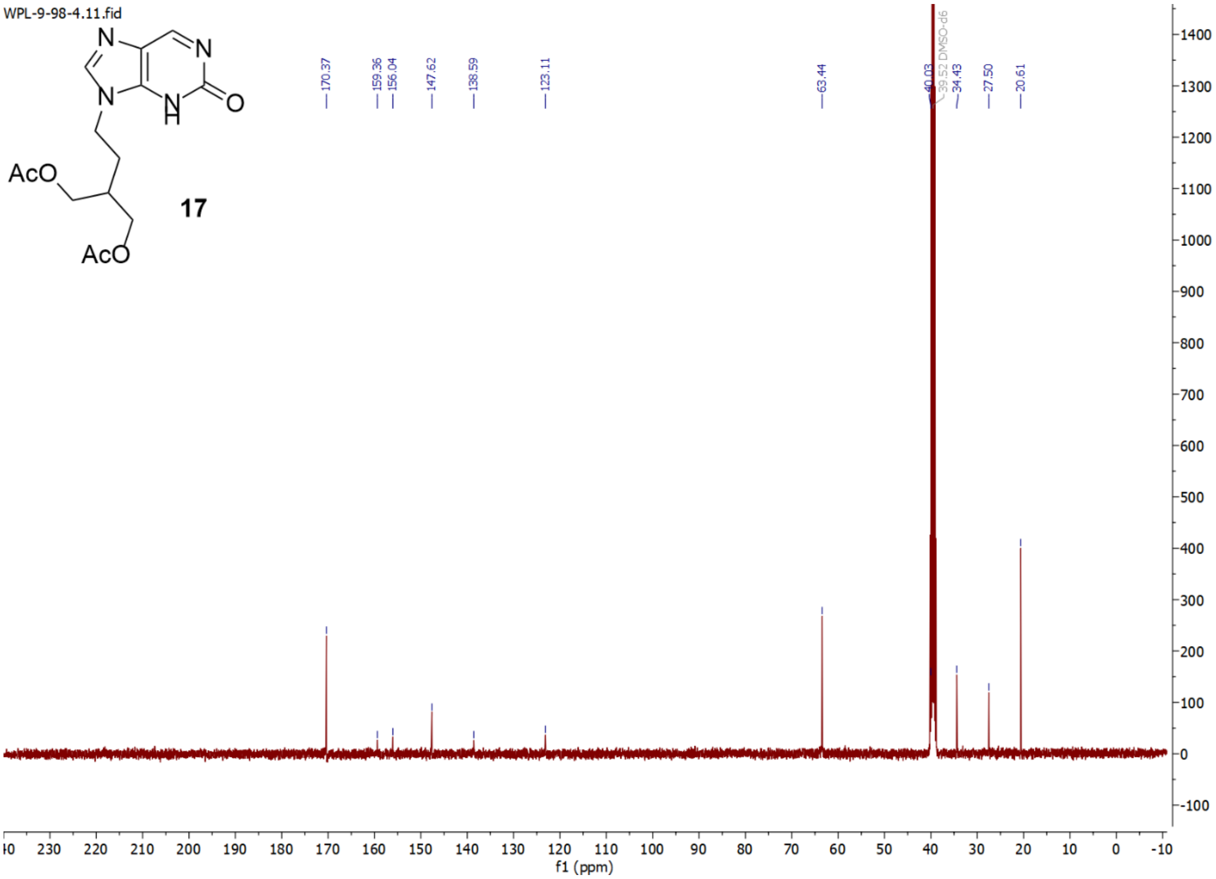

Note Figure 33 | ^{13}C NMR spectrum of product 17.

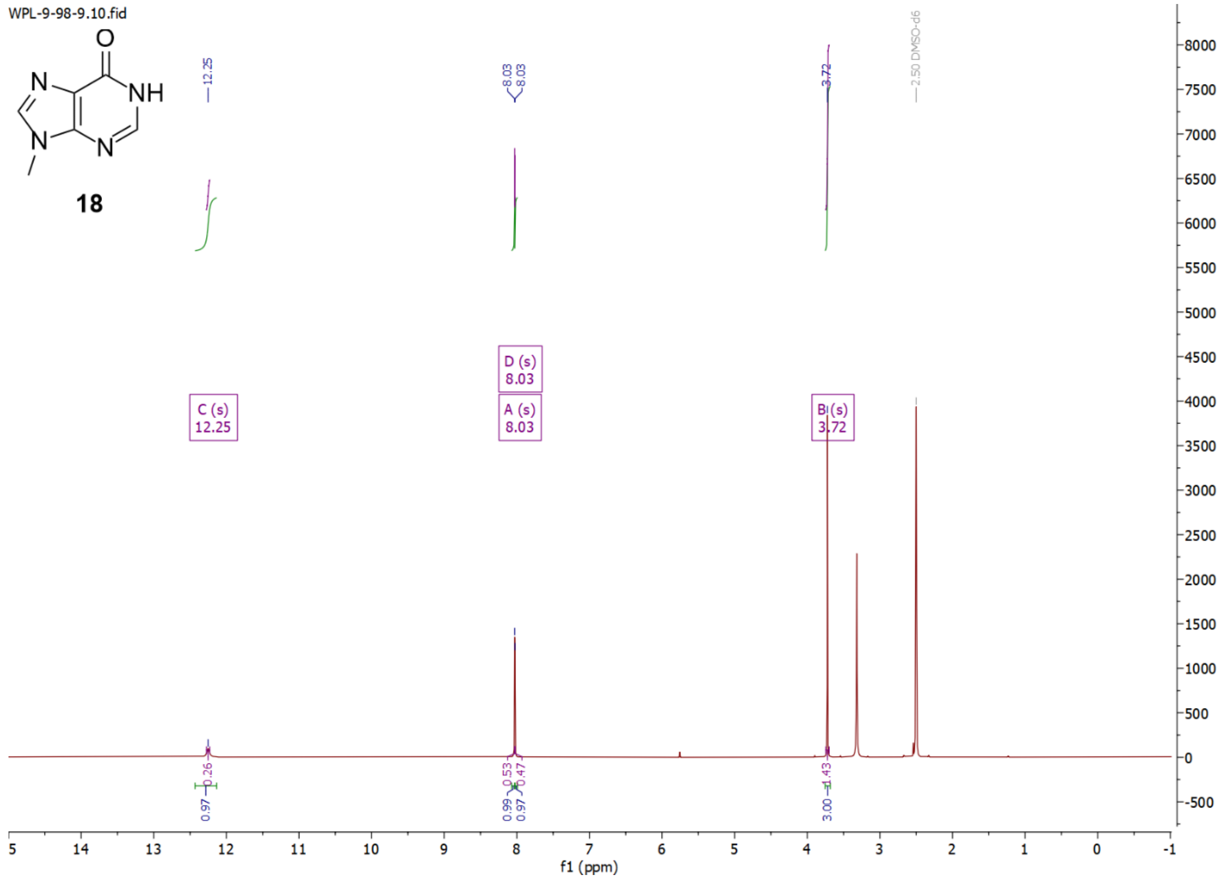

Note Figure 34 | ^{1}H NMR spectrum of product 18.

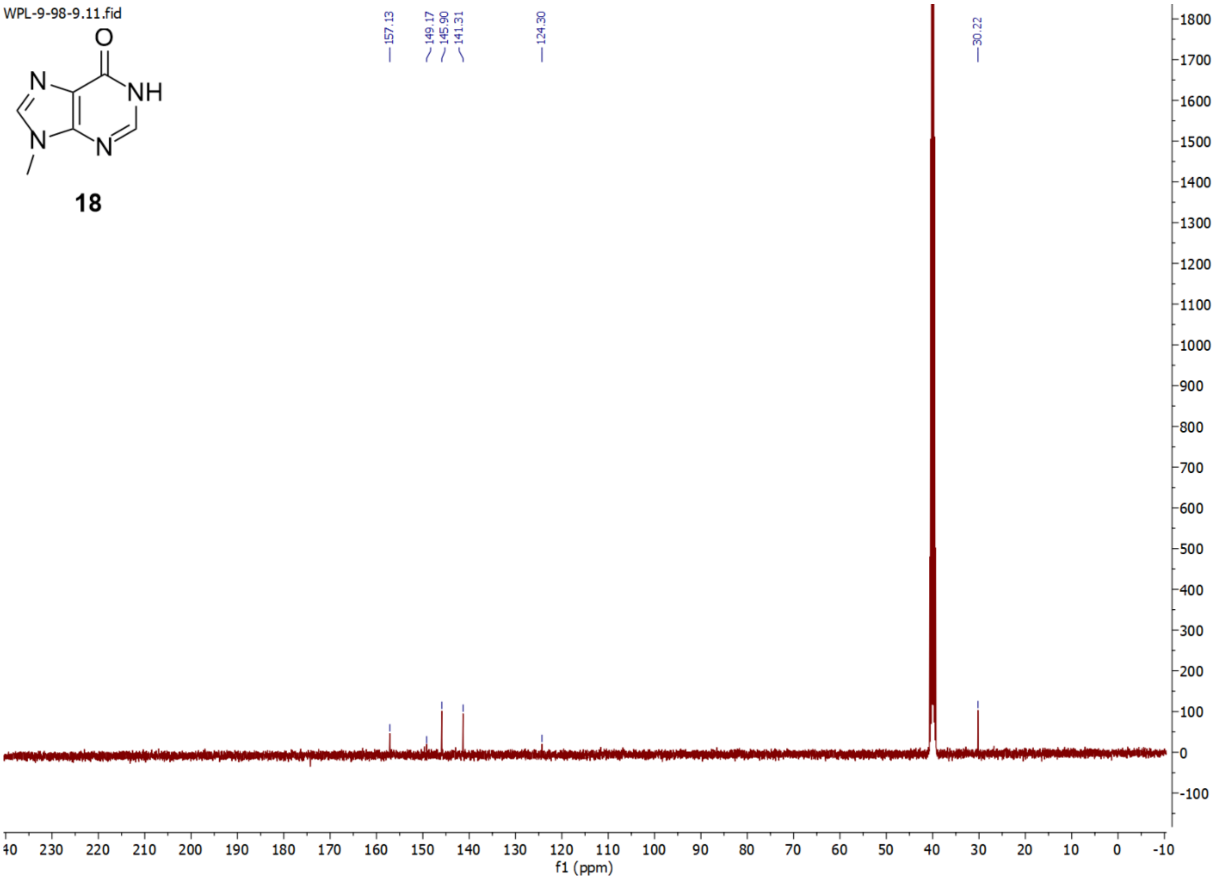

Note Figure 35 | ^{13}C NMR spectrum of product 18.

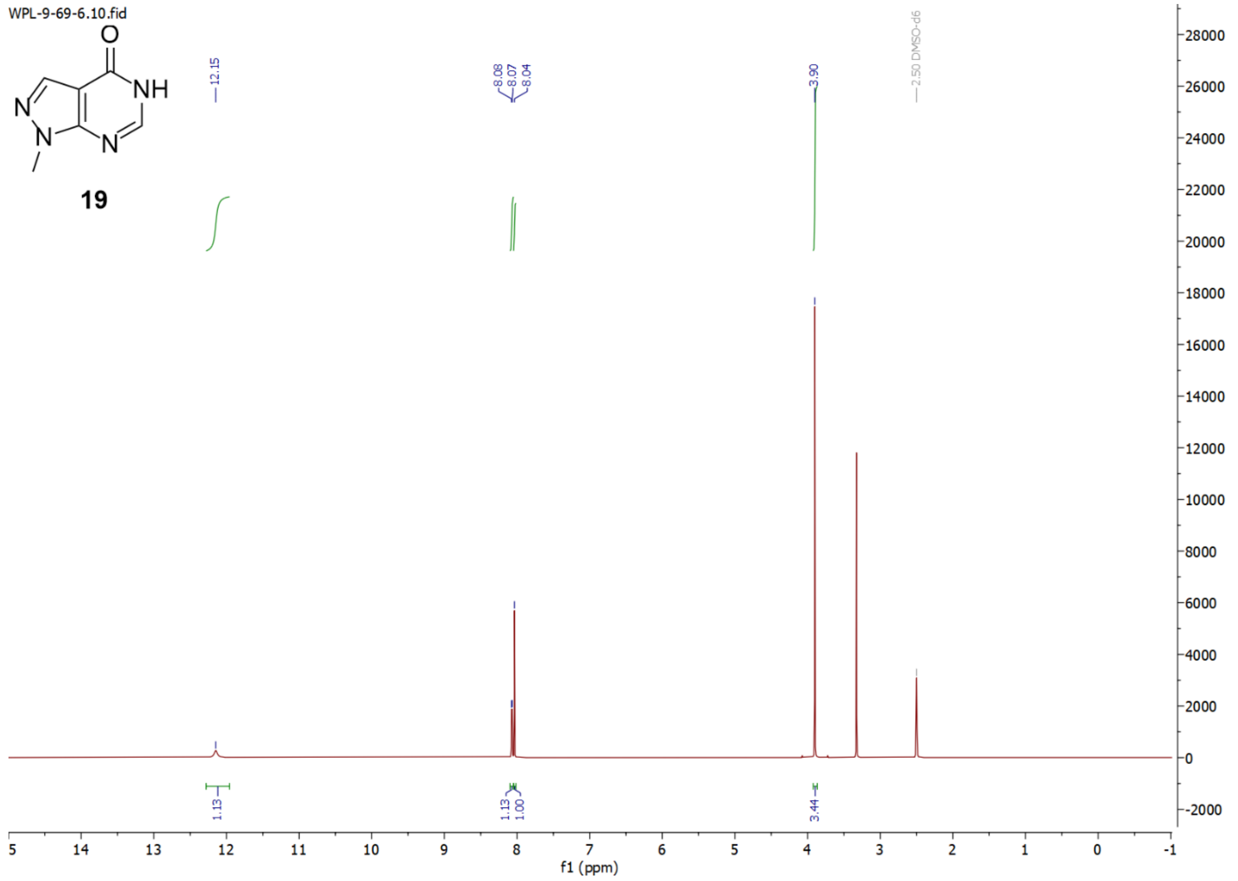

Note Figure 36 | ^{1}H NMR spectrum of product 19.

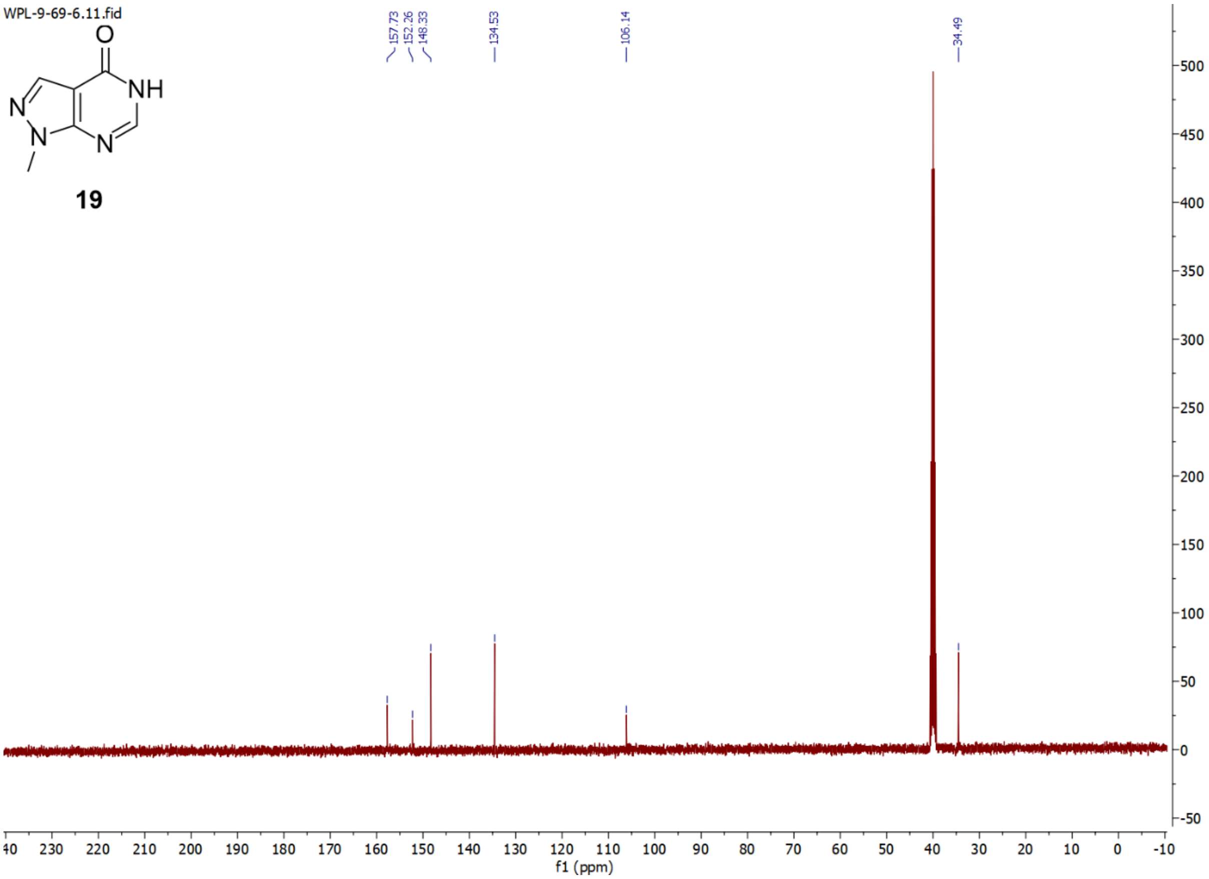

Note Figure 37 | ^{13}C NMR spectrum of product 19.

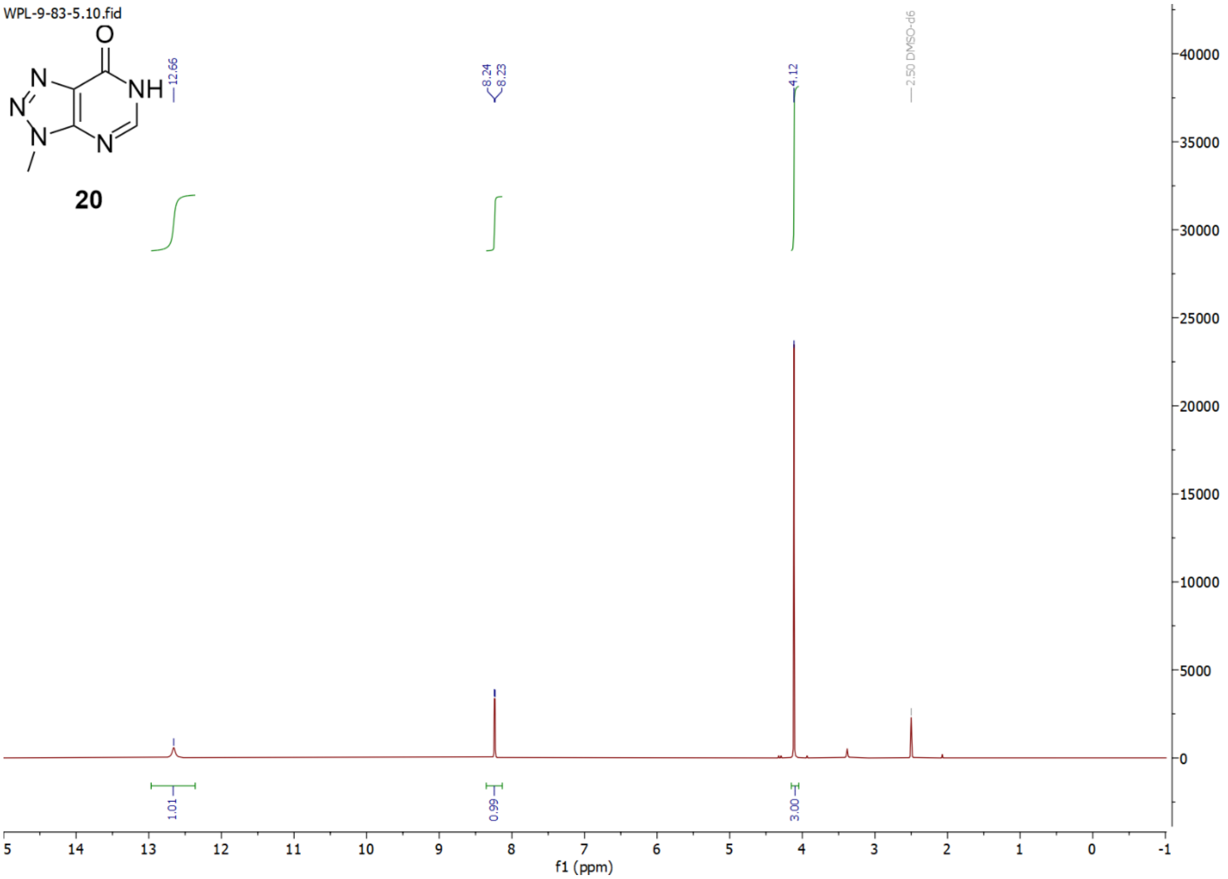

Note Figure 38 | ^{1}H NMR spectrum of product 20.

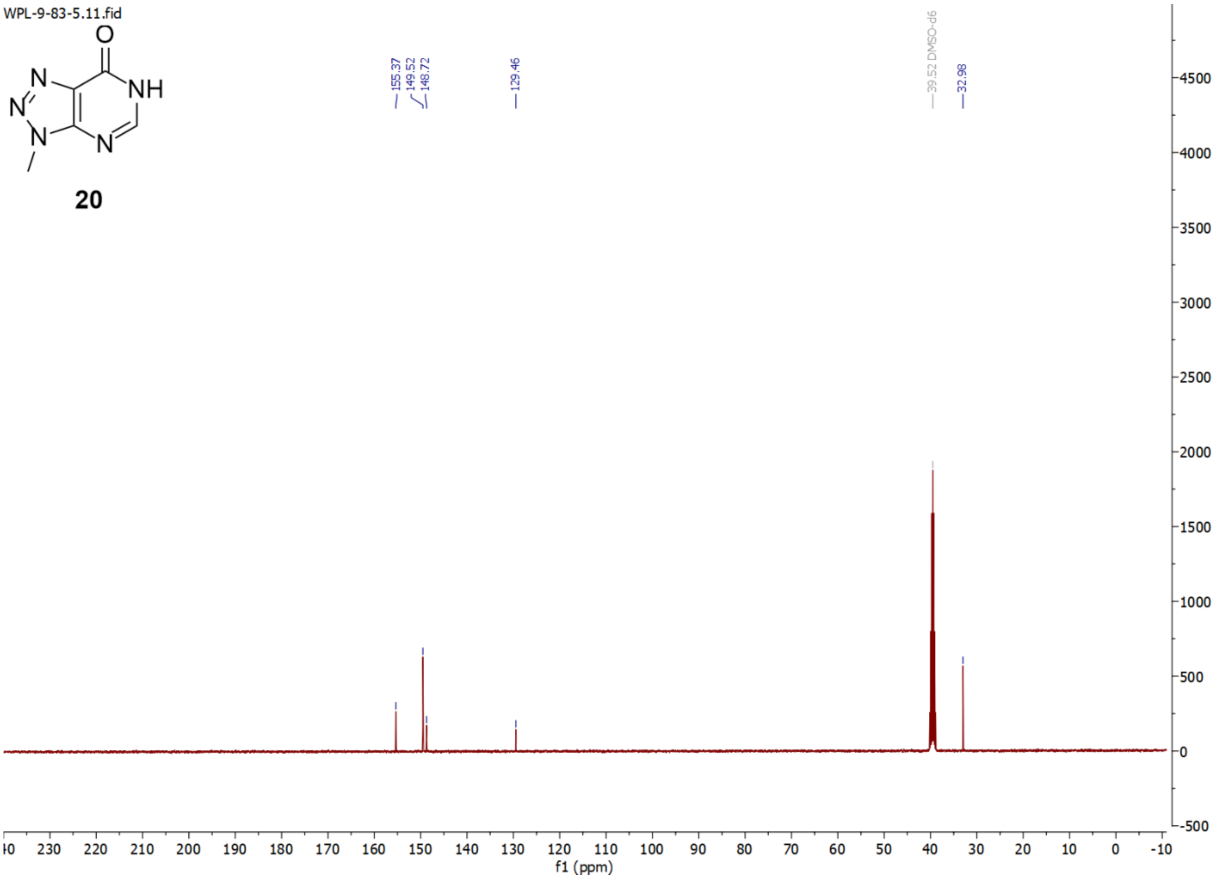

Note Figure 39 | ^{13}C NMR spectrum of product 20.

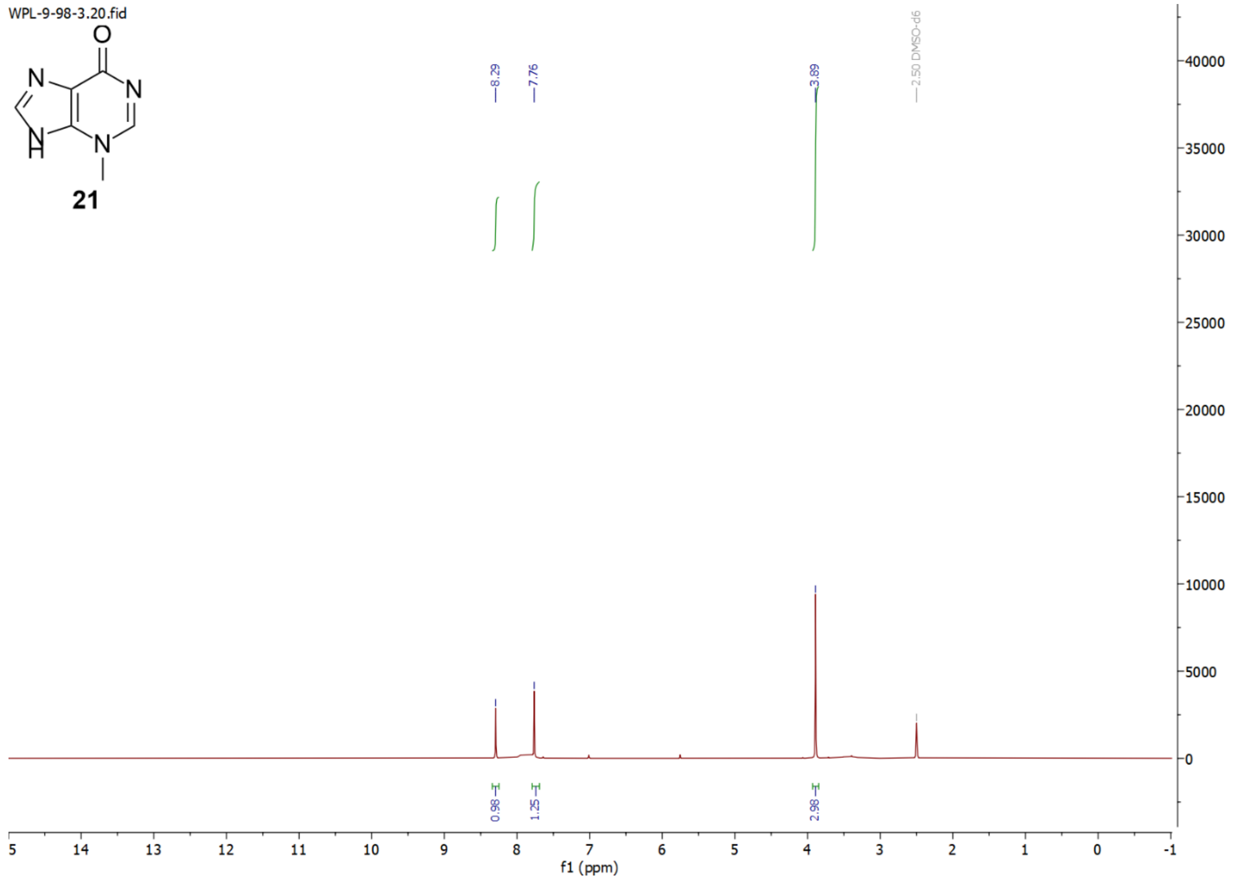

Note Figure 40 | ^{1}H NMR spectrum of product 21.

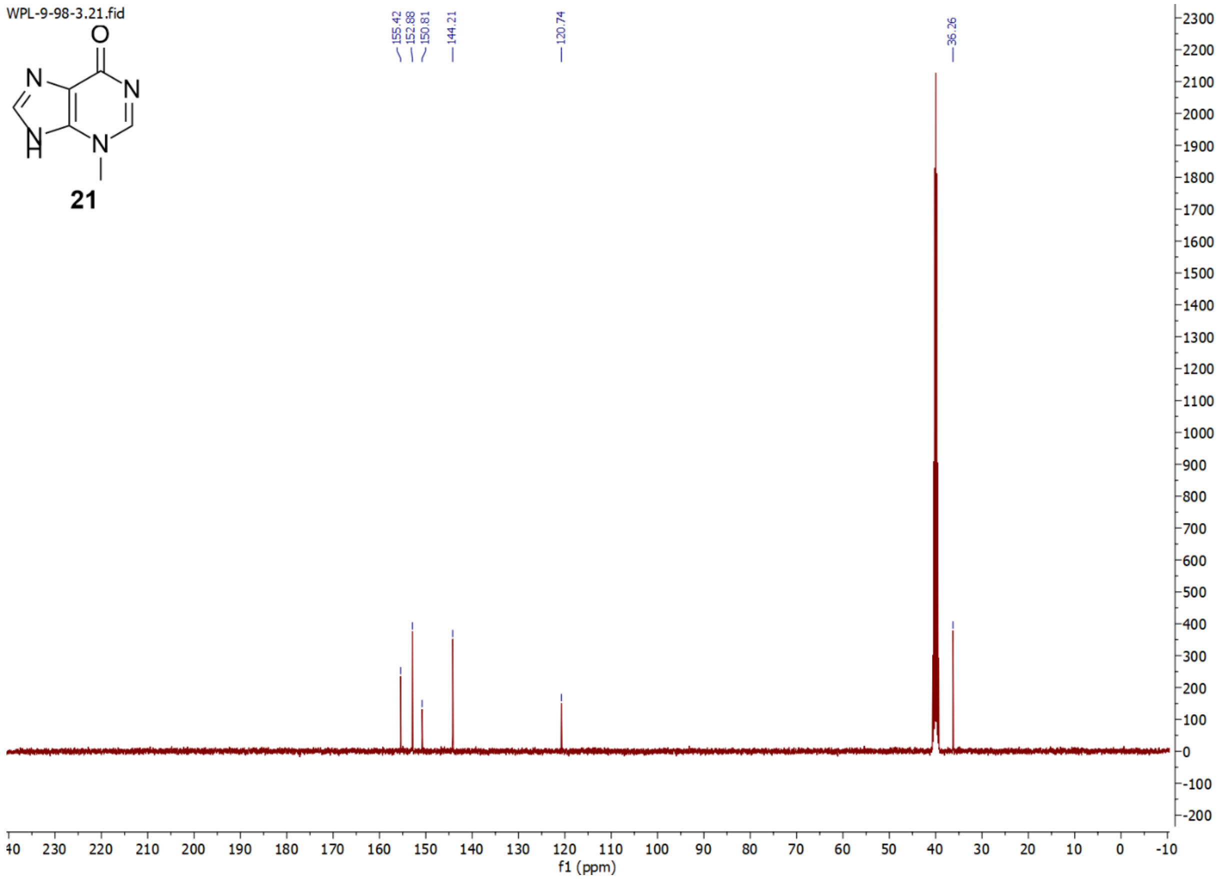

Note Figure 41 | ^{13}C NMR spectrum of product 21.

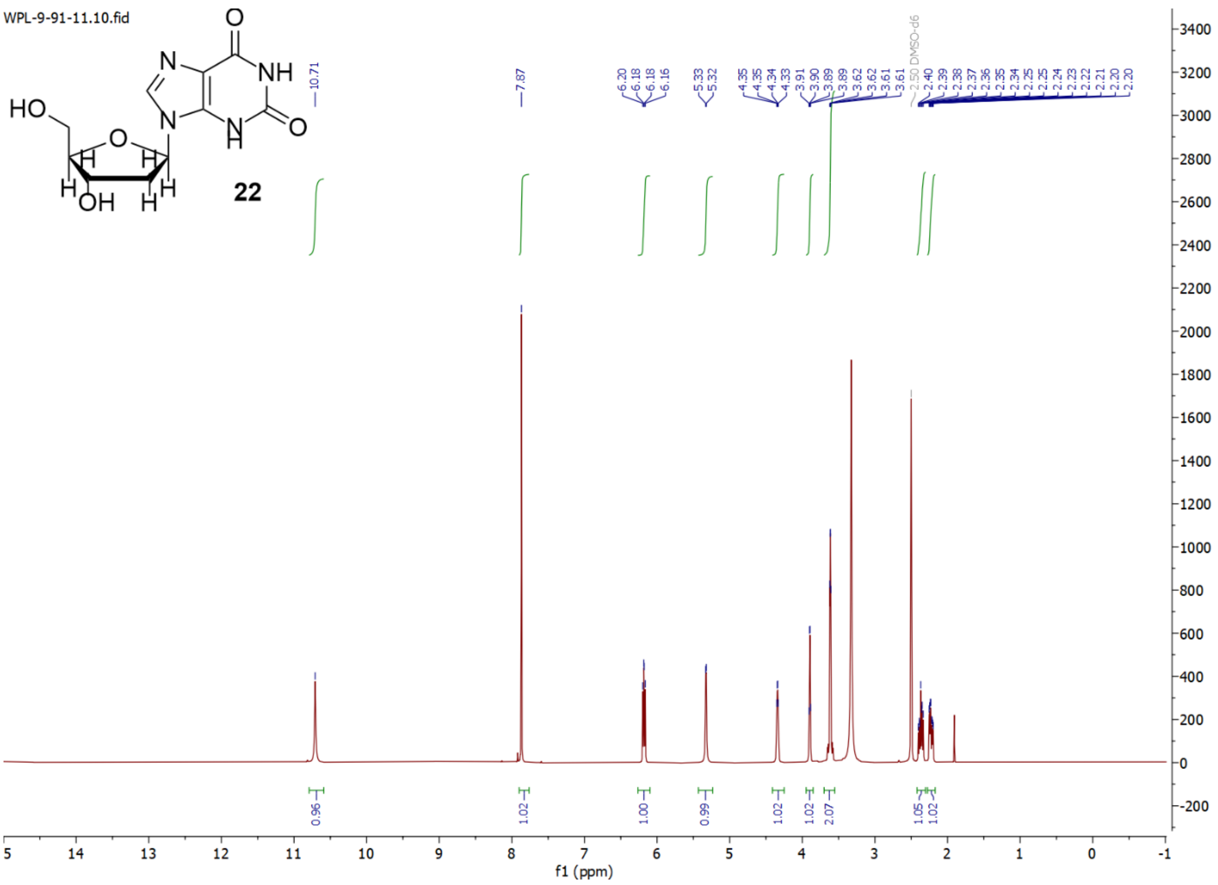

Note Figure 42 | ^{1}H NMR spectrum of product 22.

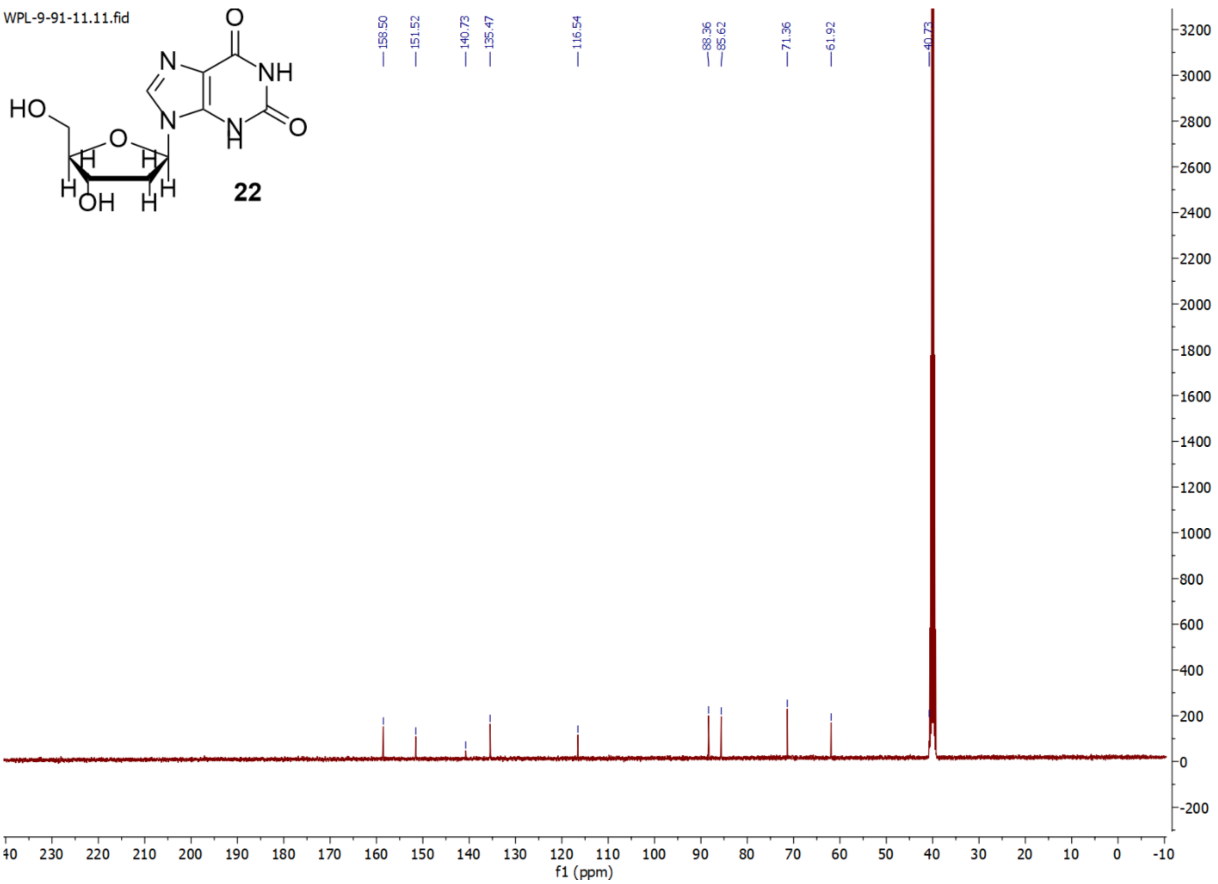

Note Figure 43 | ^{13}C NMR spectrum of product 22.

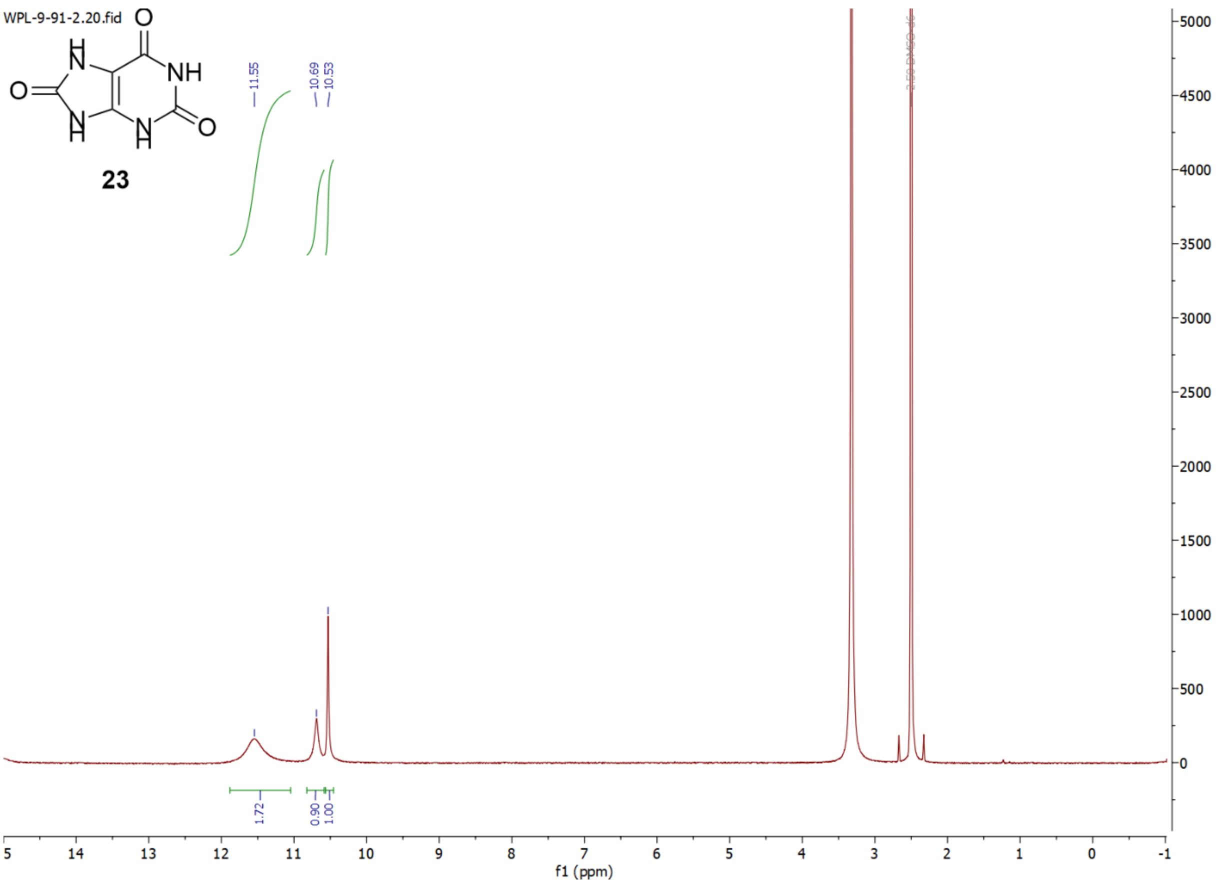

Note Figure 44 | ^{1}H NMR spectrum of product 23.

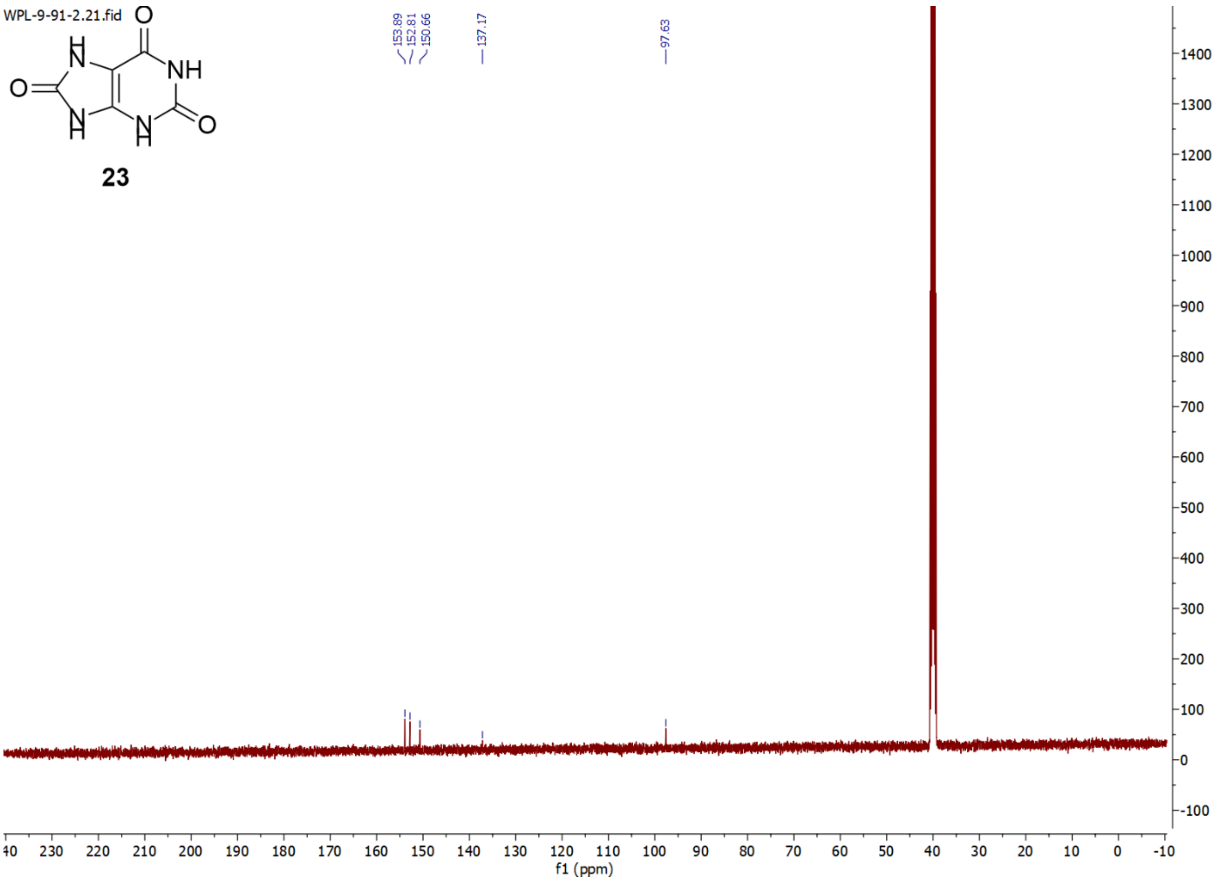

Note Figure 45 | ^{13}C NMR spectrum of product 23.

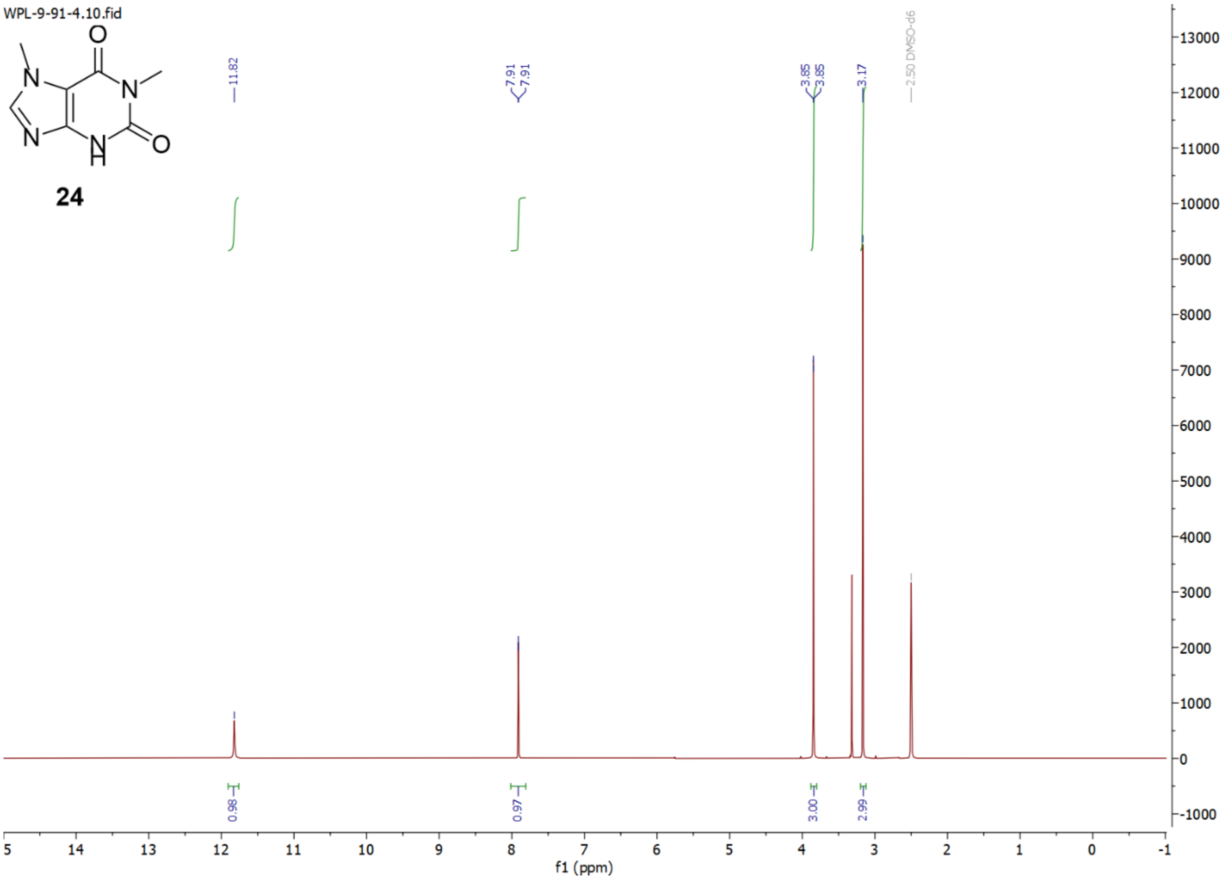

Note Figure 46 | ^{1}H NMR spectrum of product 24.

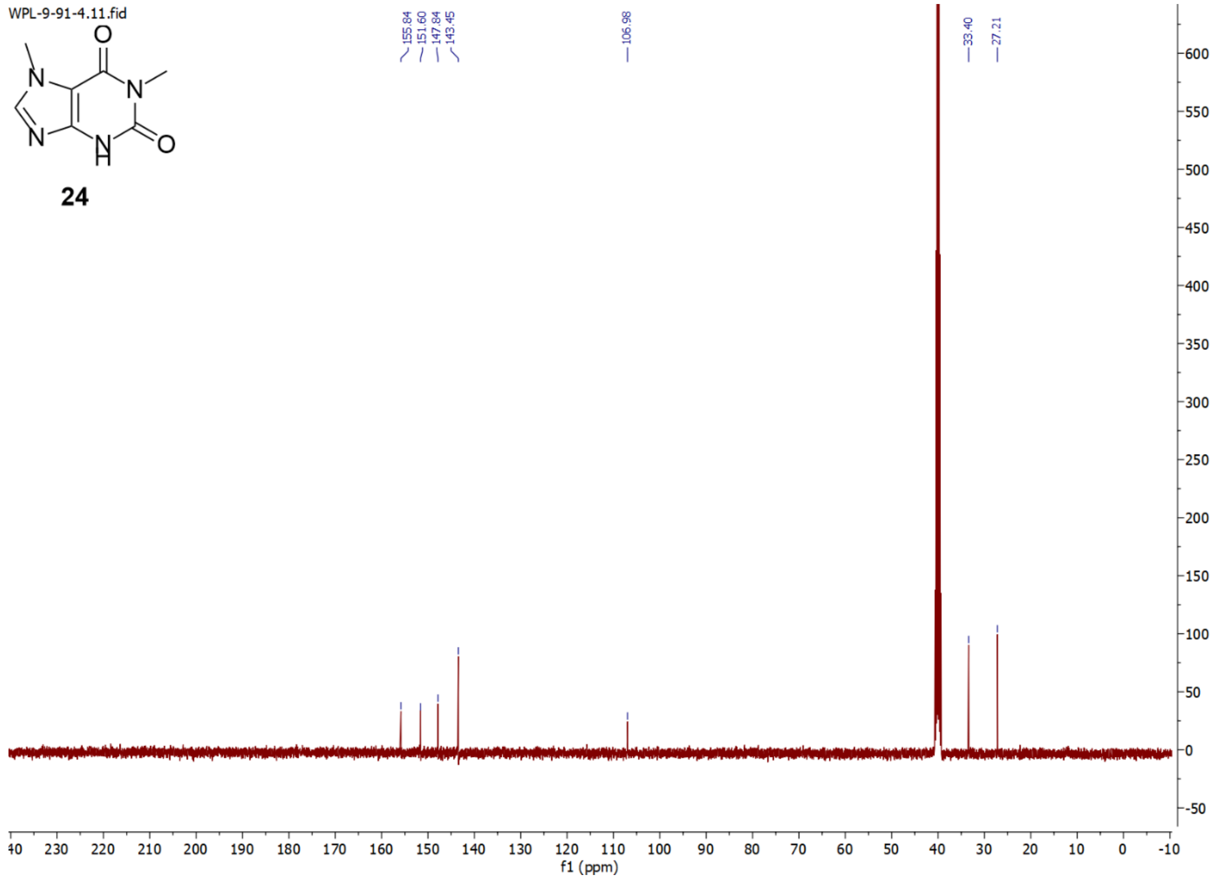

Note Figure 47 | ^{13}C NMR spectrum of product 24.

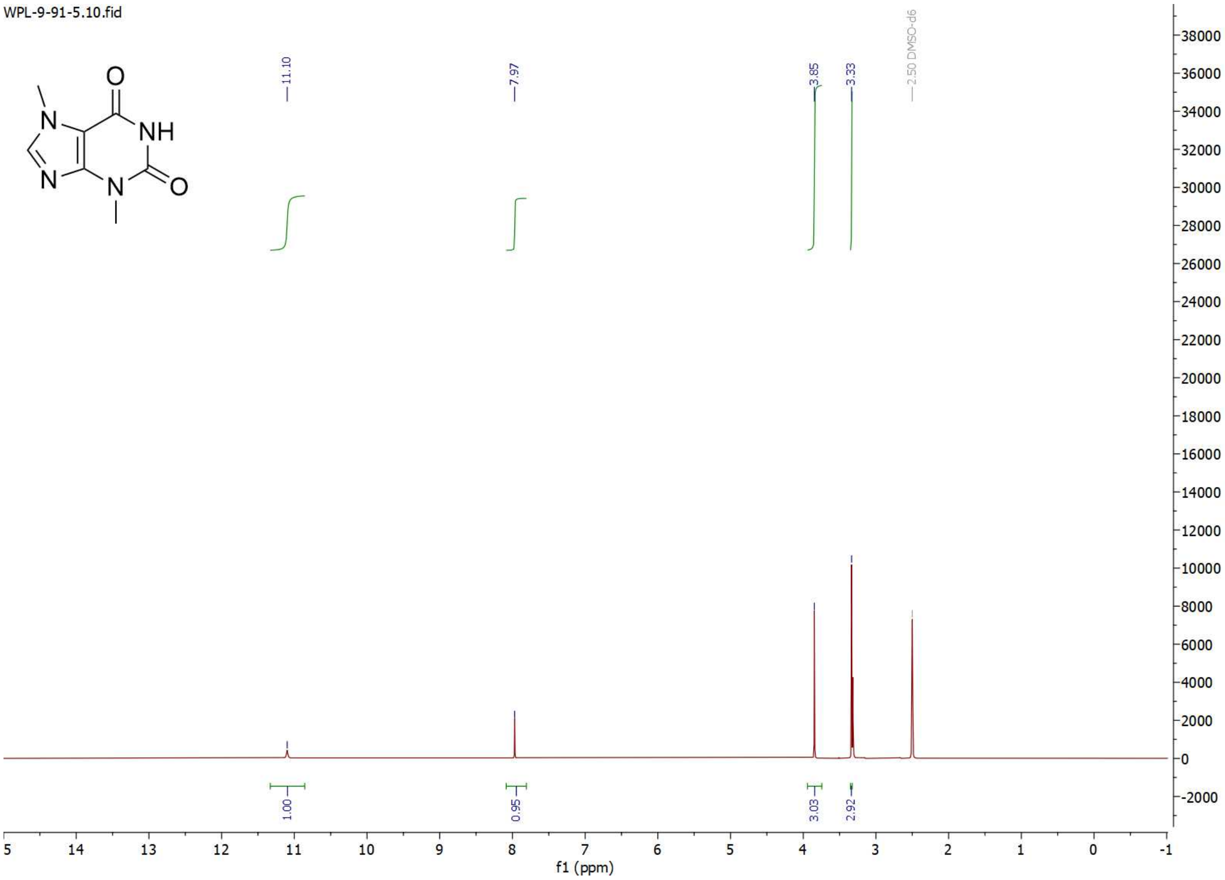

Note Figure 48 | ^{1}H NMR spectrum of product 25.

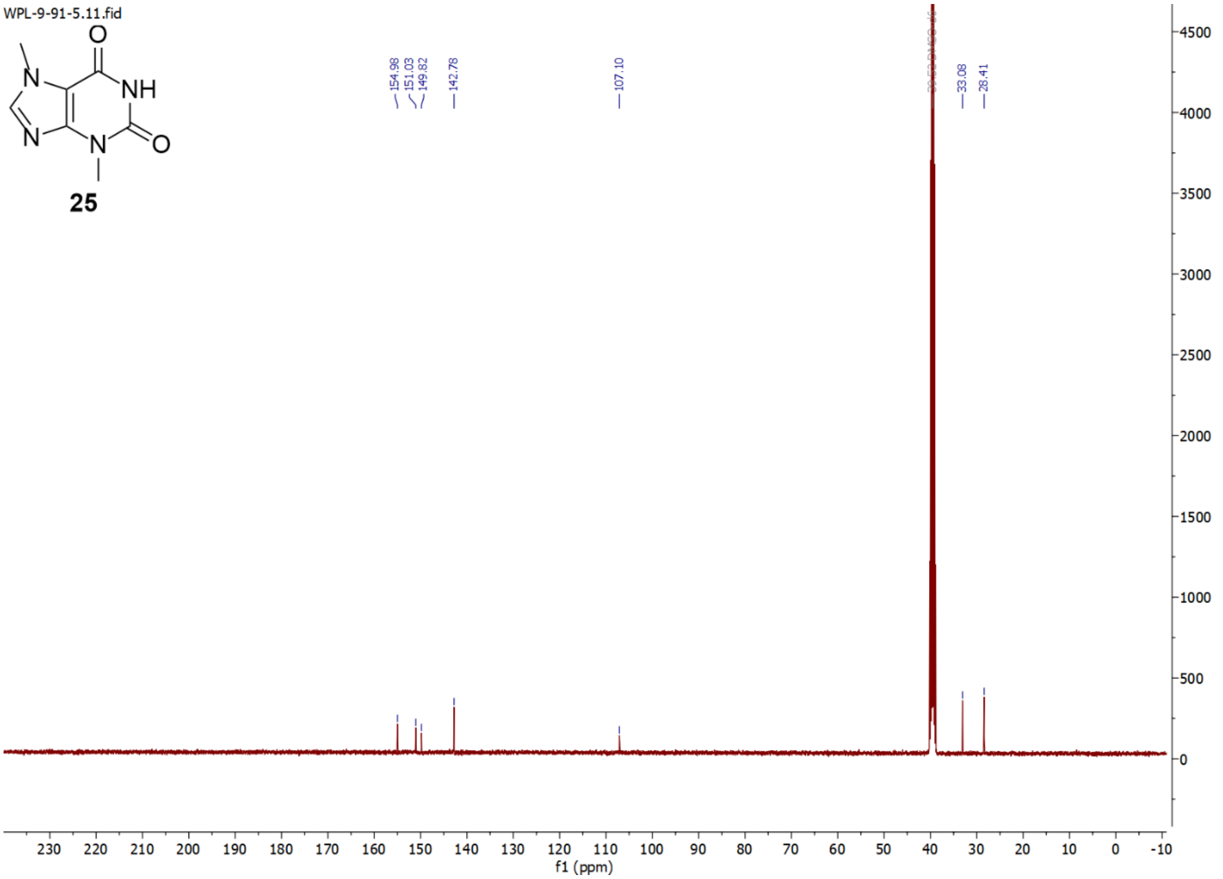

Note Figure 49 | ^{13}C NMR spectrum of product 25.

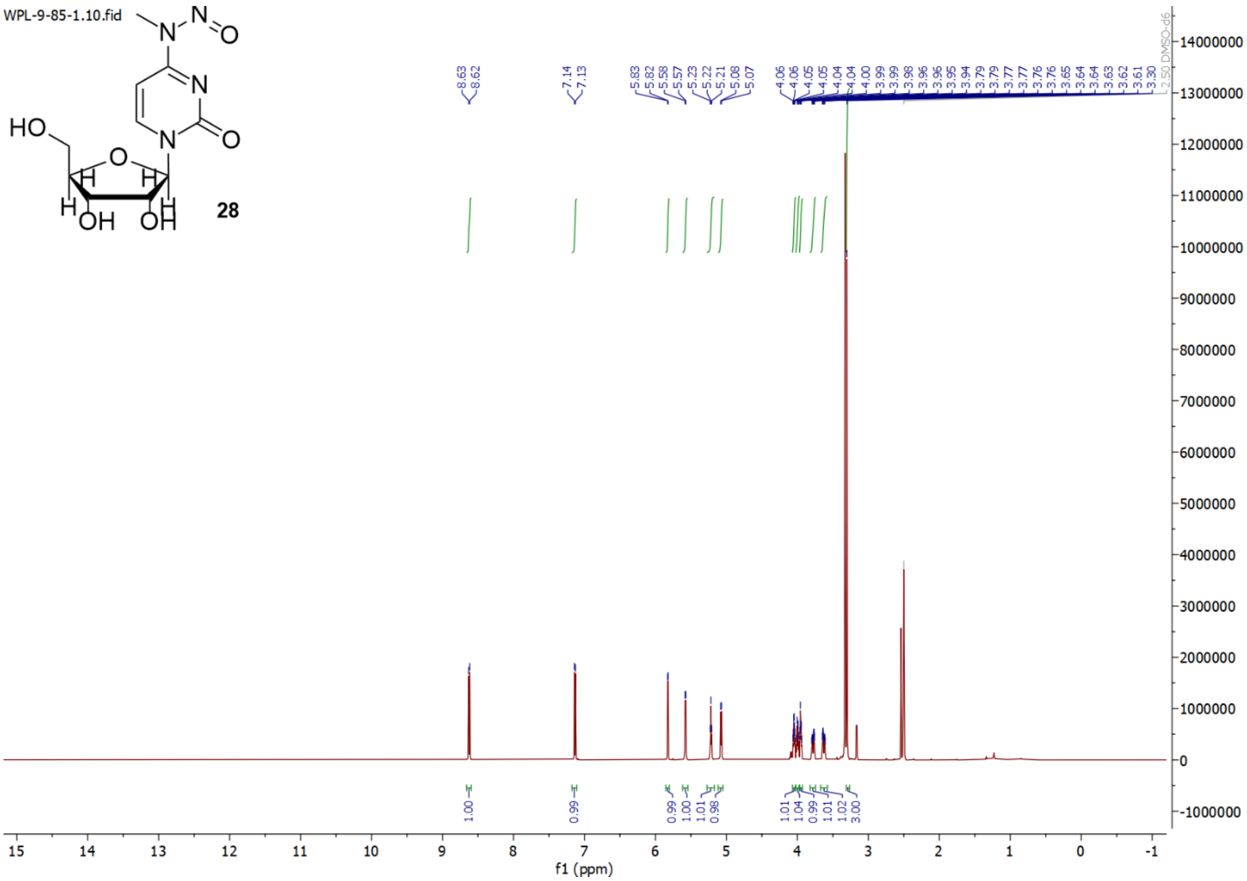

Note Figure 52 | ^{1}H NMR spectrum of product 28.

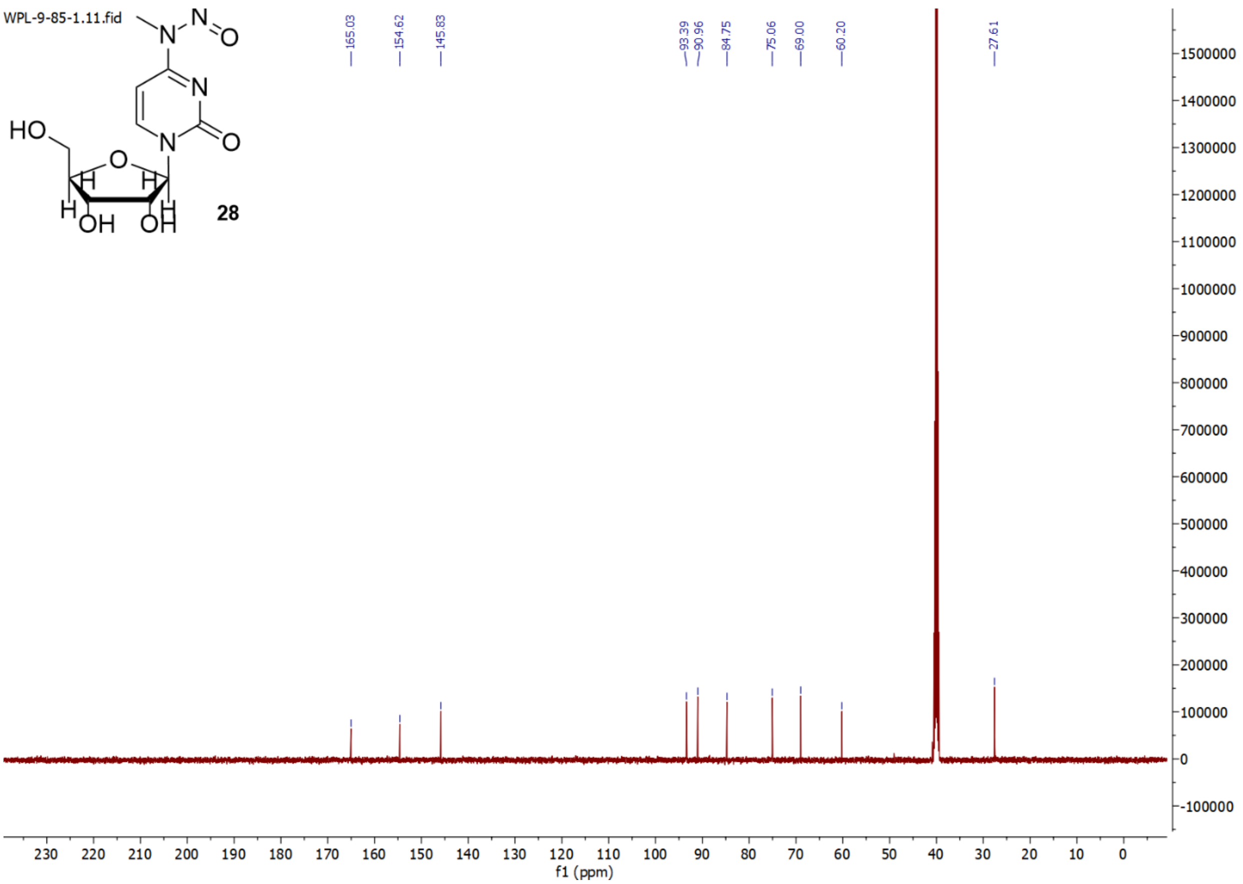

Note Figure 53 | ^{13}C NMR spectrum of product 28.

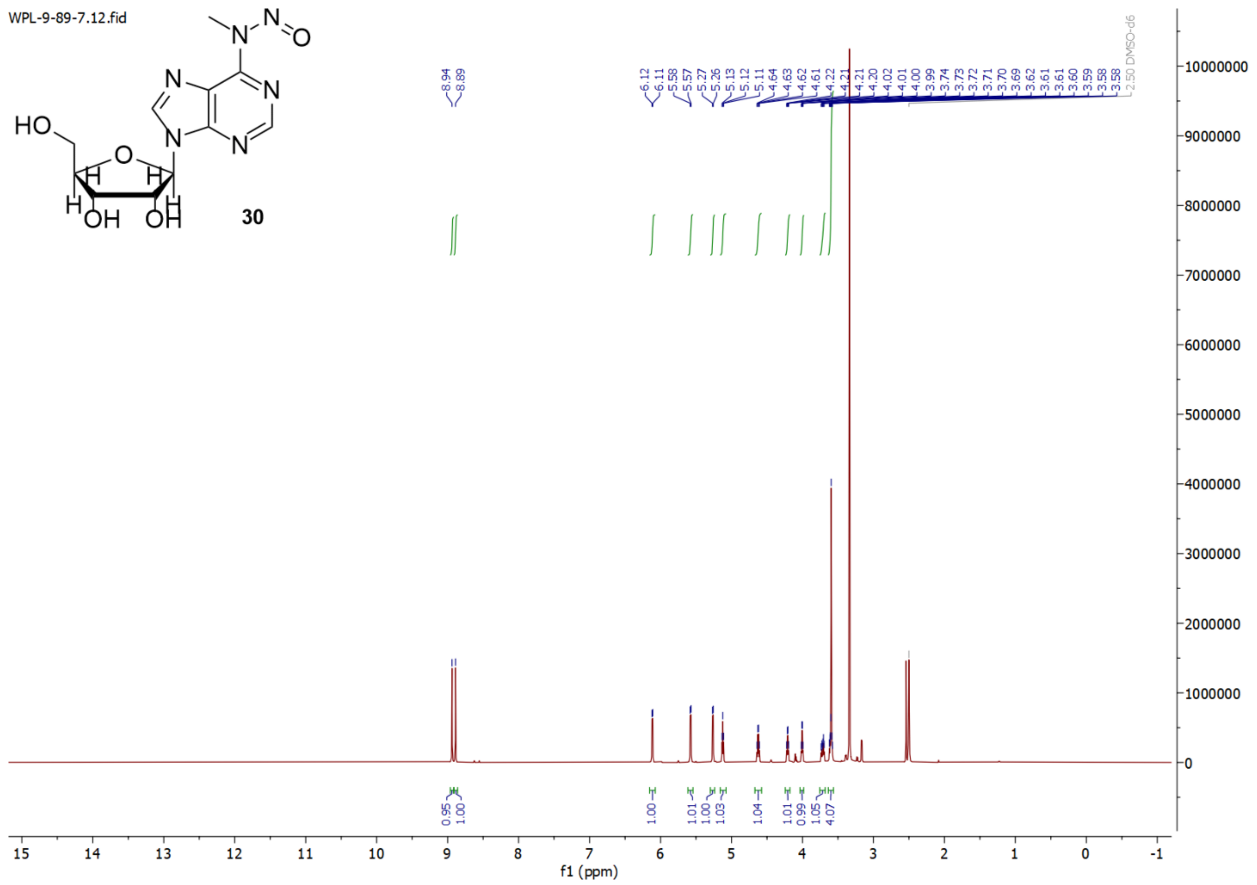

Note Figure 54 | ^{1}H NMR spectrum of product 29.

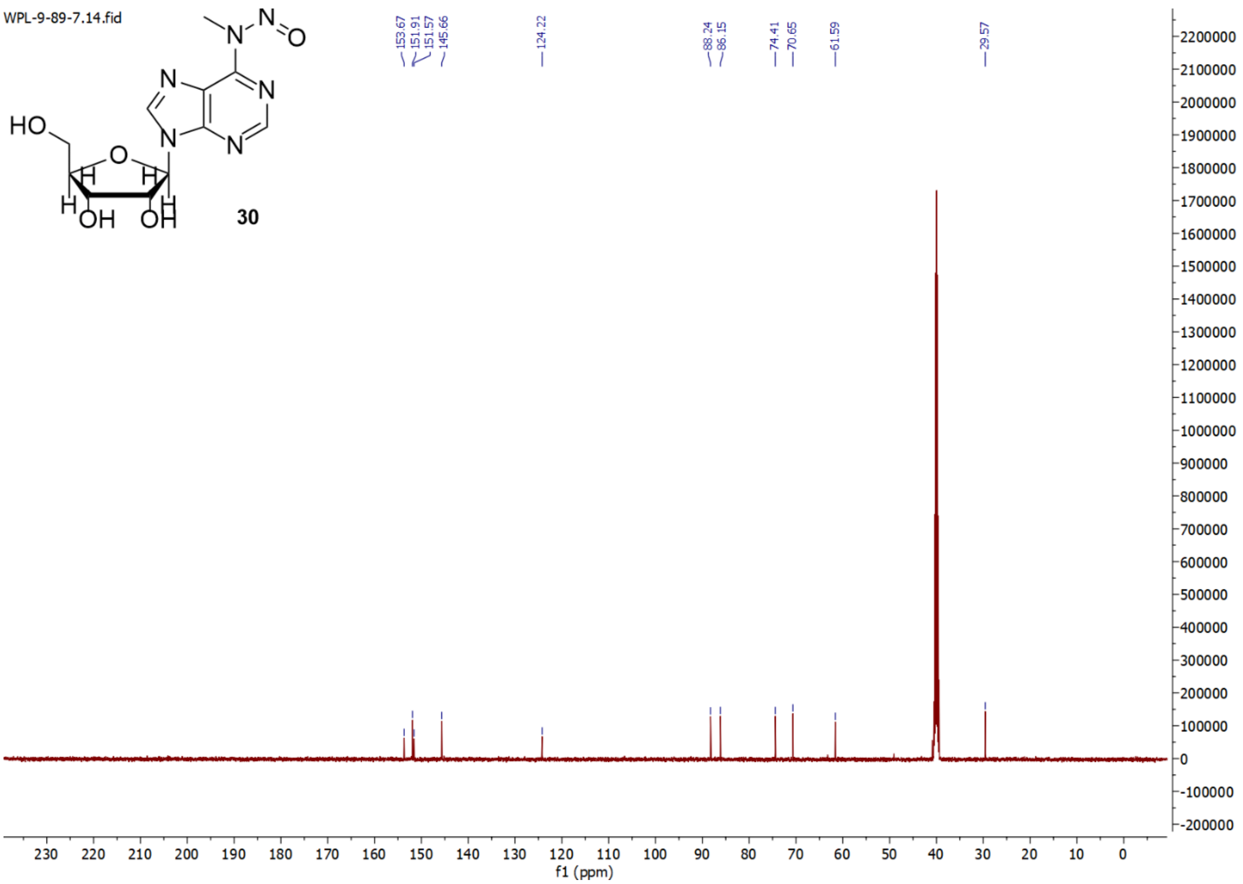

Note Figure 55 | ^{13}C NMR spectrum of product 29.

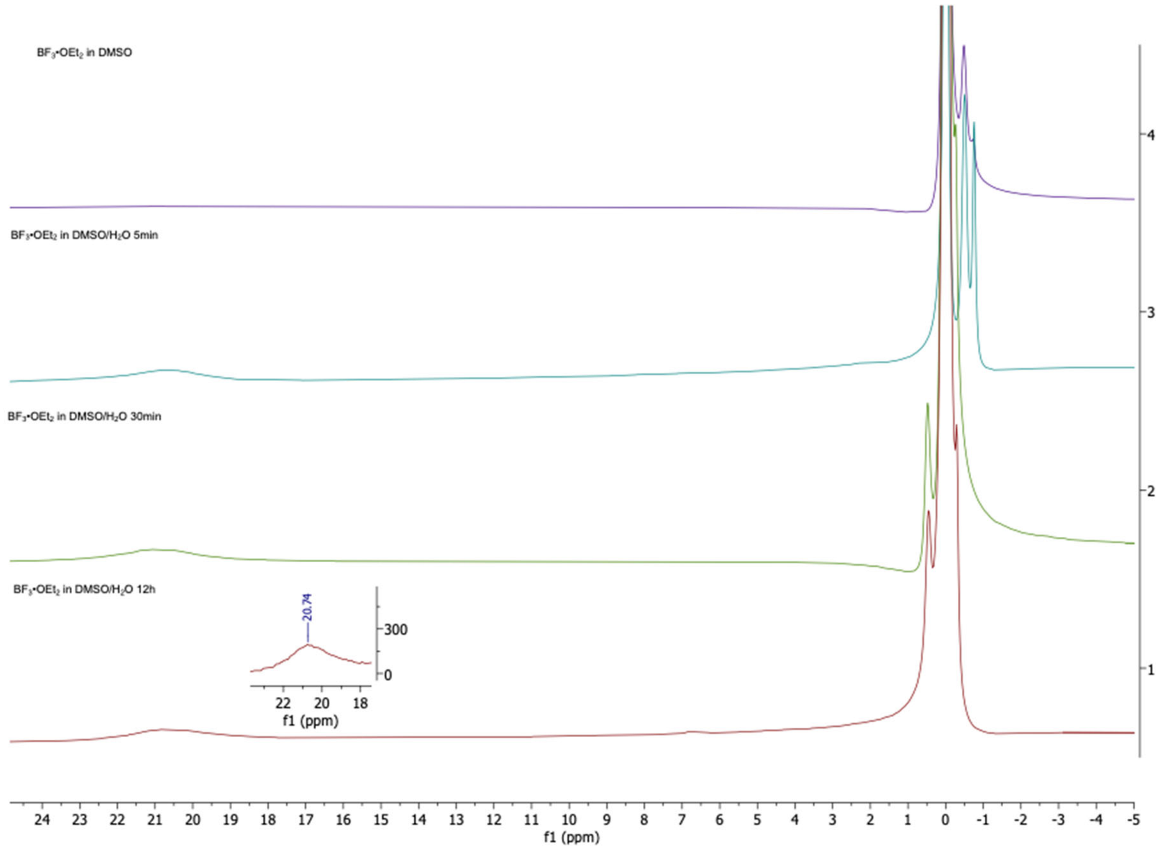

Note Figure 56 | ^{11}B NMR spectrum: B^{11} NMR experiment for BF_3 hydrolysis in mixture solvent.

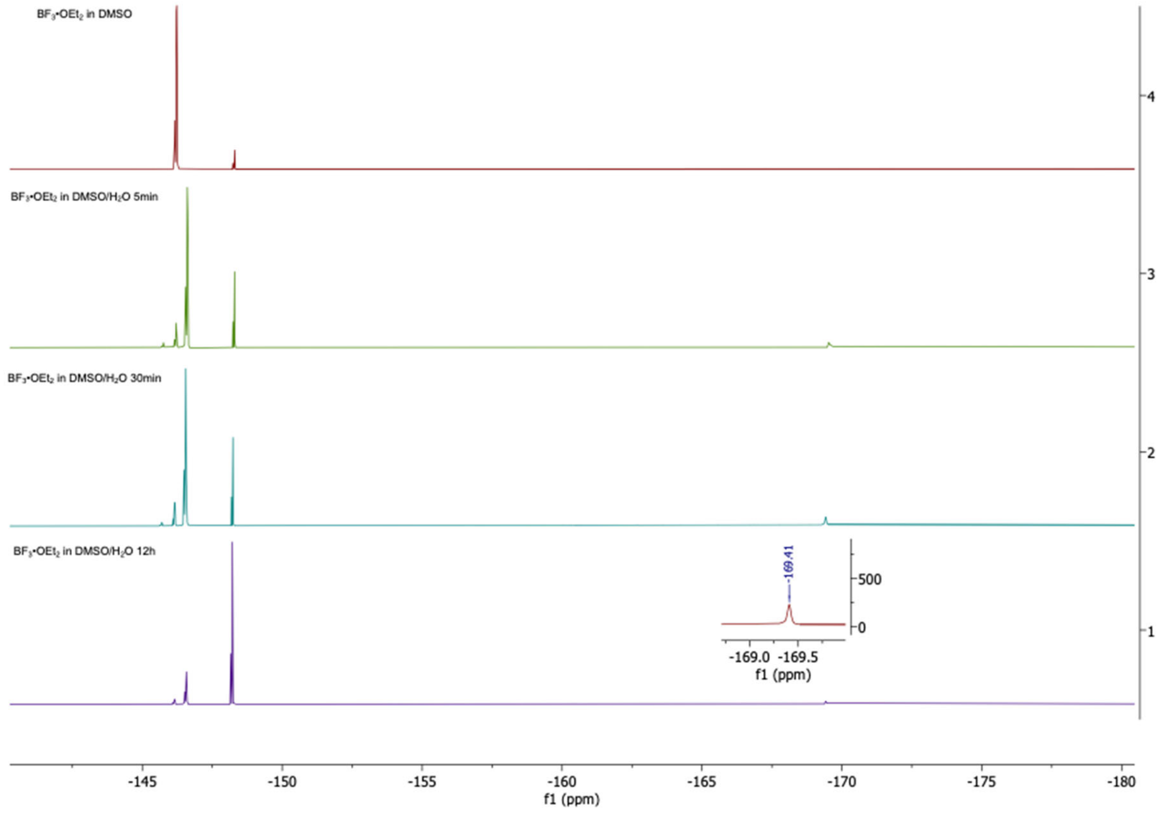

Note Figure 57 | ^{19}F NMR spectrum: F^{19} NMR experiment for BF_3 hydrolysis in mixture solvent.

Supplementary Note II: CAM-seq Protocol

1. **RNA fragmentation.** Timing: ~15 mins

Transfer 5 µL RNA sample (10 ~ 300 ng) to a PCR tube. Add a premixed stock of 2 µL 10× RNA Fragmentation Buffer (NEB, E6150S) and 13 µL RNase-free water to the RNA samples. Mix well by pipetting 10 times.

Prepare RNA fragmentation reaction as follows:

Reagents	Volume (µL)
RNA	5
10× RNA Fragmentation Buffer	2
RNase-free water	13

Note: If there are multiple samples, it is recommended to premix the 10× RNA Fragmentation Reagents and RNase-free water at first, followed by adding 5 µL premixed stock into each sample.

Heat the RNA samples in a PCR block using a preheated, hot-lid thermocycler at 94 °C for **2.5 min** then immediately move onto the ice. (peak at ~300 nt)

Add 2 µL 10× RNA Fragmentation Stop Solution (NEB, E6150S) to the fragmentation reaction.

Purify samples with Zymo RNA Clean & Concentrator-5 kit according to the manufacturer's protocol. Add 42 µL RCC Binding Buffer and mix well. Then add 100 µL EtOH. Mix well by pipetting 20 times. Load the solution onto the RCC column, followed by 400 µL RNA Pre-Wash Buffer and 670 µL Wash Buffer.

Elute RNA with 11 µL RNase-free water to get RNA in 10 µL RNase-free water.

Pause point: The eluted RNA (10 µL) can be stored at –80 °C for a week.

Note: For low input RNA, chemical treatment can proceed directly without RNA purification.

2. **RNA treatment with carbonyl-catalyzed deamination.** Timing: ~6 hours

Prepare a premixed stock containing 24 µL RNase-free water, 1 µL 1M N_3-kethoxal solution (**S1**), and 5 µL 10× PBS buffer (**B1**) in DMSO as a final stock of 30 µL. Mix the stock well by pipetting

20 times. Add the premixed stock to 10 µL of the eluted RNA from **Step 1**. Mix well again by pipetting 20 times.

Prepare deamination reaction as follows:

Reagents	Volume (µL)
RNA (from 0.1 ng to 10 ng)	9
Spike-in mix (0.05% of the input RNA)	1
N_3-kethoxal DMSO solution (**S1**)	1
10× PBS buffer (pH ~7.4) (**B1**)	5
RNase-free water	24

Heat the samples at 70 °C for 2 min; then 37 °C for 30 min; then 4 °C hold.

After finishing the reaction, add 15 µL 8.8 M glyoxal solution (**S2**), and 10 µL H_3BO_3/Na_3BO_3 buffer (saturated, ~500mM, **B2**). Mix the stock well by pipetting 20 times.

Reagents	Volume (µL)
RNA protection reaction solution	40
8.8M glyoxal solution (**S2**)	15
500 mM H_3BO_3/Na_3BO_3 Buffer (pH=6.4) (**B2**)	10

Note: Split the glyoxal solution into multiple tubes and store the stock separately to minimize frequent opening and reduce oxidation.

Heat the samples at 50 °C for 30 min; then hold at 4 °C.

After finishing the reaction, add 10 µL $NaNO_2$ (sat. ~8M, **S3**), 5 µL HEPES buffer (1M, pH = 6.0, **B3**), and 20 µL nuclease-free water. Mix the stock well by pipetting 40 times.

Reagents	Volume (µL)
RNA catalysis reaction solution	65
$NaNO_2$ (Sat.) solution (**S3**)	10
1M HEPES buffer (pH = 7.0) (**B3**)	5
Nuclease-free water	20

Perform PCR on the samples.

**Temp.**	**Time**	**Cycle**
37℃	5 min	5 cycles
18℃	30 min	
4℃	hold	

After finishing the deamination reaction, add 50 µL NH_4Cl (1M, in water, **S4**), 50 µL Tris-HCl buffer (1M, pH = 8.0, **B4**) and **1.0 µL dGTP solution** (100mM in water) to quench the reaction system. Mix the stock well by pipetting 20 times. Purify samples with EtOH precipitation according to the following protocol:

Transfer the quenched solution to a 1.5-mL EP tube and add 600 µL 100% EtOH (3.0x); 20 µL 3M NaOAc, pH = 5.5 (10%); and **3 µL GlycoBlue™ Coprecipitant** (15 mg/mL, Invitrogen™ AM9516). Incubate at -80 °C overnight, then centrifuge at top speed (~13,000 rpm) in a microcentrifuge for 15 min. A significant amount of GlycoBlue Coprecipitant precipitates out the nucleic acid and remains associated with the nucleic acid pellet while washing with 75% ethanol. Collect the RNA pellet and dissolve in 20 µL nuclease-free water.

3. **Deprotection of the chemically treated RNA.** Timing: ~30 min

Prepare a premixed stock containing 100 µL formamide, 100 µL TEAA buffer (1M, pH = 7.0) as the **B5** buffer. Add 5 µL dNTP mix (10 mM each, **S5**), 25 µL **B5** and 5 µL nuclease-free water as a final stock of 50 µL per reaction. Mix the stock well by pipetting 20 times. Add the premixed stock into 20 µL redissolved RNA from **Step 2**. Mix well again by pipetting 20 times.

Prepare RNA deprotection reaction as follows:

Reagents	Volume (µL)
Treated RNA	20
dNTP mix (10 mM each) (**S5**)	5
Formamide & TEAA mix buffer (**B5**)	25

Heat the RNA samples to 94 °C for **5 min** and immediately move onto the ice.

Purify samples with Zymo RNA Clean & Concentrator-5 kit according to the manufacturer's protocol. Add 100 µL RCC Binding Buffer and mix well. Then add 225 µL EtOH (1.5 X). Mix well by pipetting 20 times. Load onto the RCC column, followed by 400 µL RNA Pre-Wash Buffer and 680 µL Wash Buffer.

Elute RNA with 11 µL RNase-free water to get RNA in 10 µL RNase-free water.

4. **RNA end repair.** Timing: ~1.5 hours; **must be in PCR block**.

Prepare a premixed stock containing 5 µL RNase-free water, 2 µL 10× PNK buffer, 1 µL SUPERase•In™, and 2 µL T4 PNK for a final stock of 10 µL per sample. Mix the stock well by pipetting 20 times. Add the premixed stock into 10 µL eluted RNA from **Step 3**. Mix well again by pipetting 20 times.

Prepare the end repair reaction as follows:

Reagents	Volume (µL)
RNA	10
10× PNK reaction buffer	2
SUPERase•In™	1
RNase-free water	5
T4 PNK	2

Heat the samples at 37 °C for 45 min to 1 hour.

Dilute the reaction to 30 µL with nuclease-free water. Purify samples with Zymo RNA Clean & Concentrator-5 kit according to the manufacturer's protocol. Add 60 µL RCC Binding Buffer and mix well. Then add 220 µL EtOH (2.5 X). Mix well by pipetting 20 times. Load onto the RCC column, followed by 400 µL RNA Pre-Wash Buffer and 700 µL Wash Buffer.

Elute RNA with 11 µL RNase-free water to get 3′-repaired RNA in 10 µL RNase-free water.

Pause point: The eluted RNA (10 µL) can be stored at –80 °C for a week.

5. **RNA 3′ adaptor ligation.** Timing: ~12 hours; **Must be in PCR block**.

10 µL of end-repaired RNA solution were mixed with 1.0 µL of 20 µM RNA 3′-Adapter (/5rApp/AGATCGGAAGAGCGTCGTG/3Bio/). Heat at 70 °C for 2 mins and immediately move onto the ice.

Note: After RNA denature, placing the samples immediately onto the ice is important.

Then, prepare a stock containing 2.5 µL 10× T4 RNA Ligase Reaction Buffer, 7.5 µL PEG8000 (50%), and 1.0 µL SUPERase•In. Mix very well by pipetting 30 times.

Add the stock into the RNA–adapter mixture. Mix the samples well by pipetting 10 times. Then add 1.0 µL T4 RNA Ligase 2 truncated KQ. Mix the samples well again by pipetting 20 times.

Reagents	Volume (µL)
RNA and linker mixture	11.0
10× T4 RNA Ligase Reaction Buffer	2.5
50% PEG8000	7.5
SUPERase•In™	1.0
Nuclease-free water	2.0
T4 RNA ligase 2 truncated KQ	1.0

Incubate the reaction at 25°C for 2 hours, followed by 16°C for 12 hours and then 4 °C forever.

After the ligation, dilute the reaction to 47 µl with 22 µL RNase-free water, then add 2 µL 5′-deadenylase. Mix the samples well by pipetting 20 times.

Reagents	Volume (µL)
Reaction Mixture	25
RNase-free water	22
5′-deadenylase	2

Note: To avoid enzyme inactivation, do not pre-mix the 5′-deadenylase with RNase-free water as a stock.

Incubate the samples at 30 °C for 30 min to 1 hour to allow the 5'-deadenylation of excessive adapters.

After the 5'-deadenylation, add 1 µL RecJf for ssDNA digestion. Mix the samples well by pipetting 20 times.

Reagents	Volume (µL)
Reaction Mixture	49
RecJf	1

Incubate the samples at 37 °C for 30 min to 1 hour.

Purify samples with Zymo RNA Clean & Concentrator-5 kit according to the manufacturer's protocol. Transfer each reaction to a new EP tube. Add 100 µL RCC Binding Buffer (2×) and mix well. Then add 170 µL EtOH. Invert 15 times and quickly spin. Load to the RCC column, spin for 1 min, then **change out into a new collection tube**. Then add 400 µL RNA Pre-Wash Buffer and 670 µL Wash Buffer.

Elute RNA with 11 µL RNase-free water to get 3′-end-ligated RNA in 10 µL RNase-free water.

Pause point: The eluted RNA (10 µL) can be stored at –80 °C for a week.

6. **Reverse transcription and RNase H treatment.** Timing: ~1 hour + 30 min; **Must be in PCR block**.

Mix RNA samples with 1.2 µL 2.0 µM CAM-seq RT primer (5′-ACACGACGCTCTTCCGATCT-3′). Heat at 70 °C for 2 mins and immediately move onto the ice.

Note: After RNA denature, placing the samples immediately onto the ice is important.

Prepare a stock with 4 µL 5× reaction Buffer, 3.05 µL dNTP Solution Mix (2.0 µL 100mM dCTP, 0.5 mM 100 mM dATP, 0.5 µL 100 mM dGTP, and 0.05 µL 100 mM dTTP), 1.0 µL RNaseOUT Recombinant Ribonuclease Inhibitor, and 1.0 µL RevertAid H- Reverse Transcriptase per RNA sample. Mix the stock very well by pipetting 20 times. Then add the pre-mixed stock to each sample, and finally mix well by pipetting 20 times.

Reagents	Volume (µL)
RNA-primer mixture	11
5× Reverse transcription reaction Buffer	4
dNTP Solution Mix	3.05
RNaseOUT Recombinant Ribonuclease Inhibitor	1.0
RevertAid H- RT	1

Note: Mixing the stock well is important when working with multiple samples.

Incubate the reaction at 42 °C for 1 h.

Pause point: The RT product can be stored at 4 °C overnight. Storing for a longer time than overnight is not recommended.

After the RT reaction, add 1 µL RNase H. Mix well by pipetting 20 times.

Reagents	Volume (µL)
Reaction Mixture	20
RNase H	1

Incubate at 37 °C for 20 min, then heat at 70 °C for 5 min.

Purify cDNA samples with Zymo **DNA Clean & Concentrator kit** (Not the RCC kit). Add a **7X volume (147 µL)** of **DNA binding buffer** and mix by pipetting 10 times. Transfer to DNA Clean & Concentrator column, and spin. Wash the columns with 200 µL DNA Wash Buffer twice.

Elute the cDNA using 12 µL RNase-free water to get cDNA in 10 µL RNase-free water.

Pause point: The eluted cDNAs were stored at –80 °C for 6 months.

7. cDNA adaptor ligation. Timing: ~12 hours; **Must be in PCR block**.

Mix the purified cDNA with 2.0 µl 20 µM CAM-seq cDNA Adapter (5′-/5Phos/NNNNNNNNNNAGATCGGAAGAGCACACGTCTG/3SpC3/-3′). Heat at 70 °C for 2 mins and immediately move onto the ice.

Note: After cDNA denature, placing the samples immediately onto the ice is important.

Then prepare a stock containing 5 µL 10× T4 RNA Ligase Reaction Buffer, 0.5 µL 100 mM ATP, 25 µL PEG8000 (50%), 1.25 µL $Co(NH_3)_6Cl_3$ (40 mM) and 3.75 µL DMSO. Mix very well by pipetting 30 times.

Add the stock into the RNA–adapter mixture and mix well by pipetting 20 times. Then, add 1 µL T4 RNA Ligase 1 (high concentration) to each sample separately. **Mix the ligase one by one**. Then mix well again by pipetting 20 times.

Reagents	Volume (µL)
cDNA and cDNA-adaptor mixture	13.5
10× T4 RNA Ligase Reaction Buffer	5
50% PEG8000	25
100 mM ATP	0.5
DMSO	3.75
$Co(NH_3)_6Cl_3$ (40 mM)	1.25
T4 RNA Ligase 1 (high concentration)	1

Incubate the reaction at 25°C for 12 hours, then 4 °C forever.

Purify cDNA samples with Zymo **DNA Clean & Concentrator kit** (Not the RCC kit). Add a **7X volume (350 µL)** of **DNA binding buffer** and mix by pipetting 10 times. Transfer to DNA Clean & Concentrator column, and spin. Wash the columns with 200 µL DNA Wash Buffer. Repeat wash once.

Elute using 11 µL nuclease-free water to get adaptor-cDNA in 10 µL nuclease-free water.

Pause point: The eluted cDNAs (10 µL) were stored at –80 °C for 6 months.

8. PCR amplification for NGS sequencing. Timing: ~4 hours; **Must be in PCR block**.

Obtain 1 µL cDNA from **Step 7** was used for qPCR.

Use 8 µL cDNA obtained from **Step 7** for each 13 to 16-cycle PCR amplification reaction. Add 10 µL NEBNext Unique Dual Index Primer for Illumina (10 µM) for Illumina, 25 µL Q5® Hot Start High-Fidelity 2× Master Mix and 7 µL nuclease-free water to each sample.

Note: To avoid mistakes in assigning indexes for multiple samples, it is recommended to add indexed primers into PCR tubes before the addition of cDNA.

Reagents	Volume (µL)
cDNA	8
NEBNext Unique Dual Index Primer for Illumina (10 µM)	10
RNase-free water	7
Q5® Hot Start High-Fidelity 2× Master Mix	25

Perform PCR on the samples from **Step 8**.

**Temp.**	**Time**	**Cycle**
98℃	30 s	
98℃	15 s	13 -16 cycles
60℃	30 s	
68℃	30 s	
4℃	hold	

After PCR amplification, add 10 µL Gel Loading Dye (6X) to each sample and mix well by pipetting 10 times.

Library purification can be achieved using a 3.5% low melting point agarose gel, run at 90 V for 45 mins. pBR322 DNA-MspI Digest (NEB) is used as a size marker.

Recover the DNA from the agarose gel using MinElute Gel Extraction Kit (QIAGEN) according to the manufacturer's protocol. Elute DNA with 15 µL RNase-free water.

Reagents

● Dynabeads™ mRNA DIRECT™ Purification Kit (Invitrogen, cat. no. 61012)

● RNA Clean & Concentrator-5 (Zymo Research, cat. no. R1013)

● DNA Clean & Concentrator-5 (Zymo Research, cat. no. D4013)

● Ethyl alcohol, pure (CH_3CH_2OH; Sigma-Aldrich, cat. no. E7023)

●GlycoBlue™ Coprecipitant (15 mg/mL) (Invitrogen, cat. no. AM9515)

● RNA Fragmentation Reagents (New England BioLabs, cat. no. E6150S)

● Glyoxal solution (Sigma Aldrich, cat. no. 50649-25ML)

● SUPERase·In™ RNase Inhibitor (20 U/µL) (Invitrogen, cat. no. AM2696)

- T4 Polynucleotide Kinase Reaction Buffer (700 mM Tris-HCl, pH 7.6,100 mM $MgCl_2$, 50 mM DTT; included in New England BioLabs, cat. no. M0201L)
- T4 Polynucleotide Kinase (10 U/µL) (New England BioLabs, cat. no. M0201L)
- SUPERase•In RNase Inhibitor (Invitrogen, cat. no. AM2696)
- RevertAid Reverse Transcriptase (Thermo Scientific™, cat. no. EP0441).
- RNaseOUT Recombinant Ribonuclease Inhibitor (Invitrogen, cat. no. 10777019)
- RNase H (New England BioLabs, cat. no. M0297L)
- T4 RNA Ligase 2, truncated KQ (New England BioLabs, cat. no. M0373L)
- T4 RNA Ligase Reaction Buffer (500 mM Tris–HCl, pH 7.5, 100 mM MgCl2, and 10 mM DTT; included in New England BioLabs, cat. nos. M0373L and M0437M)
- PEG 8000 (50% (wt/vol), included in New England BioLabs, cat. nos. M0373L and M0437M)
- 5′ deadenylase (New England BioLabs, cat. no. M0331S)
- RecJf (New England BioLabs, cat. no. M0264L)
- Deoxynucleotide (dNTP) Solution Mix (New England BioLabs, cat. no. N0447L)
- Deoxynucleotide (dNTP) Solution Set (New England BioLabs, cat. no. N0446S)
- T4 RNA Ligase 1 (ssRNA Ligase), High Concentration (New England BioLabs, cat. no. M0437M)
- T4 RNA Ligase 1 (ssRNA Ligase) (New England BioLabs, cat. no. M0204L)
- ATP (100 mM, included in New England BioLabs, cat. no. M0437M)
- Hexammine cobalt (III) chloride (Sigma Aldrich, cat. no. 481521-25G)
- NEBNext® Multiplex Oligos for Illumina® (96 Unique Dual Index Primer Pairs) (New England BioLabs, cat. no. E6440S)
- Q5® Hot Start High-Fidelity 2× Master Mix (New England BioLabs, cat. no. M0494L)
- Agarose (Low-Melting, Nucleic Acid Recovery/Molecular Biology Grade) (Thermo Fisher Scientific, cat. no. BP165-25)
- Gel Loading Dye (6x) (New England Biolabs, cat. no. B7021S)
- pBR322 DNA-MspI Digest (New England Biolabs, cat. no. N3032S)

Reference

1. Knutson, S. D. *et al.* Thermoreversible Control of Nucleic Acid Structure and Function with Glyoxal Caging. *J. Am. Chem. Soc.* **142**, 17766–17781 (2020).
2. Ge, R. *et al.* m6A-SAC-seq for quantitative whole transcriptome m6A profiling. *Nat Protoc* **18**, 626–657 (2023).
3. Ghiazza, C., Wagner, L., Fernández, S., Leutzsch, M. & Cornella, J. Bio-Inspired Deaminative Hydroxylation of Aminoheterocycles and Electron-Deficient Anilines. *Angewandte Chemie International Edition* **62**, e202212219 (2023).
4. Jin, X.-Y., He, Y.-M., Hui, T.-H., Liu, L. & Cheng, L. Selective Methylation of Nucleosides via an In Situ Generated Methyl Oxonium. *J. Org. Chem.* **89**, 3597–3604 (2024).
5. Xiang, L. *et al.* Synthesis and evaluation of NHC derivatives and 4′-fluorouridine prodrugs. *Org. Biomol. Chem.* **21**, 2754–2767 (2023).
6. Wang, X.-K., Jia, Y.-M., Li, Y.-X. & Yu, C.-Y. Total Synthesis of Pseudouridimycin. *Org. Lett.* **24**, 511–515 (2022).
7. Yuan, Y., Tian, J.-M., Xiao, J., Shao, Q. & Gao, J.-M. Bioactive metabolites isolated from Penicillium sp. YY-20, the endophytic fungus from Ginkgo biloba. *Natural Product Research* **28**, 278–281 (2014).
8. Mou, Q. *et al.* DNA Trojan Horses: Self-Assembled Floxuridine-Containing DNA Polyhedra for Cancer Therapy. *Angewandte Chemie International Edition* **56**, 12528–12532 (2017).
9. Baraniak, D., Ruszkowski, P., Baranowski, D., Framski, G. & Boryski, J. Nucleoside dimers analogs containing floxuridine and thymidine with unnatural linker groups: synthesis and cancer line studies. Part III. *Nucleosides, Nucleotides & Nucleic Acids* **38**, 980–1005 (2019).

10. Hansen, A. S., Thalhammer, A., El-Sagheer, A. H., Brown, T. & Schofield, C. J. Improved synthesis of 5-hydroxymethyl-2′-deoxycytidine phosphoramidite using a 2′-deoxyuridine to 2′-deoxycytidine conversion without temporary protecting groups. *Bioorganic & Medicinal Chemistry Letters* **21**, 1181–1184 (2011).

11. Guo, P. *et al.* Synthesis and spectroscopic properties of fluorescent 5-benzimidazolyl-2′-deoxyuridines 5-fdU probes obtained from o-phenylenediamine derivatives. *Org. Biomol. Chem.* **11**, 1610–1613 (2013).

12. Bednarek, E. *et al.* Theoretical and experimental 1H, 13C, 15N, and 17O NMR spectra of 5-nitro, 5-amino, and 5-carboxy uracils. *Journal of Molecular Structure* **482**, 333–337 (1999).

13. Rosemeyer, H. *et al.* Syn-anti conformational analysis of regular and modified nucleosides by 1D 1H NOE difference spectroscopy: a simple graphical method based on conformationally rigid molecules. *J. Org. Chem.* **55**, 5784–5790 (1990).

14. Zheltovsky, N. V., Samoilenko, S. A., Kondratyuk, I. V., Kolomiets, I. N. & Stepanyugin, A. V. Recognition of purine bases and nucleosides by the amino acid carboxylic group. *Journal of Molecular Structure* **344**, 53–62 (1995).

15. Narukulla, R., Shuker, D. E. G., Ramesh, V. & Xu, Y.-Z. Unambiguous structural elucidation of base-modified purine nucleosides using NMR. *Magnetic Resonance in Chemistry* **46**, 1–8 (2008).

16. Londregan, A. T., Curto, J. M., Hastry, E., Rose, C. R. & Berritt, S. Preparation of Azinones from (Cyclopropylmethoxy)azine Ethers. *J. Org. Chem.* **88**, 5671–5675 (2023).

17. Rosemeyer, H., Kaiser, K. & Seela, F. Spontaneous hydroxylation of a cyclization intermediate of allopurinol. *J. Org. Chem.* **50**, 1847–1852 (1985).

18. Wright, A. E. *et al.* Isolation, Synthesis, and Biological Activity of Aphrocallistin, an Adenine-Substituted Bromotyramine Metabolite from the Hexactinellida Sponge Aphrocallistes beatrix. *J. Nat. Prod.* **72**, 1178–1183 (2009).

19. Pochet, S. & Dugué, L. Oligodeoxynucleotides Embodying the Ambiguous Base Z, 5-Amino-imidazole-4-carboxamide. *Nucleosides and Nucleotides* **14**, 1195–1210 (1995).

20. Mueller, C. E., Shi, D., Manning, M. Jr. & Daly, J. W. Synthesis of paraxanthine analogs (1,7-disubstituted xanthines) and other xanthines unsubstituted at the 3-position: structure-activity relationships at adenosine receptors. *J. Med. Chem.* **36**, 3341–3349 (1993).

21. Palazzolo, A. *et al.* Efficient Access to Deuterated and Tritiated Nucleobase Pharmaceuticals and Oligonucleotides using Hydrogen-Isotope Exchange. *Angewandte Chemie International Edition* **58**, 4891–4895 (2019).

22. Weng, X. *et al.* Keth-seq for transcriptome-wide RNA structure mapping. *Nature Chemical Biology* **16**, 489–492 (2020).
